# Eight new *α*-pyrone and *γ*-butenolide derivatives from the plant endophytic fungus *Diaporthe* sp. CCY4

**DOI:** 10.1007/s13659-025-00580-1

**Published:** 2026-02-03

**Authors:** Jie-Chun Zeng, Xu-Ping Zhang, Lu Gao, Qian-Qian Yin, Wei-Guang Wang

**Affiliations:** Key Laboratory of Chemistry in Ethnic Medicinal Resources of Ministry of Education, Yunnan Minzu University, Kunming, 650031 Yunnan People’s Republic of China

**Keywords:** *α*-pyrone, *γ*-butenolide, *Diaporthe* sp., USP4, Polyketides, Deubiquitinating enzymes

## Abstract

Five new *α*-pyrones, diaporpyrones G-K (**1**–**5**) and three new *γ*-butenolide derivatives, porbutenolides A-C (**6**–**8**), along with seven known compounds (**9**–**15**), were isolated from the culture extract of the endophytic fungus *Diaporthe* sp. CCY4. Their structures were elucidated by comprehensive spectroscopic analysis, including 1D/2D NMR and HRESIMS data. The absolute configurations of **7** and **8** were assigned using electronic circular dichroism (ECD) calculations. All compounds were evaluated for inhibitory activity against ubiquitin-specific peptidase 4 (USP4). Compounds **2**,** 5**,** 9**, and **13** exhibited significant anti-ubiquitination effects at 40 μM, with **13** showing the most potent inhibition (IC_50_ = 20.85 μM).

**Graphical Abstract:**

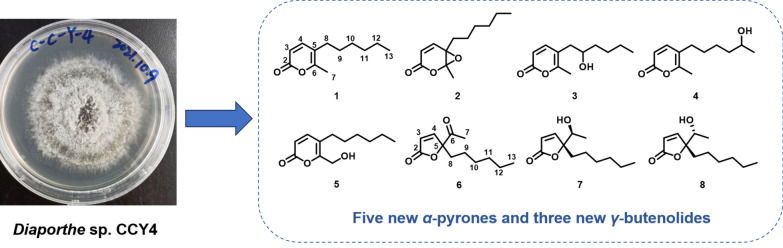

**Supplementary Information:**

The online version contains supplementary material available at 10.1007/s13659-025-00580-1.

## Introduction

Deubiquitinating enzymes (DUBs) are crucial regulators of protein ubiquitination, impacting fundamental processes like signal transduction and protein homeostasis. Dysregulation of DUB activity is implicated in various pathologies, including cancer and neurodegenerative diseases [1–6]. Among DUBs, ubiquitin-specific protease 4 (USP4) modulates multiple signaling pathways via specific substrate interactions. Aberrant USP4 activity is linked to tumor progression, metastasis, and immune evasion [7–10], highlighting its potential as a therapeutic target for cancer.

Current USP4 inhibitor development relies heavily on synthetic compounds (e.g., PR-619). However, issues such as lack of specificity and cytotoxicity limit their clinical utility [11, 12]. In contrast, natural products, shaped by evolutionary pressures, possess diverse and complex structures enabling specific target binding. This makes them invaluable sources for drug discovery [13, 14]. Fungi, renowned as prolific producers of bioactive metabolites with novel scaffolds and precise bioactivities (e.g., penicillin, lovastatin) [15, 16], represent a promising resource for identifying specific USP4 inhibitors. For instance, vialinin A, isolated from the fungus *Thelephora vialis*, exhibits USP4 inhibition and antitumor potential [13, 17, 18].

Among fungal metabolites, α-pyrone and *γ*-butenolide (furanone) derivatives constitute two widely distributed and structurally privileged classes of polyketide-derived natural products. Their chemical versatility and biological relevance have attracted significant interest. α-Pyrones, for example, readily participate in Diels–Alder transformations to generate diverse natural-product-like scaffolds [19], while chromene–pyrone hybrids from endolichenic fungi have been identified as potential plant-growth regulators, highlighting their agrochemical importance [20]. Meanwhile, γ-butenolide and naphthoquinone derivatives from endophytic fungi often exhibit notable bioactivities [21, 22], including pro-apoptotic effects mediated through pathways such as EGFR–PI3K/Akt signaling [21], further underscoring the therapeutic value of these scaffolds.

**Fig. 1:**
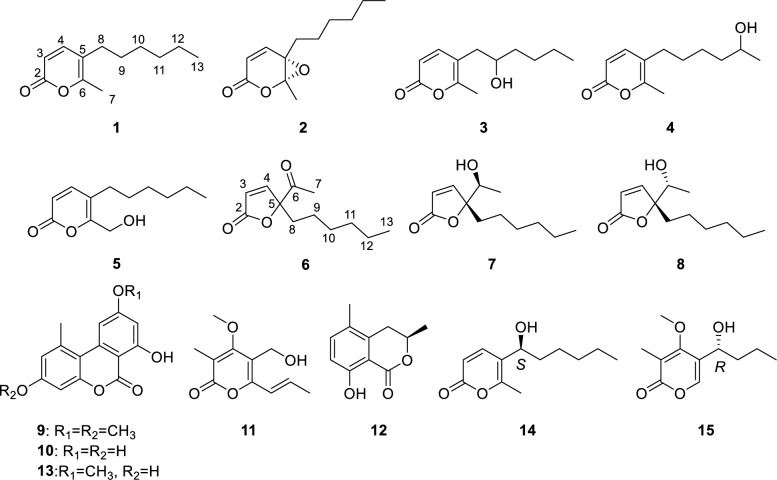
Structures of compounds **1**–**15**

To expand the repertoire of fungal-derived USP4 inhibitors, we conducted a systematic chemical investigation of the endophytic fungus *Diaporthe* sp. CCY4, isolated from *Camellia japonica*. This study led to the isolation of five new α-pyrones, diaporpyrones G-K (**1**–**5**), three new *γ*-butenolides, porbutenolides A-C (**6**–**8**), and seven known compounds (**9**–**15**) (Fig. 1). All isolates were evaluated for USP4 inhibitory activity, with several exhibiting significant effects. Herein, we describe the fermentation, isolation, structural characterization, and biological evaluation of these metabolites.

## Results and discussion

**Fig. 2:**
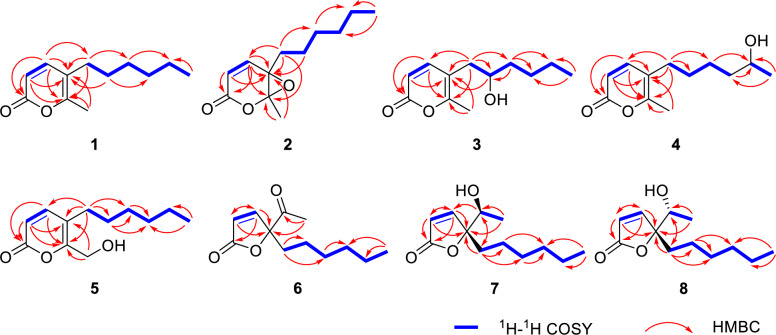
Key ^1^H − ^1^H COSY and HMBC correlations of compounds **1**−**8**

Compound **1**, a yellow oily substance, was assigned the molecular formula C_12_H_18_O_2_ by HRESIMS data (*m*/*z* 195.1377 [M + H]^+^, calcd 195.1379 for C_12_H_19_O_2_), indicating four degrees of unsaturation. The ^1^H NMR spectrum of **1** (Table 1) displayed one methyl singlets (*δ*_H_ 2.15, 3H, s), one methyl doublet (*δ*_H_ 0.81, 3H, d, *J* = 7.0 Hz), along with a set of olefinic protons (*δ*_H_ 7.11, 1H, d, *J* = 9.4 Hz; 6.28, 1H, d, *J* = 9.4 Hz). The ^13^C NMR spectrum of **1** (Table 2) showed 12 carbon signals, including two methyls (*δ*_C_ 17.1 and 14.0), five methylenes (*δ*_C_ 31.5, 29.7, 29.4, 28.6 and 22.5), two methines (*δ*_C_ 147.1 and 113.1), and three nonprotonated carbons (*δ*_C_ 162.9, 158.2 and 115.5) based on DEPT and HSQC analysis. The above NMR spectra of compound **1** was like those of 5-(1-hydroxyhexyl)-6-methyl-2*H*-pyran-2-one (**14)** [23], except for the absence of a hydroxy at C-8. The key difference was supported by ^1^H-^1^H COSY correlations of H_2_-8/H_2_-9/H_2_-10/H_2_-11/H_2_-12/H_3_-13 (Fig. 2). Additionally, the key HMBC correlations (Fig. 2) from H-4 to C-2, C-5, C-6 and C-8, H_3_-7 to C-5 and C-6, and H_2_-8 to C-4, C-5 and C-6 confirmed the structure of *α*-pyranone skeleton. Thus, the structure of compound **1** was determined and named as diaporpyrone G.

**Table 1:** ^1^H (600 MHz) NMR data for compounds **1−5** measured in CDCl_3_

No	**1**	**2**	**3**	**4**	**5**
3	6.06, d (9.4)	6.12, d (10.0)	6.10, d (9.4)	6.13, d (9.4)	6.22, d (9.5)
4	7.11, d (9.4)	7.14, d (10.0)	7.23, d (9.4)	7.15, d (9.4)	7.20, d (9.5)
7	2.15, s	1.79, s	2.24, s	2.21, s	4.41, s
8	2.21, m	1.87, ddd (14.0, 10.0, 5.5) 1.61, ddd (14.0, 10.0, 6.6)	2.45, dd (14.4,4.0) 2.37, dd (14.4,8.5)	2.29, t (7.5)	2.33, t (7.8)
9	1.38, dt (15.3, 7.7)	1.47, dtt (13.5, 10.0, 6.5)	3.69, s	1.46, m	1.44, m
10	1.21, m	1.36, m	1.48, dddd (9.2, 6.9, 4.8, 2.1)	1.34, ddd (10.1, 7.4, 5.3) 1.46, m	1.26, m
11	1.21, m	1.30, m	1.45, m 1.35, m	1.46, m	1.26, m
12	1.21, m	1.30, m	1.32, m	3.79, m	1.26, m
13	0.81, t (7.0)	0.89, t (6.9)	0.90, t (7.2)	1.18, d (6.2)	0.83, t (6.6)
–OH			2.08, s		3.97, br. s

**Table 2:** ^13^C (150 MHz) NMR data for compounds **1−5** measured in CDCl_3_

No	**1**	**2**	**3**	**4**	**5**
2	162.9, C	160.5, C	163.0, C	163.0, C	162.7, C
3	113.1, CH	123.8, CH	113.0, CH	113.4, CH	115.4, CH
4	147.1, CH	147.9, CH	148.0, CH	147.1, CH	147.4, CH
5	115.5, C	58.8, C	112.8, C	115.4, C	117.0, C
6	158.2, C	89.3, C	159.8, C	158.4, C	158.2, C
7	17.1, CH_3_	17.5, CH_3_	17.8, CH_3_	17.3, CH_3_	58.6, CH_2_
8	29.4, CH_2_	30.8, CH_2_	37.4, CH_2_	29.6, CH_2_	28.8, CH_2_
9	29.7, CH_2_	25.0, CH_2_	71.7, CH	29.9, CH_2_	30.3, CH_2_
10	28.6, CH_2_	29.2, CH_2_	37.0, CH_2_	25.4, CH_2_	28.7, CH_2_
11	31.5, CH_2_	31.7, CH_2_	28.0, CH_2_	39.0, CH_2_	31.6, CH_2_
12	22.5, CH_2_	22.6, CH_2_	22.8, CH_2_	68.0, CH	22.6, CH_2_
13	14.0, CH_3_	14.1, CH_3_	14.1, CH_3_	23.8, CH_3_	14.1, CH_3_

Compound **2**, a colorless oil, was assigned the molecular formula C_12_H_18_O_3_ by HRESIMS data (*m*/*z* 211.1329 [M + H]^+^, calcd 211.1328 for C_12_H_19_O_3_), indicating four degrees of unsaturation. The NMR spectra showed that compounds **2** and **1** were similar, with the only differences of the shifts of C-5 and C-6 (Tables 1 and 2). The shifts of C-5 and C-6 and HRESIMS data showed that compound **2** bears one epoxy bond fused to the α-pyranone at C-5 and C-6. Moreover, the HMBC correlations from H_3_-7 to C-5 and C-6 further confirmed the presence of the epoxide moiety. Finally, structure of **2** was determined and named as diaporpyrone H.

Diaporpyrone I (**3)** and diaporpyrone J (**4)** had the same molecular formula of C_12_H_18_O_3_ which was determined by HRESIMS data (**3**: *m*/*z* 211.1331 [M + H]^+^, calcd 211.1328 for C_12_H_19_O_3_; **4**: *m*/*z* 211.1328 [M + H]^+^, calcd 211.1328 for C_12_H_19_O_3_). Their NMR spectra were like those of 5-(1-hydroxyhexyl)-6-methyl-2*H*-pyran-2-one (**14**) [23]. Further ^1^H-^1^H COSY and HMBC correlations revealed that compounds **3** and **4** were positional isomers of compound **14** with respect to the hydroxyl substitution pattern. Specifically, the hydroxyl group in compound **3** was determined to be located at the C-9 position based on the key HMBC correlation of H-9 to C-5, whereas in compound **4** it was found at the C-12, as supported by the key ^1^H-^1^H COSY correlation of H_3_-13 and H-12.

Diaporpyrone K (**5)**, a yellow oily substance, was assigned the molecular formula C_12_H_18_O_3_ by HRESIMS data (*m/z* 211.1326 [M + H]^+^, calcd 211.1328 for C_12_H_19_O_3_), indicating four degrees of unsaturation. The NMR data of compound **5** were highly similar to those of compound **1**, differing only at the C-7 position. Compared to compound **1**, compound **5** contained one fewer methyl group and one additional hydroxymethyl group. Analysis of the HMBC correlation signals between H-7 and both C-5 and C-6 further confirmed that the C-7 position in compound **5** was substituted by a hydroxymethyl group.

Porbutenolide A (**6**) was obtained as yellow oily substance with the molecular formula C_12_H_18_O_3_ deduced from HRESIMS peak at *m/z* 233.1145 [M + Na]^+^, indicating four degrees of unsaturation. The ^1^H NMR spectroscopic data (Table 3) contained signals for a set of olefinic protons (*δ*_H_ 7.32, 1H, d, *J* = 5.4 Hz; 6.15, 1H, d, *J* = 5.4 Hz), and two methyl groups (*δ*_H_ 2.21, 3H, s; 0.85, 3H, t, *J* = 6.9 Hz). The ^13^C NMR spectrum (Table 3) consisted of three quaternary carbons (*δ*_C_ 205.0, 172.2 and 96.4), two methine carbons (*δ*_C_ 155.5, and 122.0), five methylene carbon (*δ*_C_ 35.3, 31.5, 29.2, 23.2 and 22.6), and two methyl carbons (*δ*_C_ 26.3 and 14.1), with DEPT and HSQC analyses. In the ^1^H-^1^H COSY spectrum, the cross-peaks of H_2_-8/H_2_-9/H_2_-10/H_2_-11/H_2_-12/H_3_-13 confirmed the presence of a hexane chain. A *γ*-butenolide ring was constructed on the basis of HMBC correlations from H-3 to C-5, and H-4 to C-2. Furthermore, the key HMBC correlations from H_3_-7/H_2_-8 to C-5 and C-6 confirmed that an acetyl and hexane chain were linked at C-5. Therefore, the planar structure of compound **6** was determined.

**Table 3:** ^1^H (600 MHz) and ^13^C (150 MHz) NMR data for compounds **6 − 8** measured in CDCl_3_

No	**6**		**7**		**8**	
	*δ*_H_ (*J* in Hz)	*δ*_C_	*δ*_H_ (*J* in Hz)	*δ*_C_	*δ*_H_ (*J* in Hz)	*δ*_C_
2		172.2, C		172.8, C		172.7, C
3	6.15, d (5.4)	122.0, CH	6.14, d (5.7)	122.8, CH	6.13, d (5.7)	122.6, CH
4	7.32, d (5.4)	155.5, CH	7.35, d (5.7)	156.8, CH	7.34, d (5.7)	157.6, CH
5		96.4, C		94.1, C		93.9, C
6		205.0, C	3.96, m	70.6, CH	3.89, m	71.3, CH
7	2.21, s	26.3, CH_3_	1.22, d (6.4)	18.2, CH_3_	1.22, d (6.4)	17.9, CH_3_
8	2.10, ddd (14.0, 11.3, 4.4) 1.80, m	35.3, CH_2_	1.91, td (13.1, 11.8, 3.7) 1.79, ddd (15.5, 10.3, 3.1)	33.0, CH_2_	1.99, td (13.1, 11.8, 3.7) 1.78, ddd (14.2, 11.3, 4.9)	31.8, CH_2_
9	1.25, m	22.6, CH_2_	1.25, m 1.14, qd (13.1, 5.8)	23.0, CH_2_	1.26, m 1.08, qd (12.4, 8.2)	22.9, CH_2_
10	1.25, m	29.2, CH_2_	1.25, m	29.5, CH_2_	1.26, m	29.5, CH_2_
11	1.25, m	31.5, CH_2_	1.25, m	31.7, CH_2_	1.26, m	31.7, CH_2_
12	1.25, m	23.2, CH_2_	1.25, m	22.6, CH_2_	1.26, m	22.6, CH_2_
13	0.85, t (6.9)	14.1, CH_3_	0.86, t (7.0)	14.2, CH_3_	0.86, t (7.0)	14.2, CH_3_
-OH			1.96, d (6.0)		1.90, d (5.2)	

**Fig. 3:**
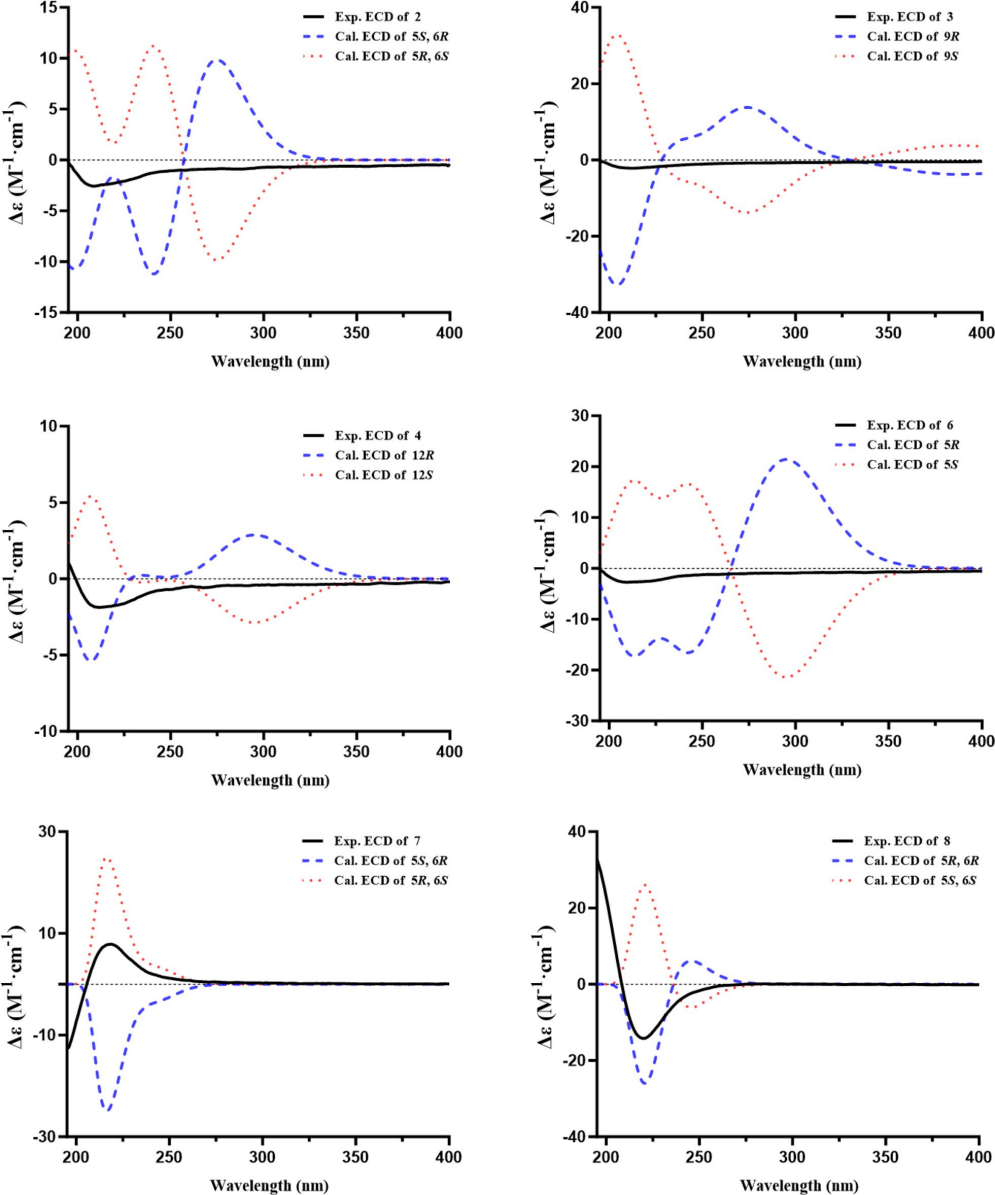
Experimental and calculated CD spectra of **2**−**4**, and **6**−**8**

Porbutenolide B (**7)** and porbutenolide C (**8)** had the same molecular formula of C_12_H_20_O_3_ by HRESIMS data, indicating three degrees of unsaturation. Theirs ^1^H and ^13^C NMR data closely resembled those of **6**. The key difference was that the acetyl in **6** was reduced in **7** and **8**, which was supported by the comparison of chemical shift value of C-6 and unsaturation among **6**, **7** and **8**. The absolute configurations of **7** and **8** were referred from the comparison of experimental and calculational ECD. As a result, the absolute configurations of **7** and **8** were 5*R*,6*S* and 5*R*,6*R*, respectively (Fig. 3).

Additionally, for compounds **2–4** and **6**, both experimental and TD-DFT-calculated ECD spectra were obtained to assess their absolute configurations. Comparison of the calculated curves with the corresponding experimental ECD profiles revealed no distinct Cotton effects that could be attributed to a single enantiomer, suggesting that these compounds are likely present as racemic mixtures (Fig. 3). Attempts to achieve enantiomeric separation were unsuccessful due to the unavailability of a suitable chiral HPLC column. Accordingly, the stereochemical discussion for these compounds has been presented on this basis.

The structures of the known compounds (**9–15**) were determined by comparing their spectroscopic data with the literature values and identified as alternariol-3,9-dimethyl ether (**9**) [24], alternariol (**10**) [24], cladobotrin IV (**11**) [25], (*R*)-(–)-5-methylmellein (**12**) [26], alternariol 9-methylether (**13**) [24], 5-(1-hydroxyhexyl)-6-methyl-2*H*-pyran-2-one (**14**) [23], phomopthane B (**15**) [27].

**Fig. 4:**
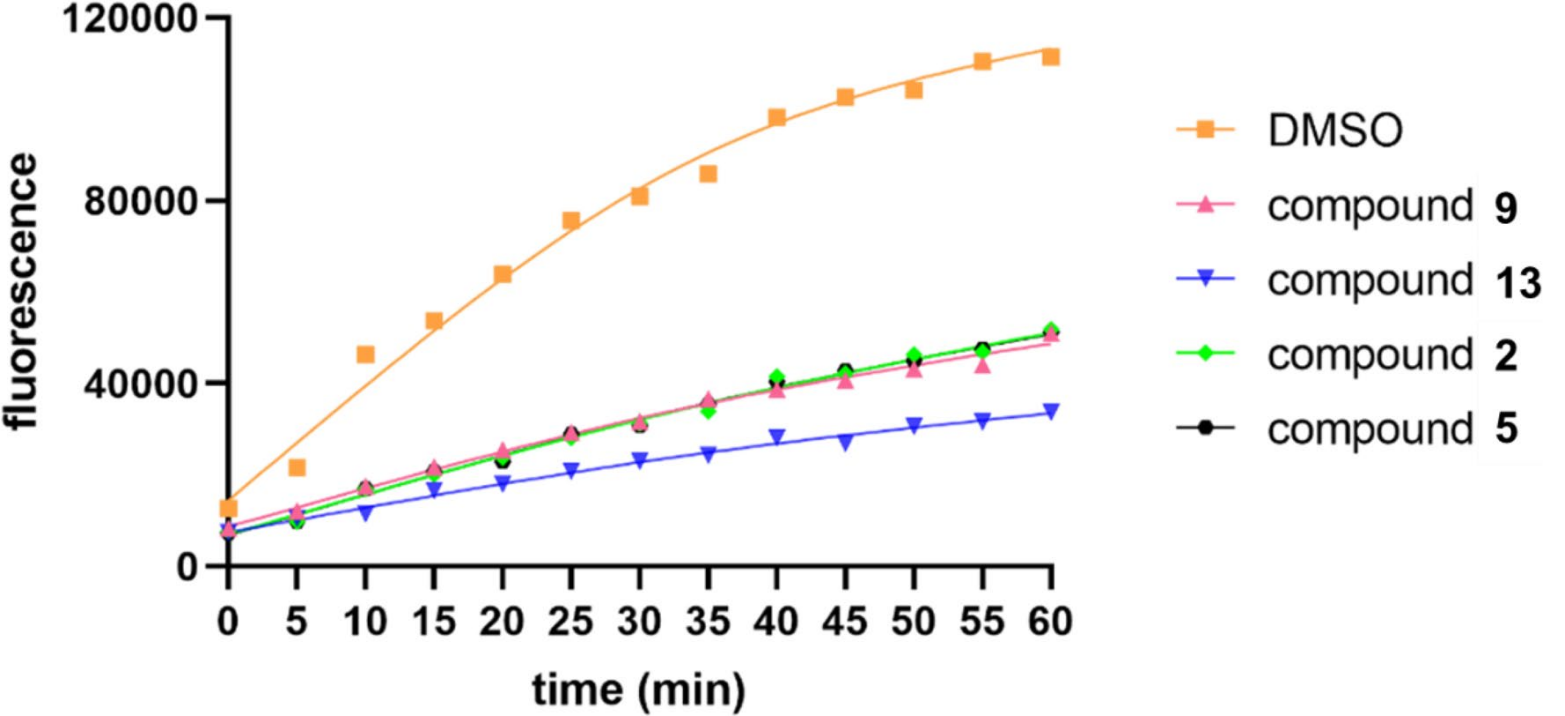
USP4 inhibition activity of **2**, **5**, **9**, **13** in 40 μM using the Ub-Rho110 as substrate

**Fig. 5:**
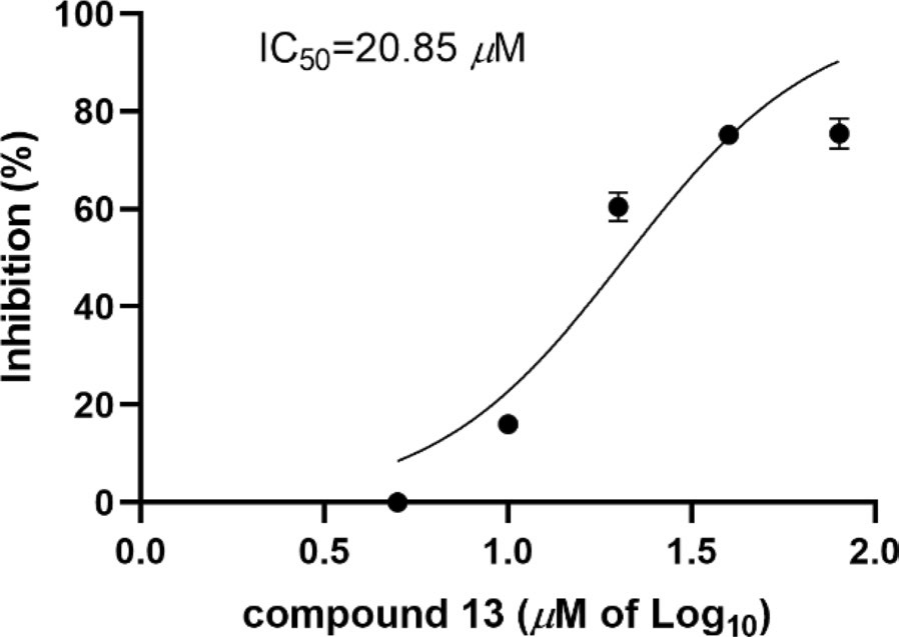
USP4 inhibition activity of **13**

Compounds **1–15** were screened for USP4 inhibition using Ubiquitin–Rhodamine 110 (Ub-Rho110) hydrolysis. Compounds **2, 5, 9,** and **13** exhibited moderate inhibition at 40 μM (Fig. 4). Compound **13** showed the most potent activity, with an IC_50_ value of 20.85 μM (Fig. 5). The remaining compounds displayed no significant inhibitory effects.

## Conclusions

In summary, we conducted a comprehensive study on the fungus *Diaporthe* sp. CCY4, from which 15 compounds were isolated from its fermentation products. The structures of these compounds were elucidated using spectroscopic techniques, including NMR, HRESIMS, and ECD. Bioactivity assays revealed that four compounds (**2, 5, 9,** and **13**) exhibited significant inhibitory effects on USP4 at a concentration of 40 μM. Notably, compound **13** demonstrated potent USP4 inhibition with an IC_50_ value of 20.85 μM, suggesting its potential as a candidate for therapeutic development. The discovery of these new metabolites not only expands the structural diversity of *α*-pyrone and *γ*-butenolide natural products but also underscores their promise as pharmaceutical lead compounds. Furthermore, this study highlights the value of plant endophytic fungi as a rich source of novel bioactive metabolites.

## Experimental

### General experimental procedures

Optical rotations were measured on a Rudolph Autopol V plus polarimeter, while NMR date were acquired on a Bruker Avance III HD 600 spectrometer with using tetramethylsilane (TMS) as an internal standard. HRESIMS data were determined on a Waters Acquity UPLC I-Class plus Xevo G2-XS Qtof mass spectrometer. Fluorescence date were measured on a SpectraMax i3x Multi-Mode Microplate Reader (Molecular Devices, Silicon Valley, California, USA). Semipreparative HPLC was performed on an Agilent 1100 liquid chromatograph with a Venusil MP-C_18_ (10 mm × 250 mm, 5 μm, 3 mL/min) column (Agilent Technologies Inc., California, USA). Lichroprep RP-18 gel (40–63 μm, Merck, Darmstadt, Germany), Column chromatography (CC) was performed with silica gel (200–300 mesh; Qingdao Marine Chemical, Inc., Qingdao, China).

### Fungal material and fermentation

The endophytic fungal strain *Diaporthe* sp. CCY4 was isolated from the plant of *Camellia japonica*, which was collected at the city of Kunming, China (102°51′14″E, 24°50′30″N), in August 2021. The plant was identified by Prof. Yong Xiong, Yunnan Minzu University, Kunming, China. The fungal strain was identified as *Diaporthe* sp. according to its ITS region sequence (NCBI accession number PX112654). The strain is preserved in the Key Laboratory of Chemistry in Ethnic Medicinal Resources of Ministry of Education, Yunnan Minzu University.

The *Diaporthe* sp. CCY4 strain was cultured on potato dextrose agar (PDA) slants at 30 °C for 3 days. Then agar plugs which covered with hyphae were cut into small pieces of about 1 × 1 × 1 mm^3^, and small amount of agar plugs were inoculated in five sterile Erlenmeyer flasks (500 mL), each containing 200 mL of potato dextrose broth. These five flasks of the inoculated media were incubated at 30 °C on a rotary shaker at 200 rpm for 3 days to prepare the seed culture. Ten liters of potato dextrose broth were distributed in 50 sterile Erlenmeyer flasks (500 mL), each containing 200 mL, sterilized by autoclave. Each flask was inoculated with 10 mL of the seed inoculum and incubated at 30 °C for 12 days.

### Extraction and isolation

Ten liters of fermentation material were separated into mycelium and fermentation liquor by gauze.

The mycelium was soaked in acetone and ultrasonicated for three times, and the obtained solution was evaporated in vacuum until acetone was absent. Then the aqueous phase was extracted with ethyl acetate (1:1, *v/v*) and the ethyl acetate was evaporated to dryness under vacuum to afford a crude extract (2.66 g). The crude extract of mycelium was subjected to silica gel CC (100–200 mesh), eluted with CH_2_Cl_2_-MeOH (1:0–5:1 gradient system), to give four fractions (Fr.A-D). Fr.B (535.2 mg) was subjected to silica gel CC (200–300 mesh), and eluted with petroleum ether-EtOAc (30:1, 20:1, 10:1 and 5:1) to afford three fractions (Fr.B1-B4). Fr.B2 (26 mg) was separated by semipreparative HPLC (eluted with CH_3_CN/H_2_O, 65:35, 3 mL/min) to obtain compound **12** (2.0 mg,* t*_R_ = 19.3 min). Fr.B4 (32 mg) was separated by semipreparative HPLC (Venusil MP-C_18_ column, eluted with CH_3_CN/H_2_O, 85:15, 3 mL/min, λ = 210 nm) to obtain compound **9** (3.8 mg,* t*_R_ = 25.3 min). Fr.C (2.0 g) was subjected to silica gel CC (200–300 mesh), and eluted with petroleum ether-EtOAc (20:1, 10:1, 5:1 and 2:1) to afford three fractions (Fr.C1-C4). Fr.C2 (217 mg) was separated by semipreparative HPLC (Venusil MP-C_18_ column, eluted with 75 − 80% CH_3_CN/H_2_O, 3 mL/min, λ = 210 nm) to obtain compound **1** (18.9 mg,* t*_R_ = 24 min), **2** (4.0 mg,* t*_R_ = 23.5 min), and **8** (12.9 mg,* t*_R_ = 22.5 min). Fr.C3 (43 mg) was separated by semipreparative HPLC (Venusil MP-C_18_ column, eluted with CH_3_CN/H_2_O, 70:30, 3 mL/min, λ = 210 nm) to obtain compound **13** (2.5 mg,* t*_R_ = 20.0 min). Fr.C4 (247.5 mg) was purified by RP-C18 gel CC with MeOH-H_2_O (30:70, 45:55, 50:50 and 60:40) to yield 4 fractions. Subsequently, the 45% MeOH sample (57 mg) was purified by semipreparative HPLC (Venusil MP-C_18_ column, eluted with CH_3_CN/H_2_O, 43:57, 3 mL/min, λ = 210 nm) to yield **11** (17.7 mg, *t*_R_ = 11.2 min). In addition, a 50% MeOH sample (28 mg) was purified by semipreparative HPLC (Venusil MP-C_18_ column, eluted with 50 − 55% CH_3_CN/H_2_O, 3 mL/min, λ = 210 nm) to give **10** (2.8 mg, *t*_R_ = 15.2 min).

The fermented liquor was extracted with EtOAc (4 × 10.0 L), and the organic solvent was evaporated to dryness under vacuum to afford a crude extract (3.0 g). The extract was subjected to silica gel CC (100–200 mesh), eluted with petroleum ether-ethyl acetate (1:0–5:1 gradient system), to give five fractions (Fr.E-I). Fr.G (1.6 g) was purified by RP-C18 gel CC with MeOH-H_2_O (30:70, 40:60, 45:55, 50:50, 55:45 and 65:35) to yield Fr.G1-G6. Subsequently, Fr.G3 was purified by semipreparative HPLC (Venusil MP-C_18_ column, 40 − 45% CH_3_CN/H_2_O, 3 mL/min, λ = 210 nm) to yield **4** (12.3 mg, *t*_R_ = 11.5 min) and **15** (4.3 mg, *t*_R_ = 12.5 min). Fr.G5 (200 mg) was purified by semipreparative HPLC (Venusil MP-C_18_ column, 50 − 55% CH_3_CN/H_2_O, 3 mL/min, λ = 210 nm) to yield **3** (15.6 mg, *t*_R_ = 14.2 min) and **14** (26.8 mg, *t*_R_ = 15.5 min). Fr.G6 (326 mg) was purified by semipreparative HPLC (Venusil MP-C_18_ column, 50 − 55% CH_3_CN/H_2_O, 3 mL/min, λ = 210 nm) to yield **5** (47.5 mg, *t*_R_ = 16.2 min), **6** (1.8 mg, *t*_R_ = 16.8 min), and **7** (2.8 mg, *t*_R_ = 17.0 min), respectively.

Diaporpyrone G (**1**). Yellow oily substance; UV (CH_3_CN) *λ*_max_ (log *ɛ*) 200 (3.03), 220 (2.71), 308 (3.00) nm; for ^1^H and ^13^C NMR data, see Table; HRESIMS *m/z* 195.1377 [M + H]^+^ (calcd for C_12_H_19_O_2_, 195.1379).

Diaporpyrone H (**2**). Colorless oil; $[\alpha]_{\rm{D}}^{22}$0 (*c* 0.045, MeOH); UV (CH_3_CN) *λ*_max_ (log *ɛ*) 195 (3.63) nm; for ^1^H and ^13^C NMR data, see Tables 1 and 2; HRESIMS *m/z* 211.1329 [M + H]^+^ (calcd for C_12_H_19_O_3_, 211.1328).

Diaporpyrone I (**3**). Yellow oily substance; $[\alpha]_{\rm{D}}^{22}$–10.3 (*c* 0.058, MeOH); UV (CH_3_CN) *λ*_max_ (log *ɛ*) 198 (3.70), 308 (3.67) nm; for ^1^H and ^13^C NMR data, see Tables 1 and 2; HRESIMS *m/z* 211.1331 [M + H]^+^ (calcd for C_12_H_19_O_3_, 211.1328).

Diaporpyrone J (**4**). Yellow oily substance; $[\alpha]_{\rm{D}}^{22}$ –7.9 (*c* 0.063, MeOH); UV (CH_3_CN) *λ*_max_ (log *ɛ*) 198 (3.80), 220 (3.22), 310 (3.56) nm; for ^1^H and ^13^C NMR data, see Tables 1 and 2; HRESIMS *m/z* 211.1328 [M + H]^+^ (calcd for C_12_H_19_O_3_, 211.1328).

Diaporpyrone K (**5**). Yellow oily substance; UV (CH_3_CN) *λ*_max_ (log *ɛ*) 198 (3.34), 304 (3.22) nm; for ^1^H and ^13^C NMR data, see Tables 1 and 2; HRESIMS *m/z* 211.1326 [M + H]^+^ (calcd for C_12_H_19_O_3_, 211.1328).

Porbutenolide A (**6**). Yellow oily substance; $[\alpha]_{\rm{D}}^{25}$ + 0.8 (*c* 0.25, MeOH); UV (CH_3_CN) *λ*_max_ (log *ɛ*) 196 (2.97), 224 (2.91), 288 (1.67) nm; for ^1^H and ^13^C NMR data, see Table 3; HRESIMS *m/z* 233.1145 [M + Na]^+^ (calcd for C_12_H_18_O_3_Na, 233.1148).

Porbutenolide B (**7**). Colorless oil;$[\alpha]_{\rm{D}}^{25}$+ 3.0 (*c* 0.033, MeOH); UV (CH_3_CN) *λ*_max_ (log *ɛ*) 208 (3.83) nm; ECD (MeOH) λ_max_ (Δε): 219 (1.80) nm. for ^1^H and ^13^C NMR data, see Table 3; HRESIMS *m/z* 213.1531 [M + H]^+^ (calcd for C_12_H_21_O_3_, 213.1485).

Porbutenolide C (**8**). Colorless oil; $[\alpha]_{\rm{D}}^{22}$ –10.3 (*c* 0.047, MeOH); UV (CH_3_CN) *λ*_max_ (log *ɛ*) 205 (3.90) nm; ECD (MeOH) λ_max_ (Δε): 220 (–2.52) nm.for ^1^H and ^13^C NMR data, see Table 3; HRESIMS *m/z* 213.1479 [M + H]^+^ (calcd for C_12_H_21_O_3_, 213.1485).

### Quantum chemical calculation method

Compound conformations were energy-minimized (ChemDraw 3D) and subjected to a systematic search (Sybyl 2.0), retaining conformers within 6 kcal/mol of the global minimum. Conformers underwent geometry optimization at the B3LYP/6-31G(d) level in methanol (CPCM solvation model) using Gaussian. Electronic Circular Dichroism (ECD) spectra were then calculated at the B3LYP/6–31 + G(d) level. Theoretical spectra were generated in SpecDis by Boltzmann-averaging individual conformer spectra according to their relative energies. The calculated ECD spectrum was compared to the experimental data to assign the absolute configuration.

### USP4 inhibitiory assay

Ub-Rho110 (Boston Biochem) hydrolysis by USP4 was monitored fluorometrically. Compounds were dissolved in DMSO. FLAG-tagged USP4 (30 nM) was pre-incubated with compounds or DMSO in assay buffer (20 mM Tris–HCl, pH 8.0, 2 mM CaCl₂, 2 mM *β*-mercaptoethanol). Reactions were initiated by adding Ub-Rho110 substrate (300 nM) to the mixture in a black 96-well plate. After incubation at 37 °C for 30 min, fluorescence was measured at 5-min intervals (Ex 485 nm/Em 535 nm) using a SpectraMax i3x microplate reader.

## Supplementary Information

Additional file 1.

## Data Availability

All data generated or analyzed during this study are included in this published article and its supplementary information files.
