# Supplementary material for: Eight new α-pyrone and γ-butenolide derivatives from the plant endophytic fungus Diaporthe sp. CCY4
Source: Nat Prod Bioprospect. 2026 Feb 3;16(1):29. doi: 10.1007/s13659-025-00580-1 (PMC12864556; doi:10.1007/s13659-025-00580-1)
Supplement: Supplementary file 1 — Additional file 1. [file 13659_2025_580_MOESM1_ESM.docx]

Supporting Information for

**Eight new α-pyrone and *γ*-butenolide derivatives from the plant endophytic fungus *Diaporthe* sp. CCY4**

Jie-Chun Zeng, Xu-Ping Zhang, Lu Gao, Qian-Qian Yin*, Wei-Guang Wang*

Key Laboratory of Chemistry in Ethnic Medicinal Resources of Ministry of Education, Yunnan Minzu University, Kunming 650031, Yunnan, PR China

* Corresponding authors.

Email: wwg@live.cn (Wei-Guang Wang); [yinqianpharm@163.com](mailto:yinqianpharm@163.com) (Qian-Qian Yin)

**Contents**

**Figure S1.** ^1^H NMR spectrum of **1** 1

**Figure S2.** ^13^C NMR spectrum of **1** 1

**Figure S3.** COSY spectrum of **1** 2

**Figure S4.** HSQC spectrum of **1** 2

**Figure S5.** HMBCspectrum of **1** 3

**Figure S6.** HRESIMS spectrum of **1** 4

**Figure S7.** UV spectrum of **1** 4

**Figure S8.** ^1^H NMR spectrum of **2** 5

**Figure S9.** ^13^C NMR spectrum of **2** 5

**Figure S10.** COSY spectrum of **2** 6

**Figure S11.** HSQC spectrum of **2** 6

**Figure S12.** HMBC spectrum of **2** 7

**Figure S13.** HRESIMS spectrum of **2** 8

**Figure S14.** UV spectrum of **2** 8

**Figure S15.** ^1^H NMR spectrum of **3** 9

**Figure S16.** ^13^C NMR spectrum of **3** 9

**Figure S17.** COSY spectrum of **3** 10

**Figure S18.** HSQC spectrum of **3** 10

**Figure S19.** HMBC spectrum of **3** 11

**Figure S20.** HRESIMS spectrum of **3** 12

**Figure S21.** UV spectrum of **3** 12

**Figure S22.** ^1^H NMR spectrum of **4** 13

**Figure S23.** ^13^C NMR spectrum of **4** 13

**Figure S24.** COSY spectrum of **4** 14

**Figure S25.** HSQC spectrum of **4** 14

**Figure S26.** HMBC spectrum of **4** 15

**Figure S27.** HRESIMS spectrum of **4** 16

**Figure S28.** UV spectrum of **4** 16

**Figure S29.** ^1^H NMR spectrum of **5** 17

**Figure S30.** ^13^C NMR spectrum of **5** 17

**Figure S31.** COSY spectrum of **5** 18

**Figure S32.** HSQC spectrum of **5** 18

**Figure S33.** HMBC spectrum of **5** 19

**Figure S34.** HRESIMS spectrum of **5** 20

**Figure S35.** UV spectrum of **5** 20

**Figure S36.** ^1^H NMR spectrum of **6** 21

**Figure S37.** ^13^C NMR spectrum of **6** 21

**Figure S38.** COSY spectrum of **6** 22

**Figure S39.** HSQC spectrum of **6** 22

**Figure S40.** HMBC spectrum of **6** 23

**Figure S41.** HRESIMS spectrum of **6** 24

**Figure S42.** UV spectrum of **6** 24

**Figure S43.** ^1^H NMR spectrum of **7** 25

**Figure S44.** ^13^C NMR spectrum of **7** 25

**Figure S45.** COSY spectrum of **7** 26

**Figure S46.** HSQC spectrum of **7** 26

**Figure S47.** HMBC spectrum of **7** 27

**Figure S48.** HRESIMS spectrum of **7** 28

**Figure S49.** UV spectrum of **7** 28

**Figure S50.** ^1^H NMR spectrum of **8** 29

**Figure S51.** ^13^C NMR spectrum of **8** 29

**Figure S52.** COSY spectrum of **8** 30

**Figure S53.** HSQC spectrum of **8** 30

**Figure S54.** HMBC spectrum of **8** 31

**Figure S55.** HRESIMS spectrum of **8** 32

**Figure S56.** UV spectrum of **8** 32

**Table S1**. Important thermodynamic parameters and Boltzmann distributions of the optimized **2** at B3LYP/6-31G(d) level in gas phase. 33

**Table S2**. The coordinates for the lowest energy conformers of **2** 33

**Table S3**. Important thermodynamic parameters and Boltzmann distributions of the optimized **3** at B3LYP/6-31G(d) level in gas phase. 37

**Table S4**. The coordinates for the lowest energy conformers of **3** 37

**Table S5**. Important thermodynamic parameters and Boltzmann distributions of the optimized **4** at B3LYP/6-31G(d) level in gas phase. 43

**Table S6**. The coordinates for the lowest energy conformers of **4** 43

**Table S7**. Important thermodynamic parameters and Boltzmann distributions of the optimized **6** at B3LYP/6-31G(d) level in gas phase. 45

**Table S8**. The coordinates for the lowest energy conformers of **6** 46

**Table S9**. Important thermodynamic parameters and Boltzmann distributions of the optimized **7** at B3LYP/6-31G(d) level in gas phase. 49

**Table S10**. The coordinates for the lowest energy conformers of **7**. 49

**Table S11**. Important thermodynamic parameters and Boltzmann distributions of the optimized **8** at B3LYP/6-31G(d) level in gas phase. 53

**Table S12**. The coordinates for the lowest energy conformers of **8**. 53


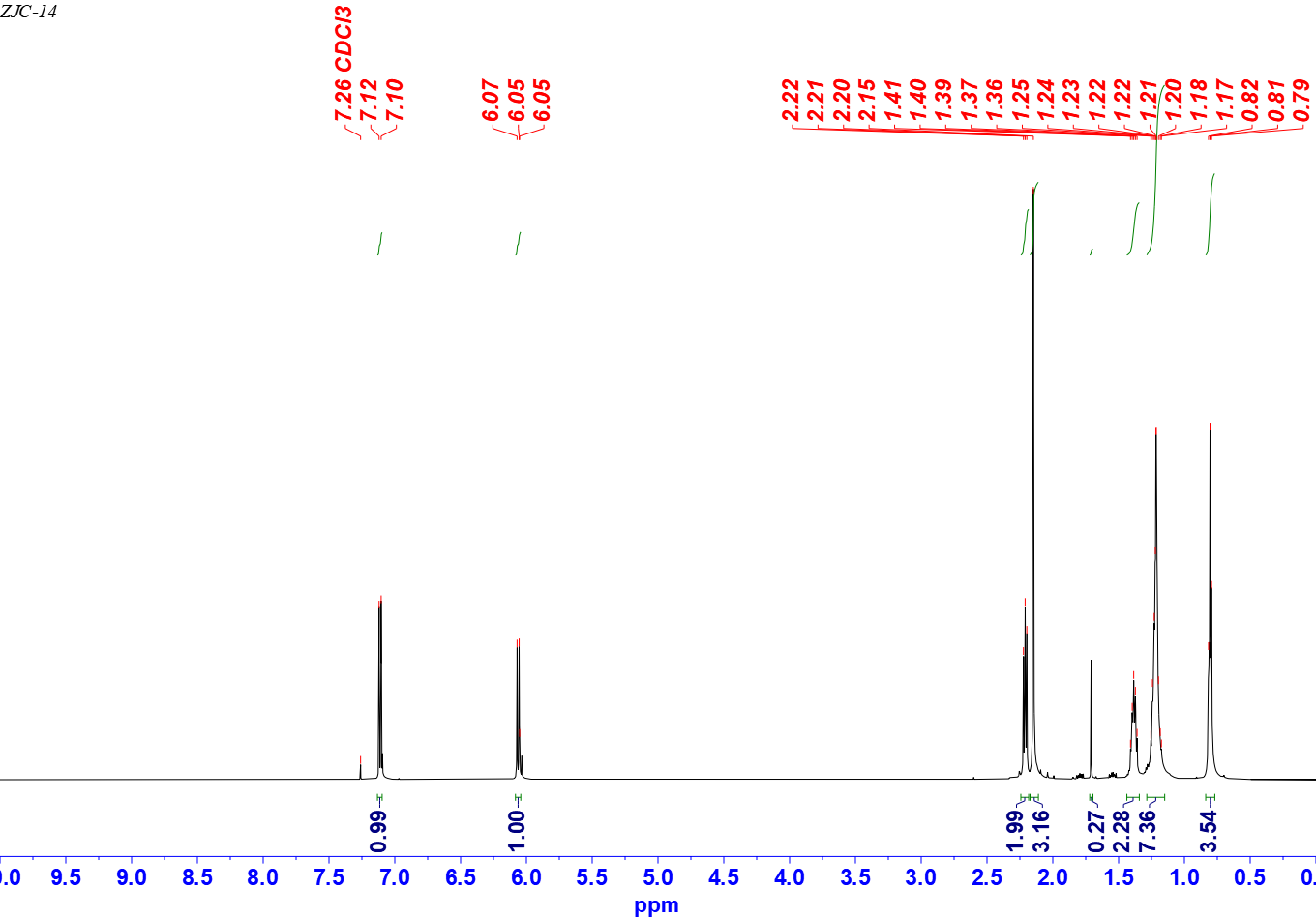


**Figure S1.** ^1^H NMR spectrum of **1**


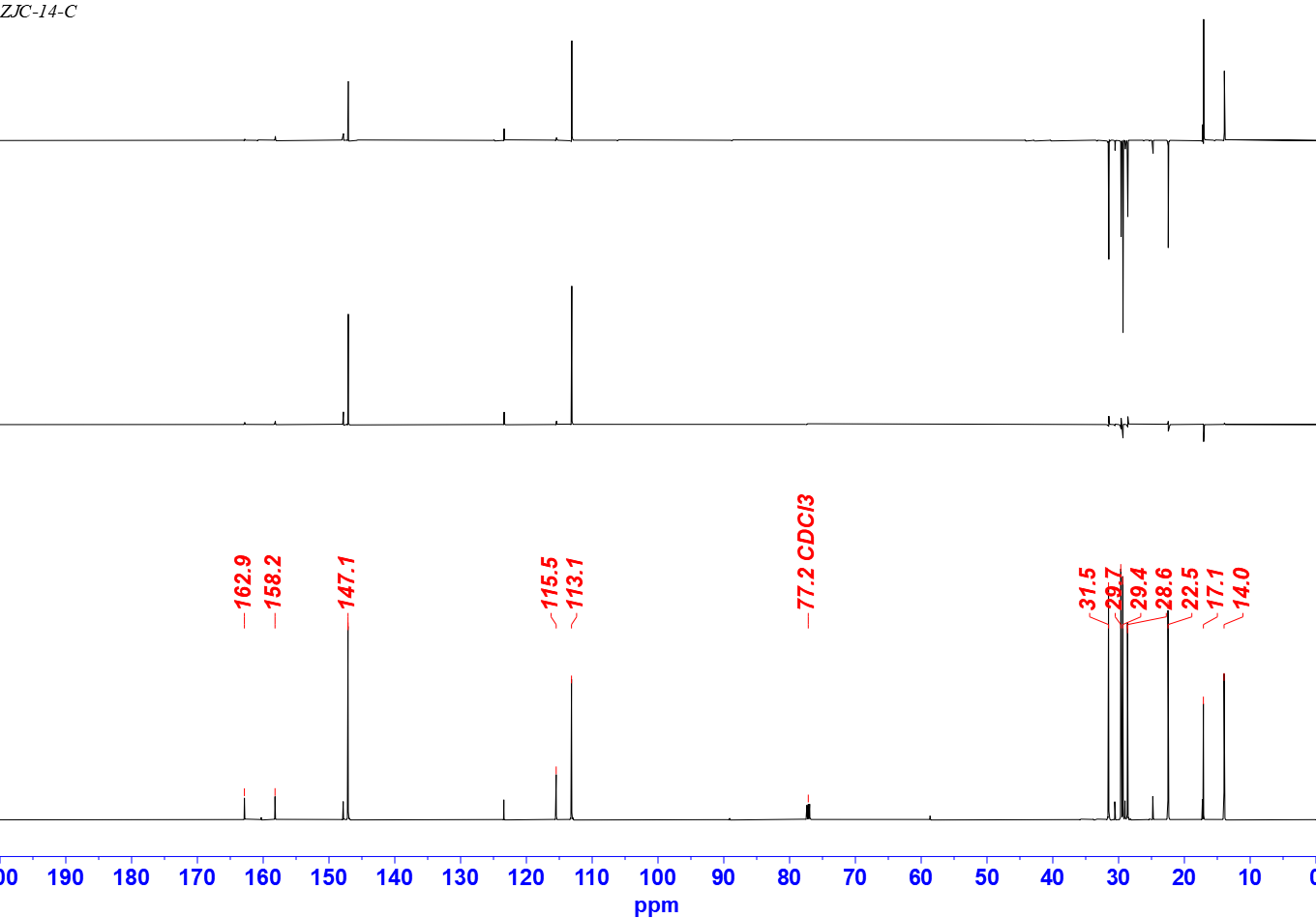


**Figure S2.** ^13^C NMR spectrum of **1**


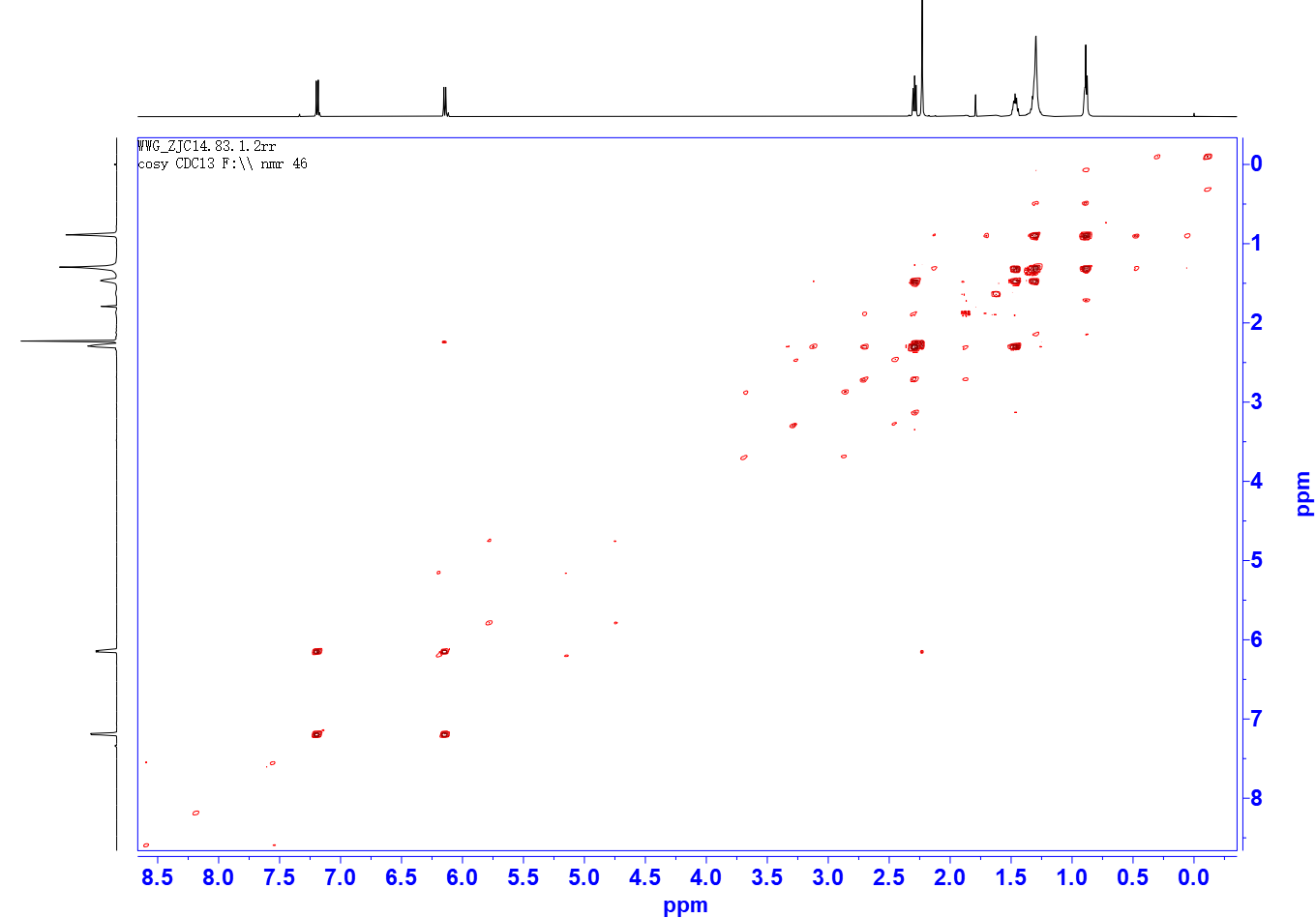


**Figure S3.** COSY spectrum of **1**


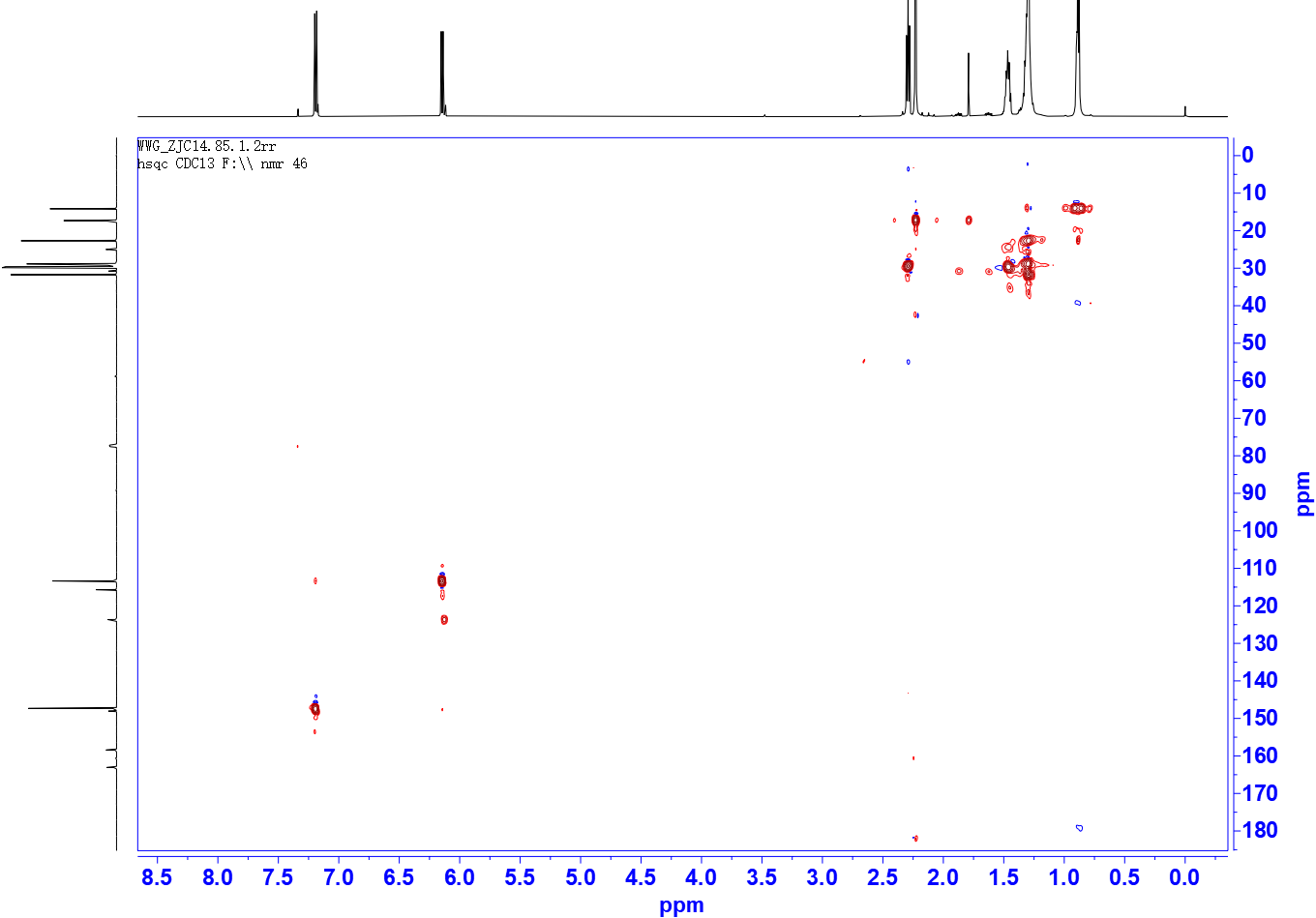


**Figure S4.** HSQC spectrum of **1**


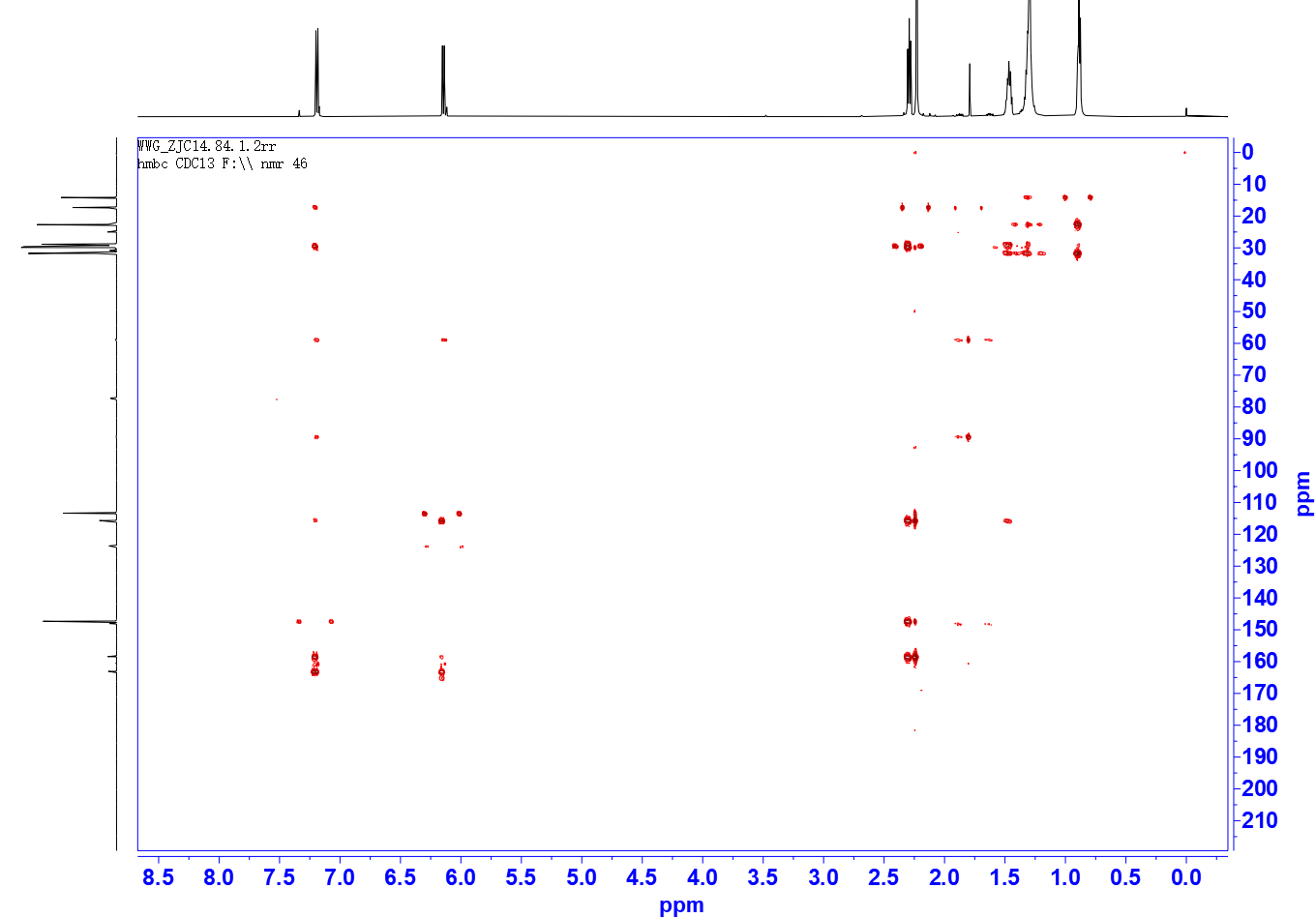


**Figure S5.** HMBCspectrum of **1**


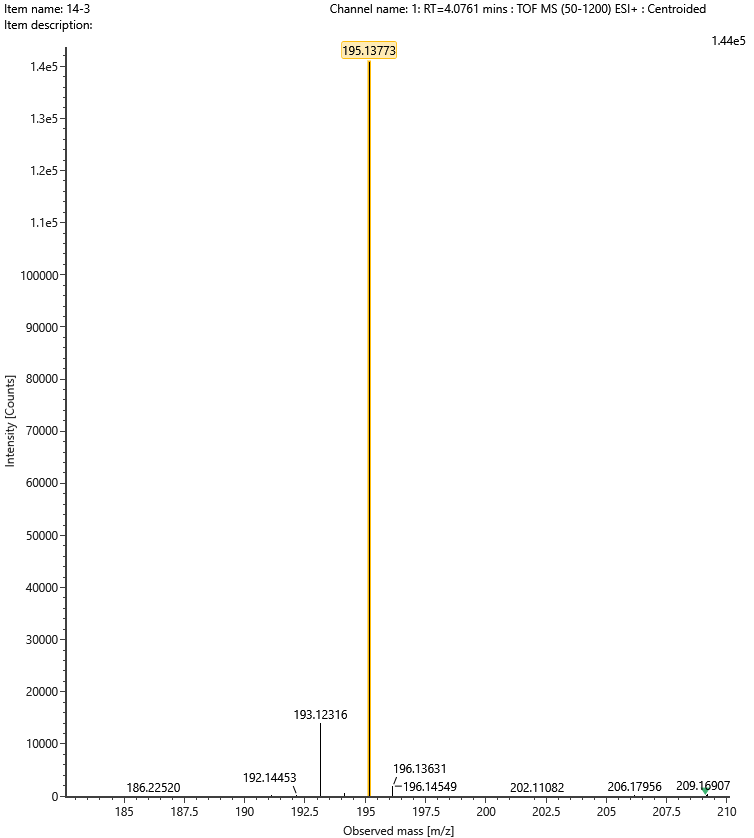


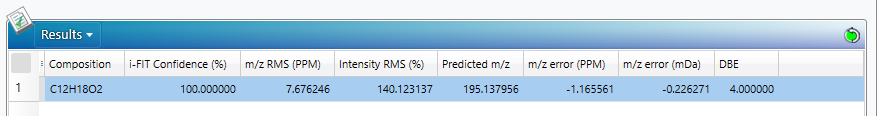


**Figure S6.** HRESIMS spectrum of **1**

**Figure S7.** UV spectrum of **1**


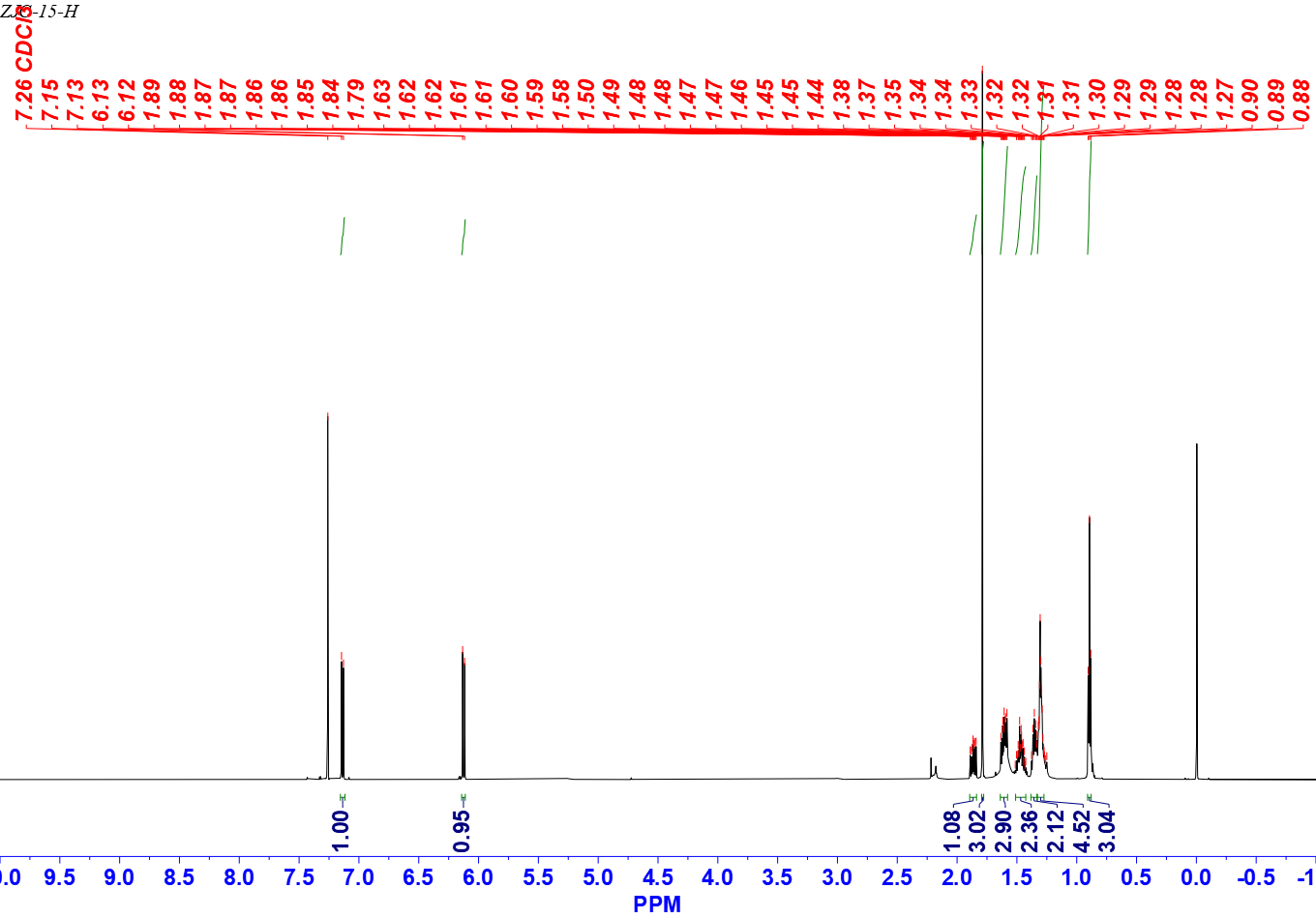


**Figure S8.** ^1^H NMR spectrum of **2**


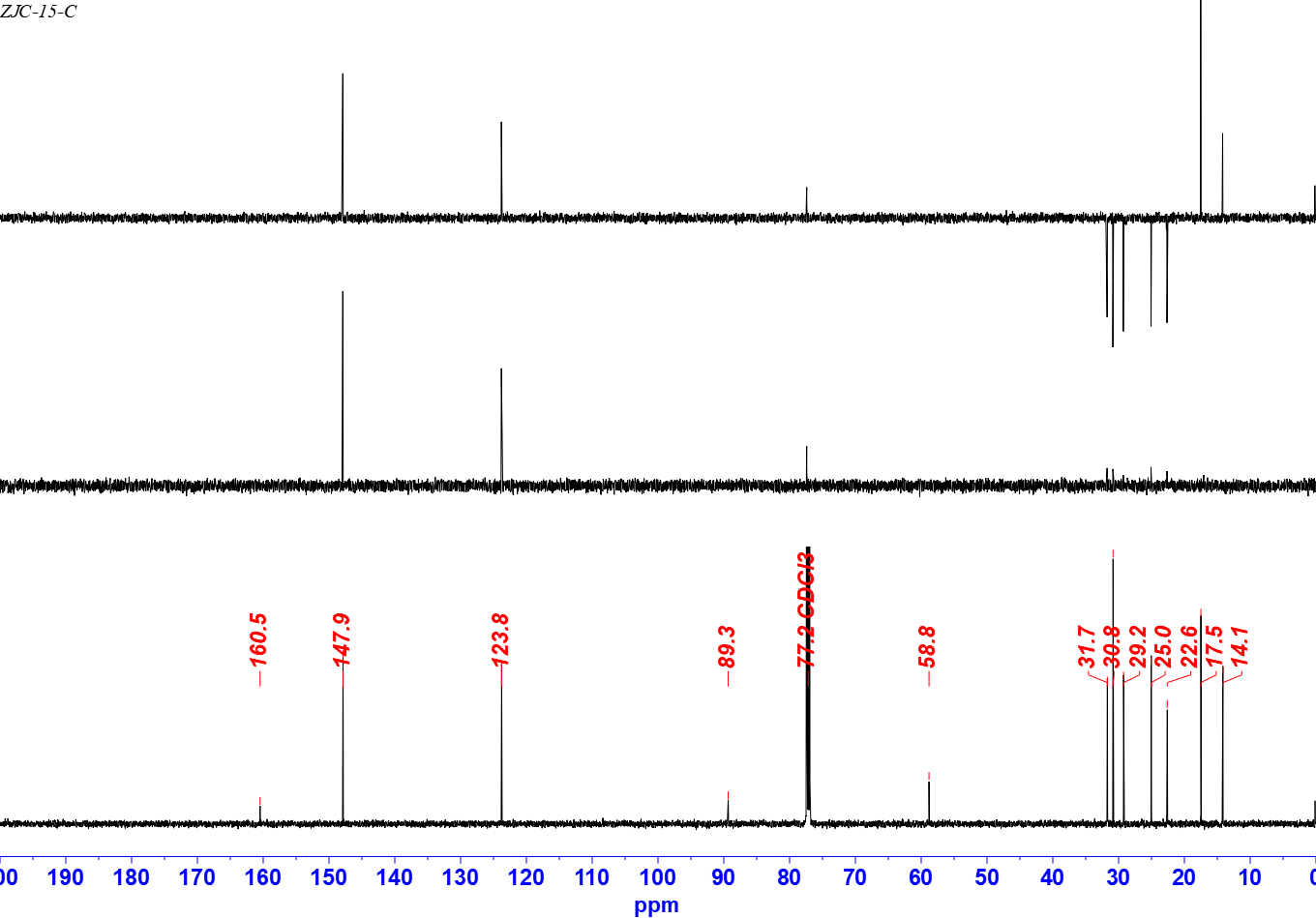


**Figure S9.** ^13^C NMR spectrum of **2**


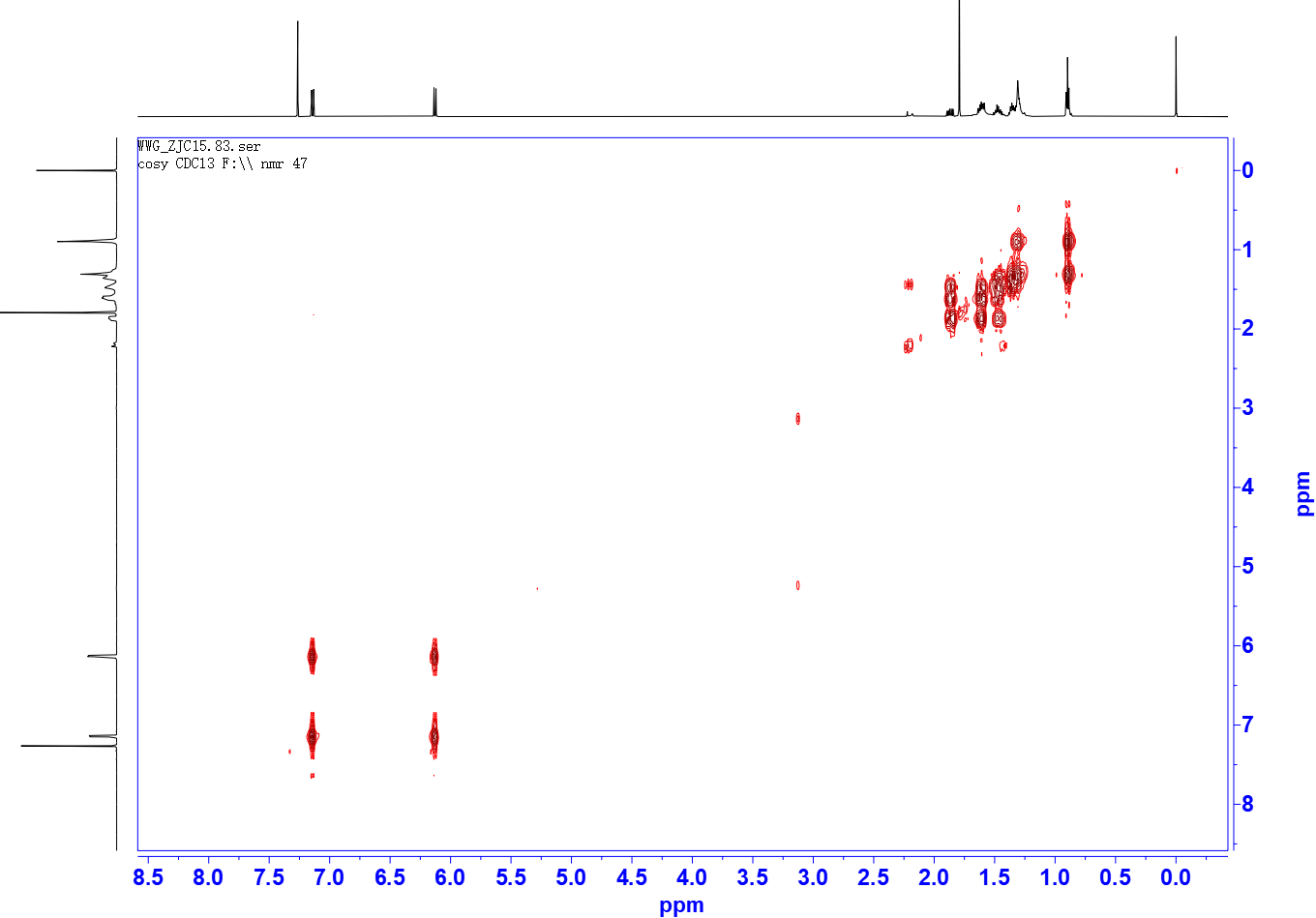


**Figure S10.** COSY spectrum of **2**


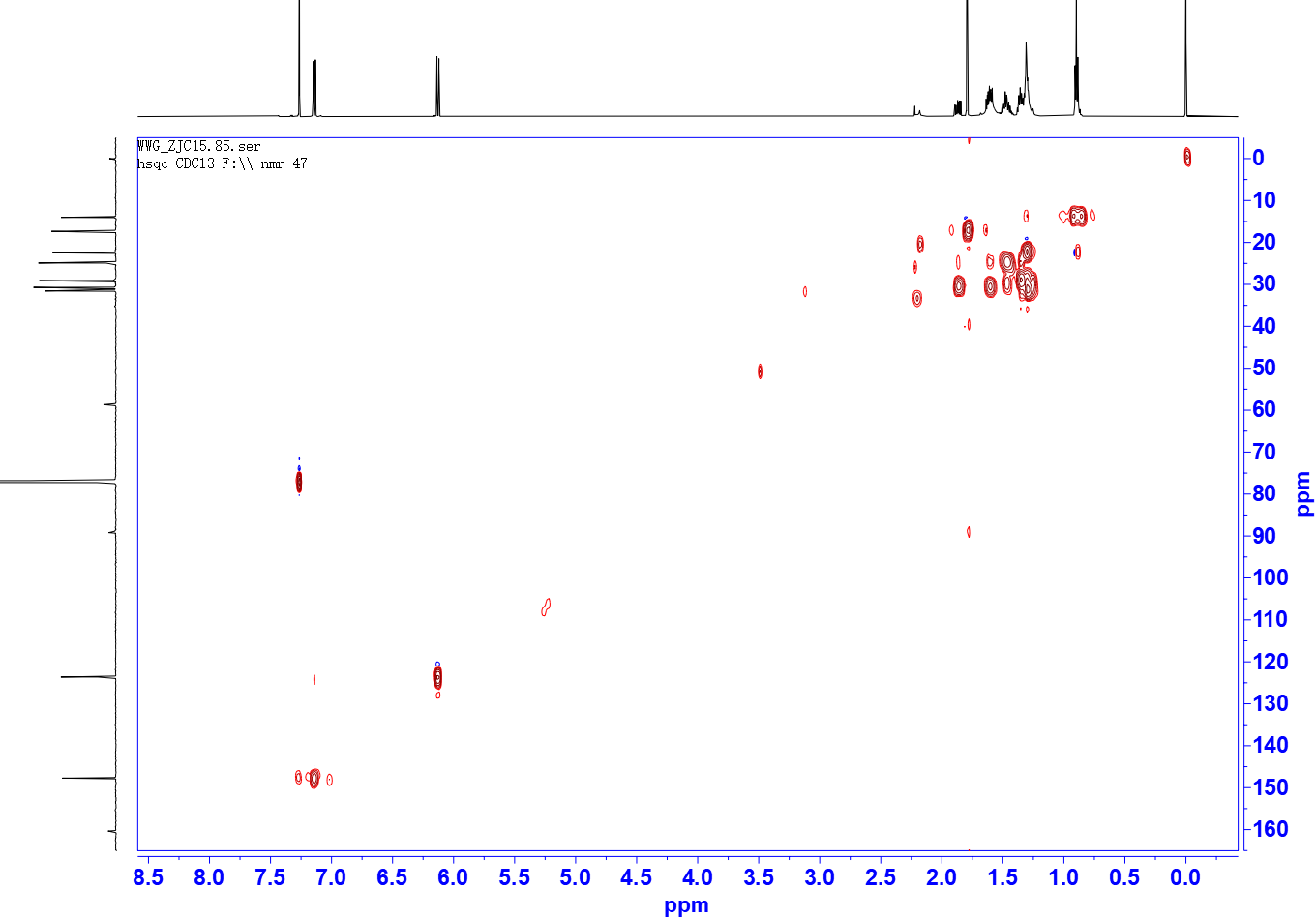


**Figure S11.** HSQC spectrum of **2**


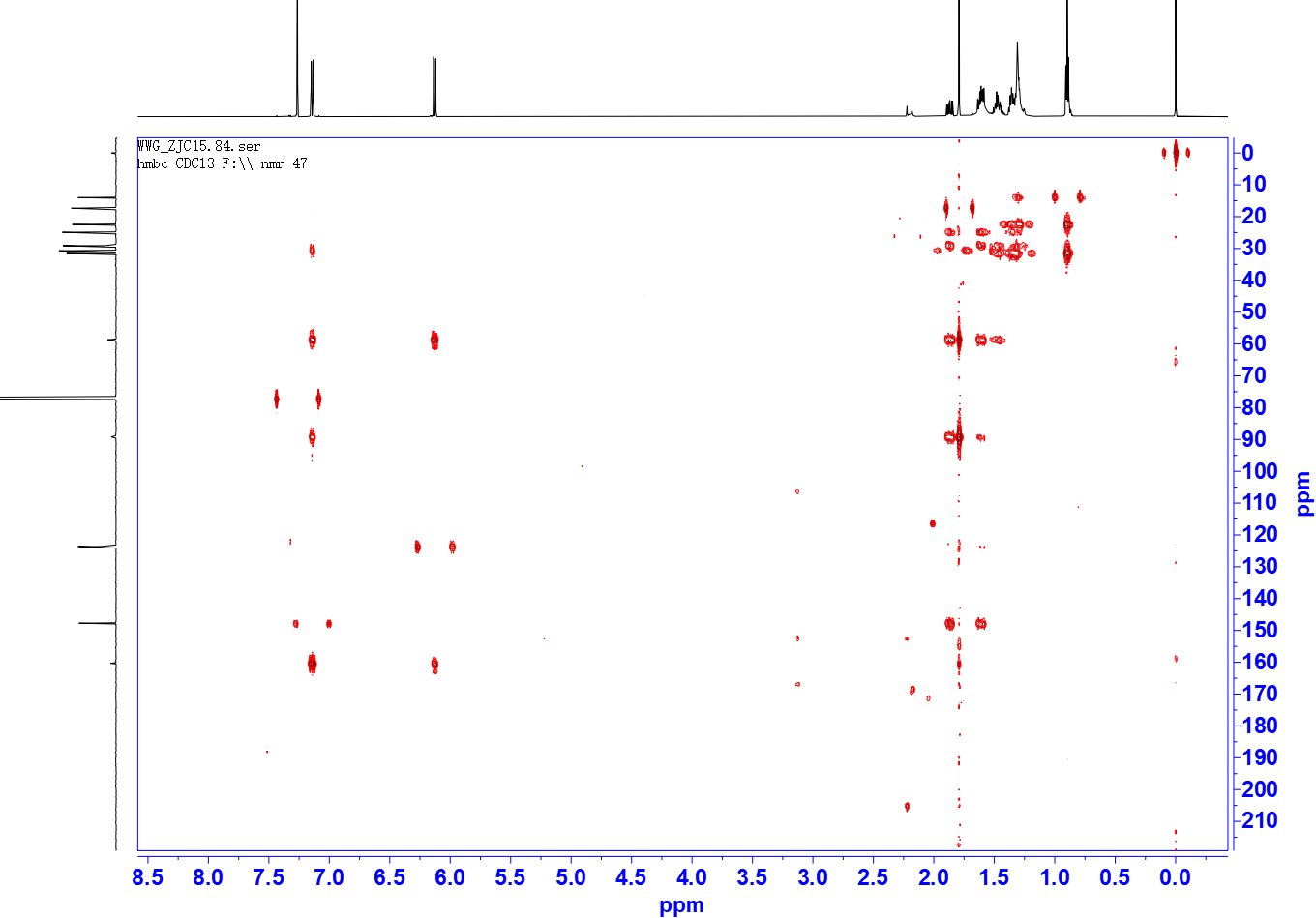


**Figure S12.** HMBC spectrum of **2**


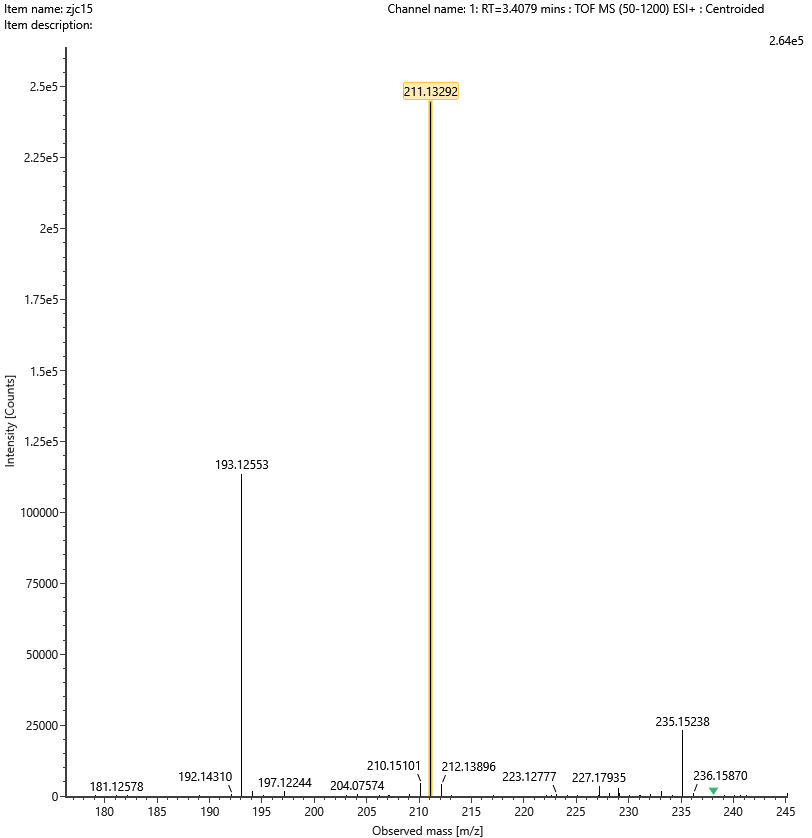


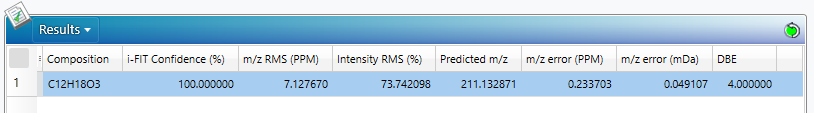


**Figure S13.** HRESIMS spectrum of **2**

**Figure S14.** UV spectrum of **2**


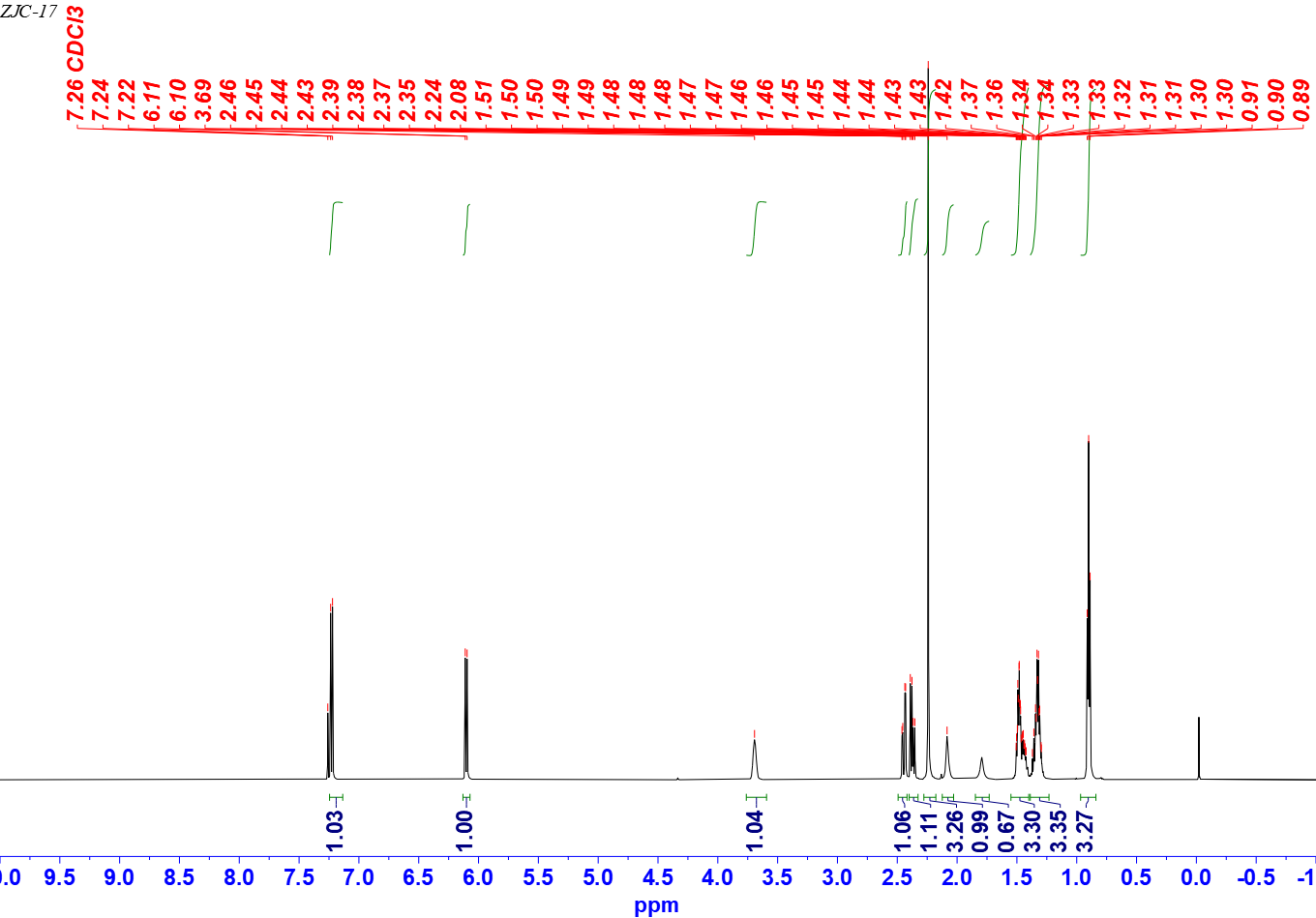


**Figure S15.** ^1^H NMR spectrum of **3**


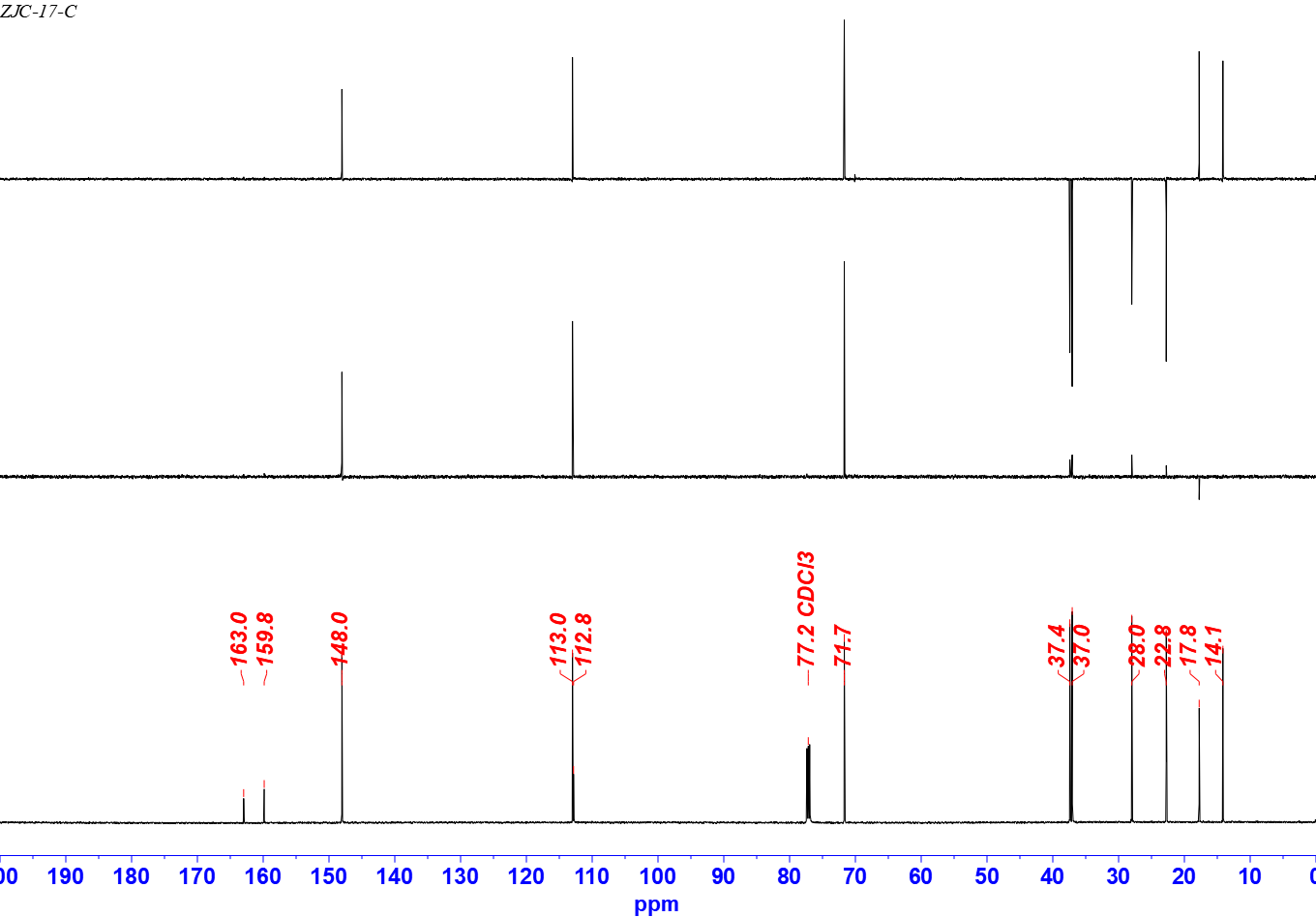


**Figure S16.** ^13^C NMR spectrum of **3**


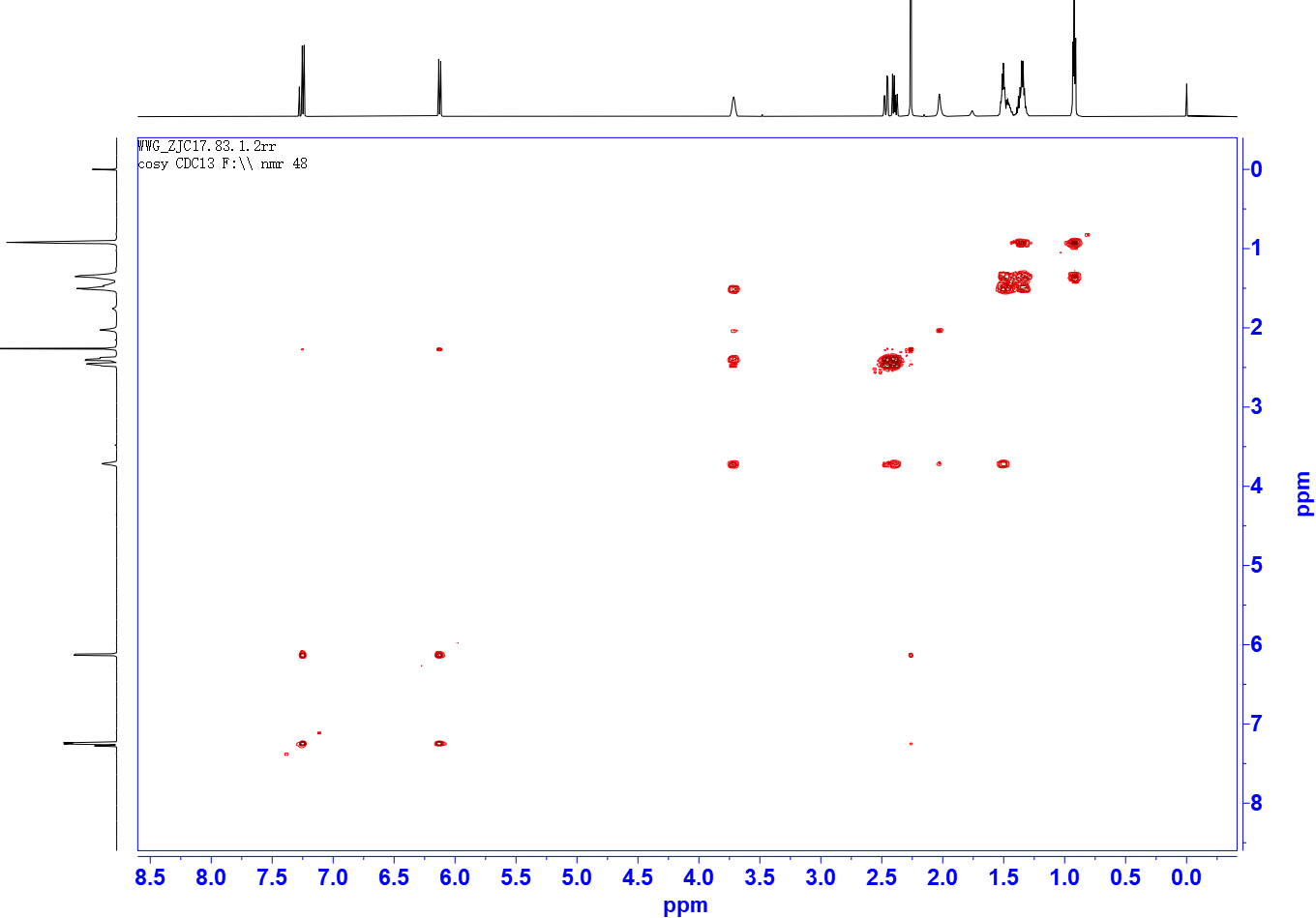


**Figure S17.** COSY spectrum of **3**


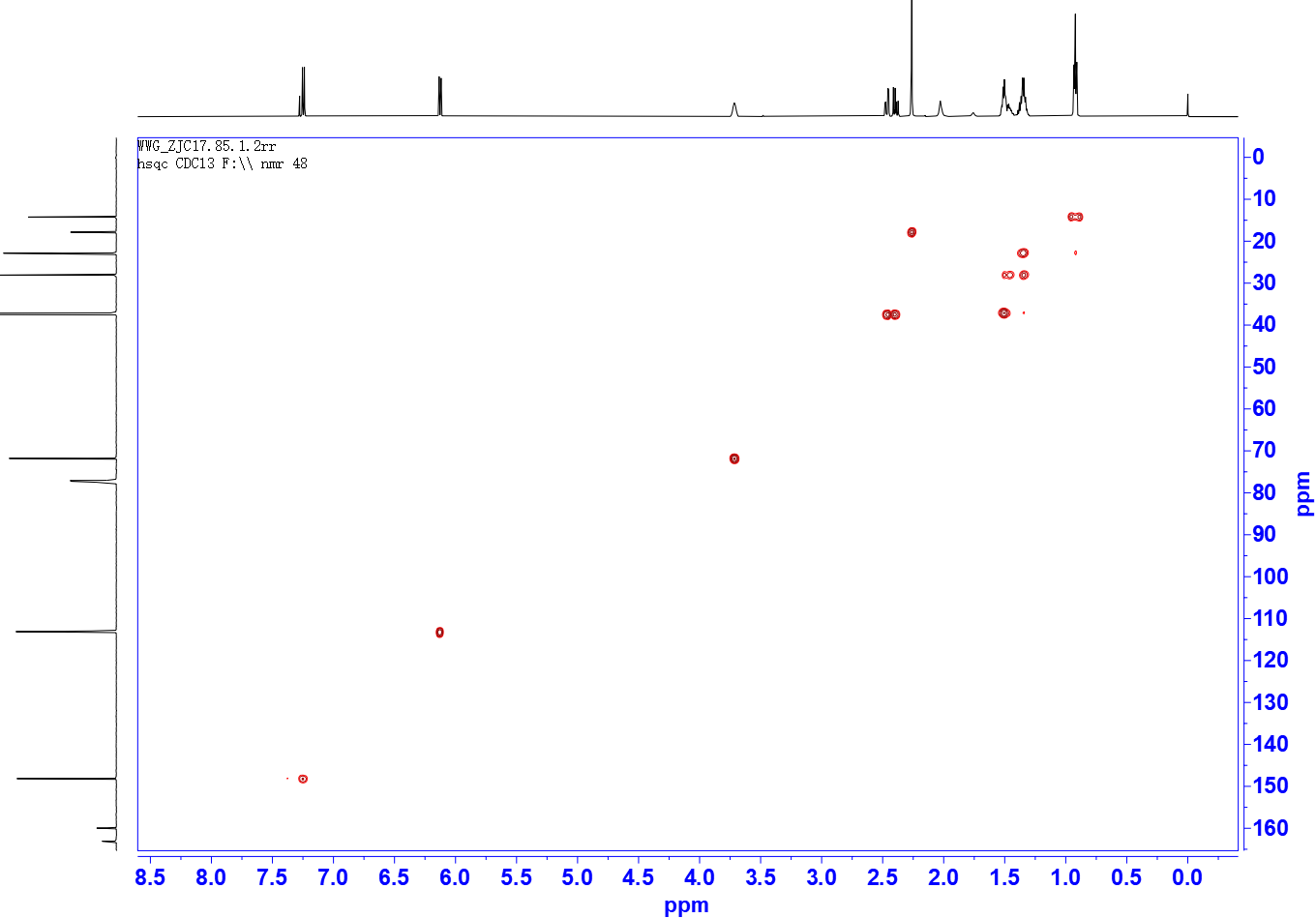


**Figure S18.** HSQC spectrum of **3**


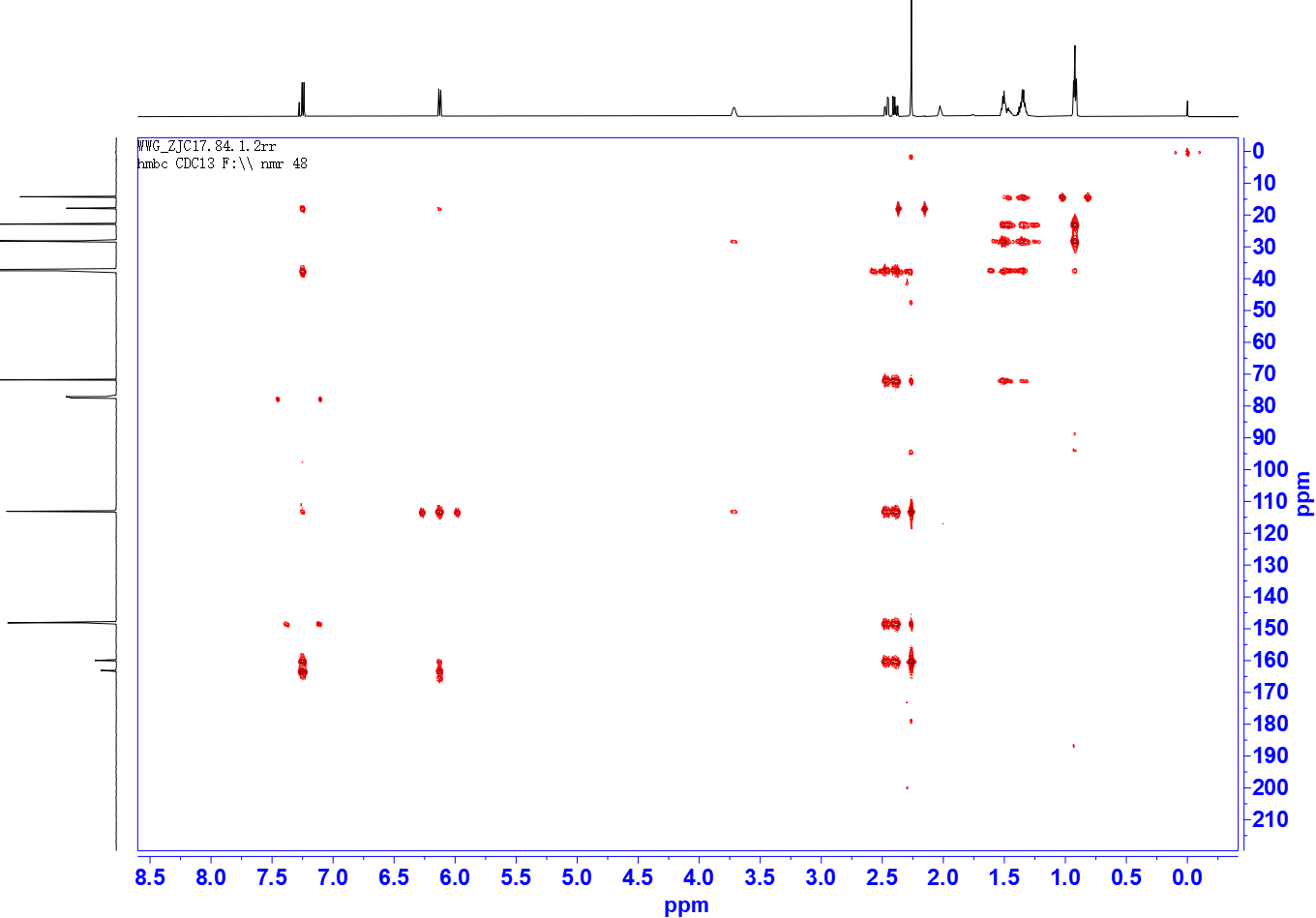


**Figure S19.** HMBC spectrum of **3**


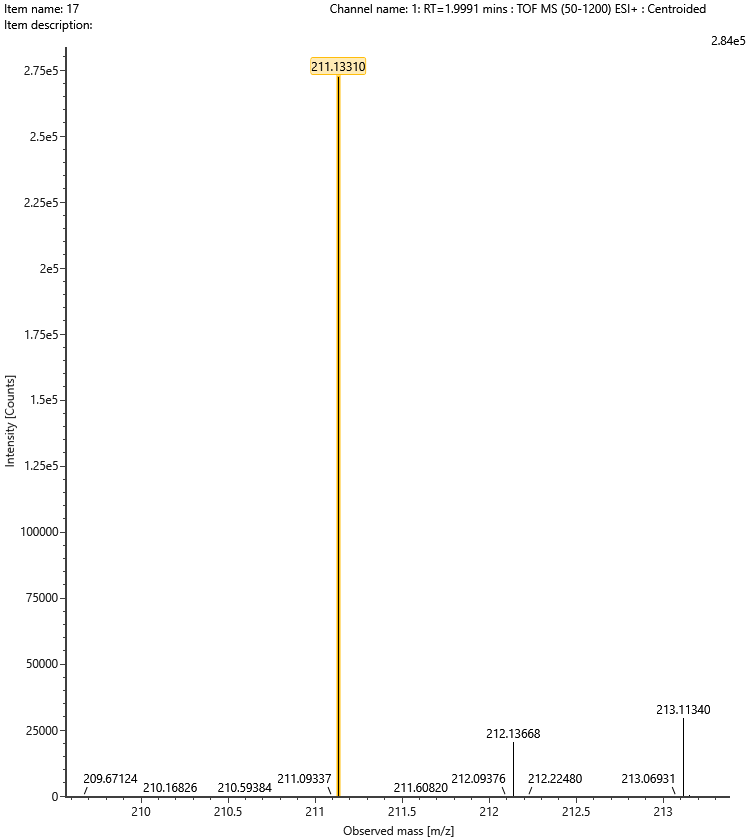


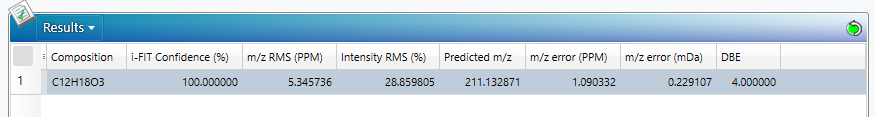


**Figure S20.** HRESIMS spectrum of **3**

**Figure S21.** UV spectrum of **3**


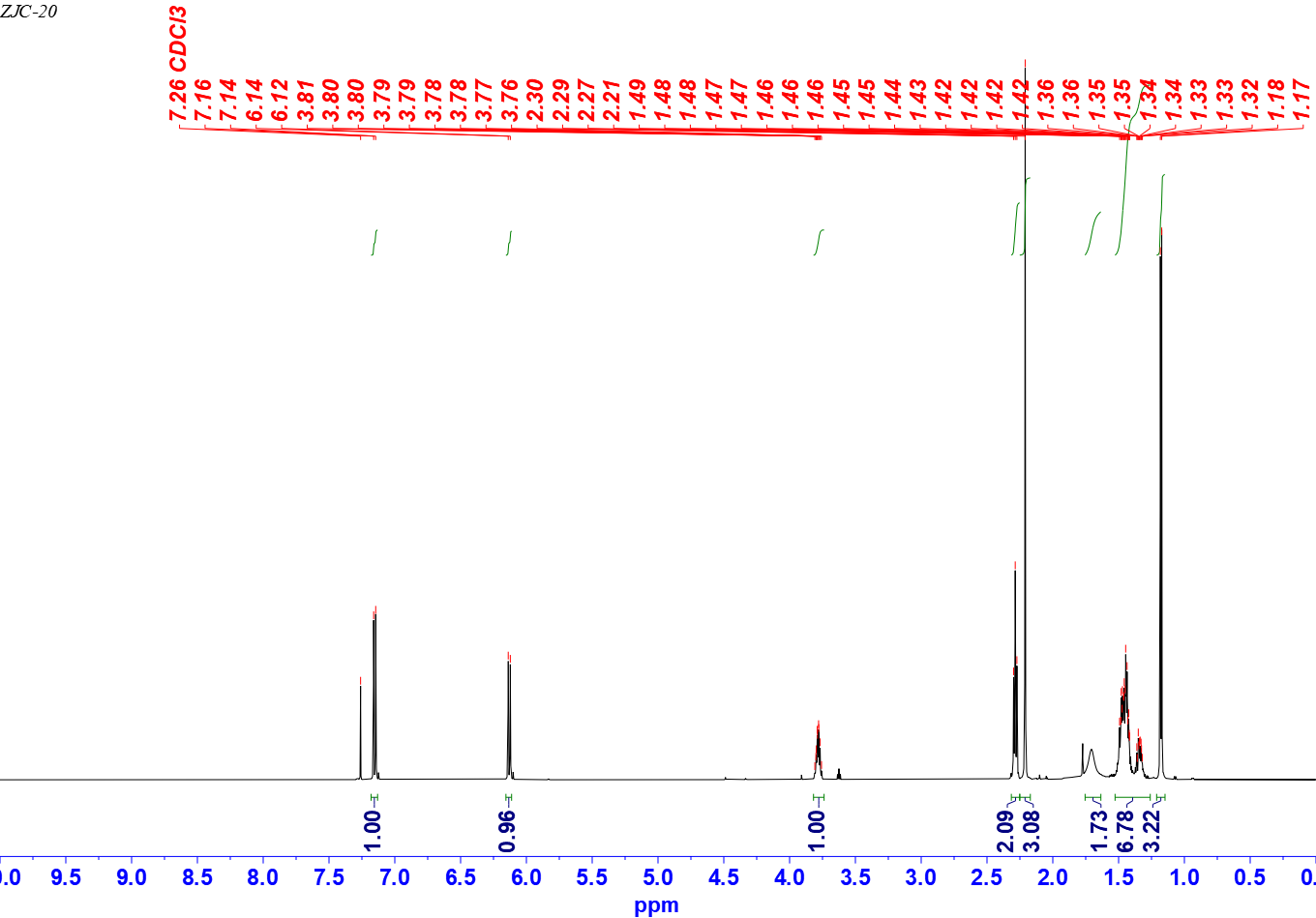


**Figure S22.** ^1^H NMR spectrum of **4**


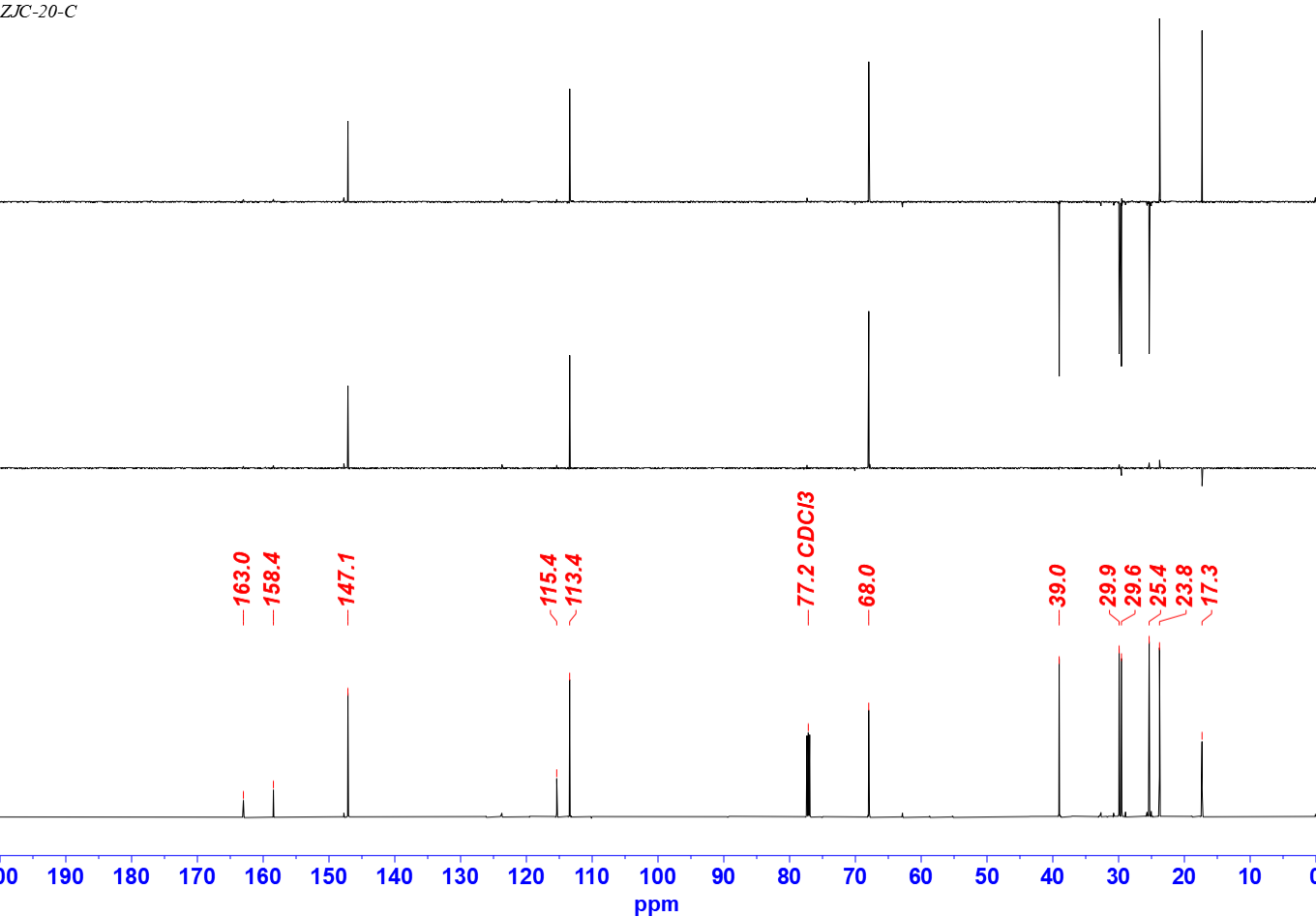


**Figure S23.** ^13^C NMR spectrum of **4**


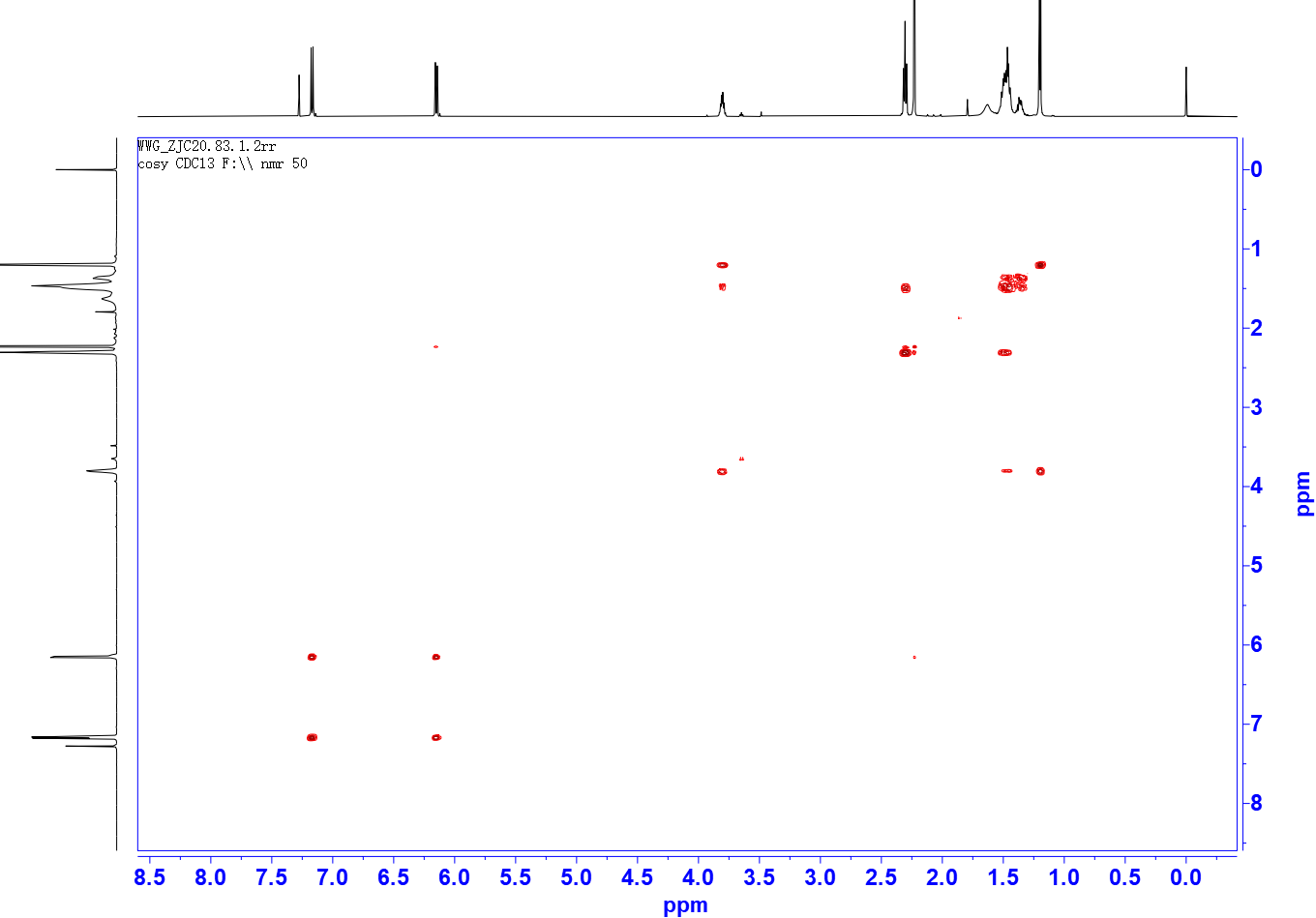


**Figure S24.** COSY spectrum of **4**


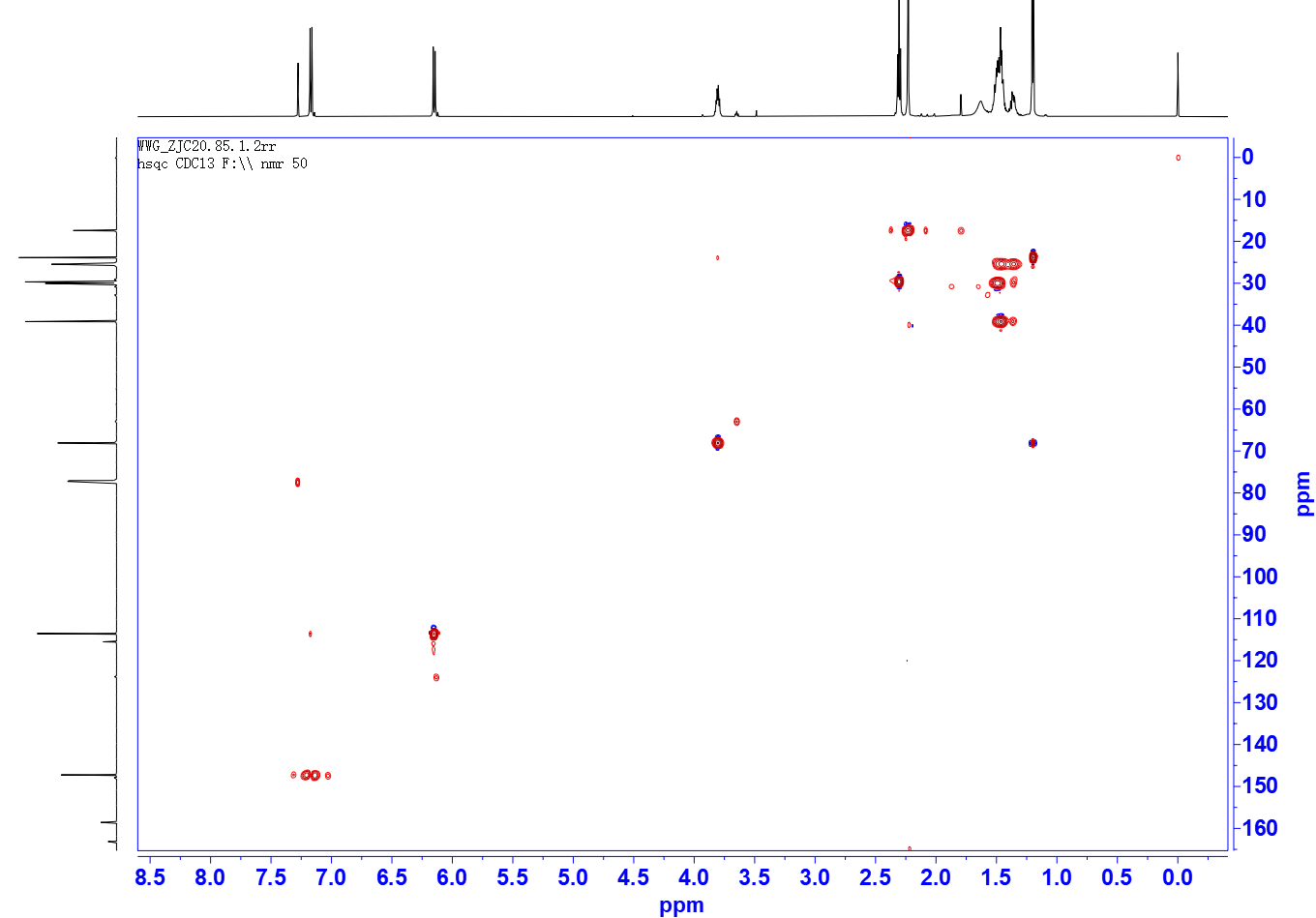


**Figure S25.** HSQC spectrum of **4**


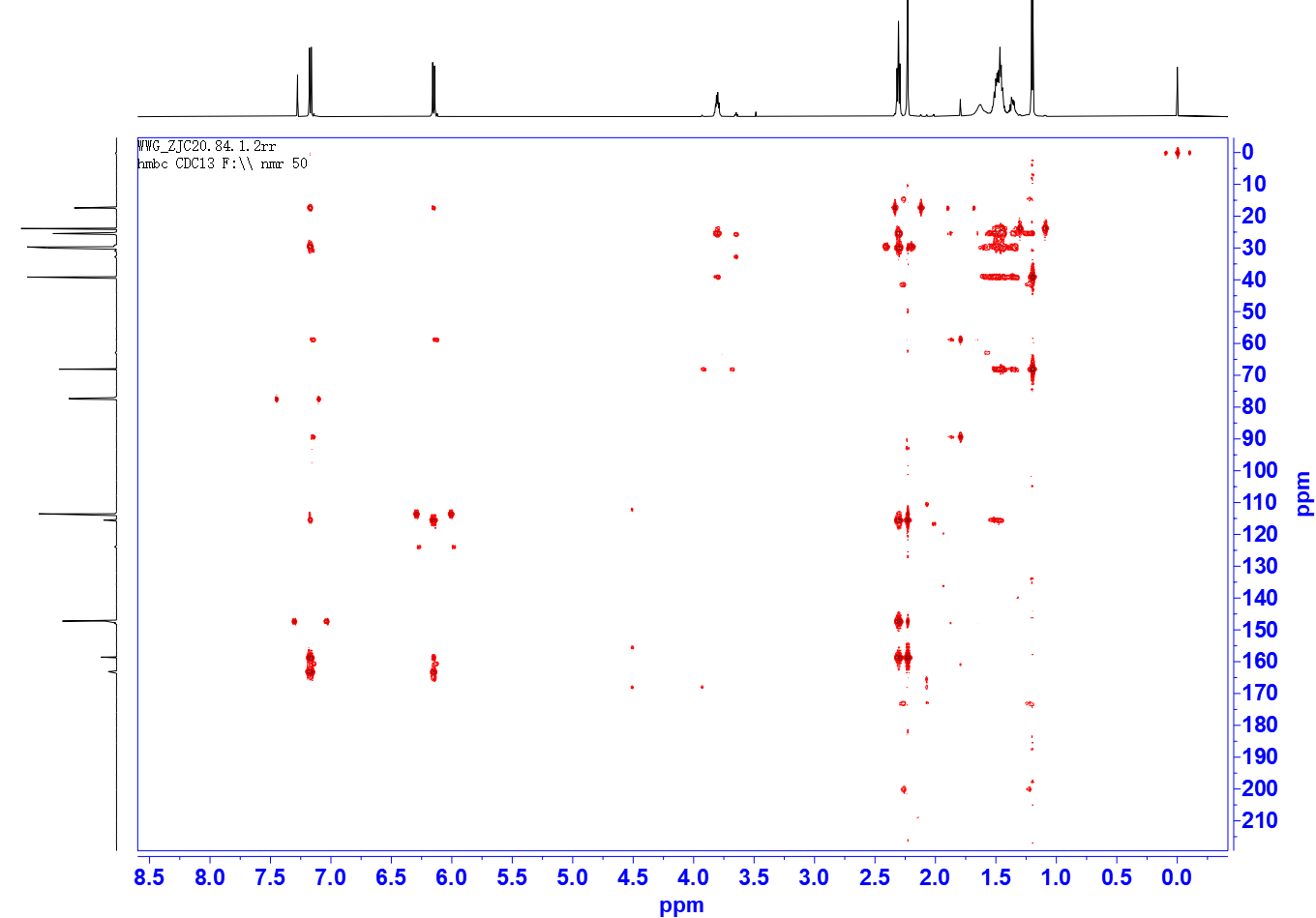


**Figure S26.** HMBC spectrum of **4**


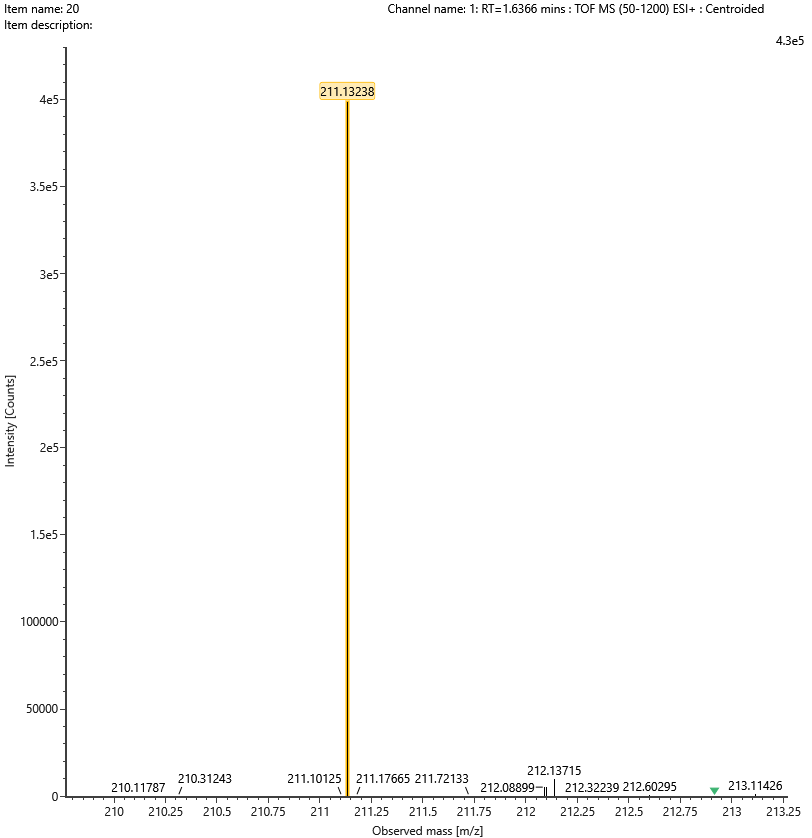


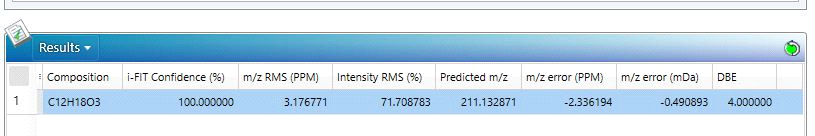


**Figure S27.** HRESIMS spectrum of **4**

**Figure S28.** UV spectrum of **4**


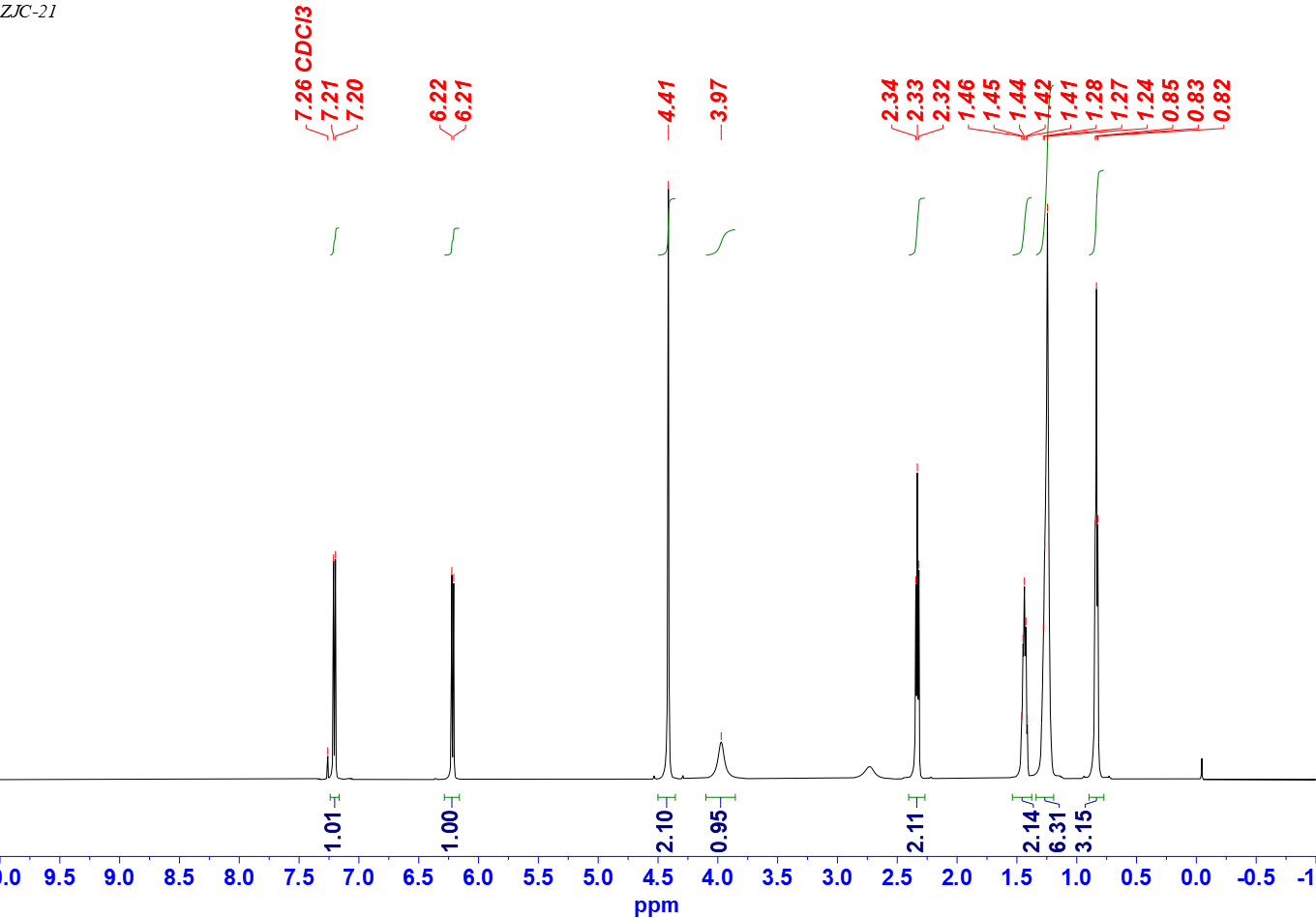


**Figure S29.** ^1^H NMR spectrum of **5**


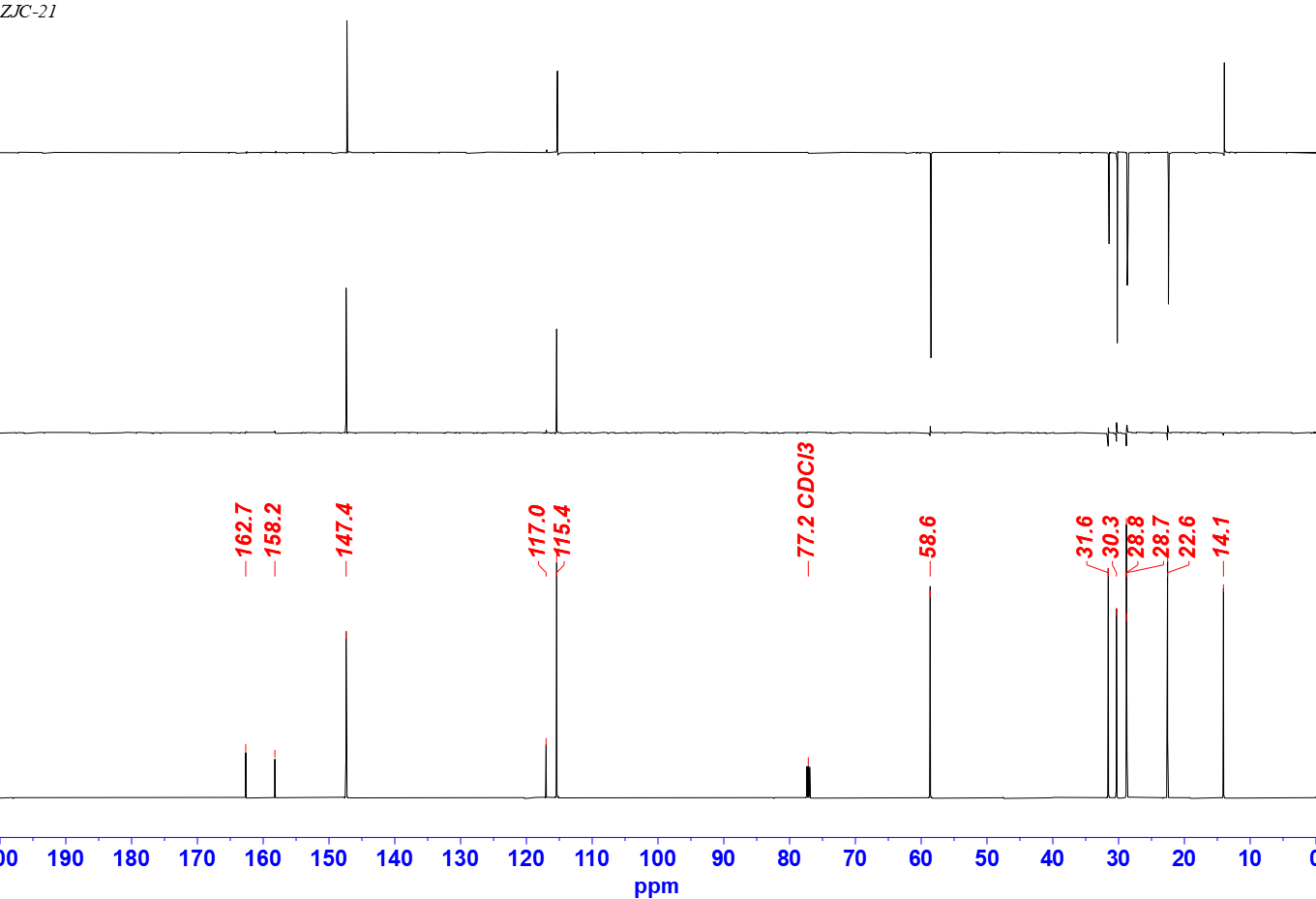


**Figure S30.** ^13^C NMR spectrum of **5**


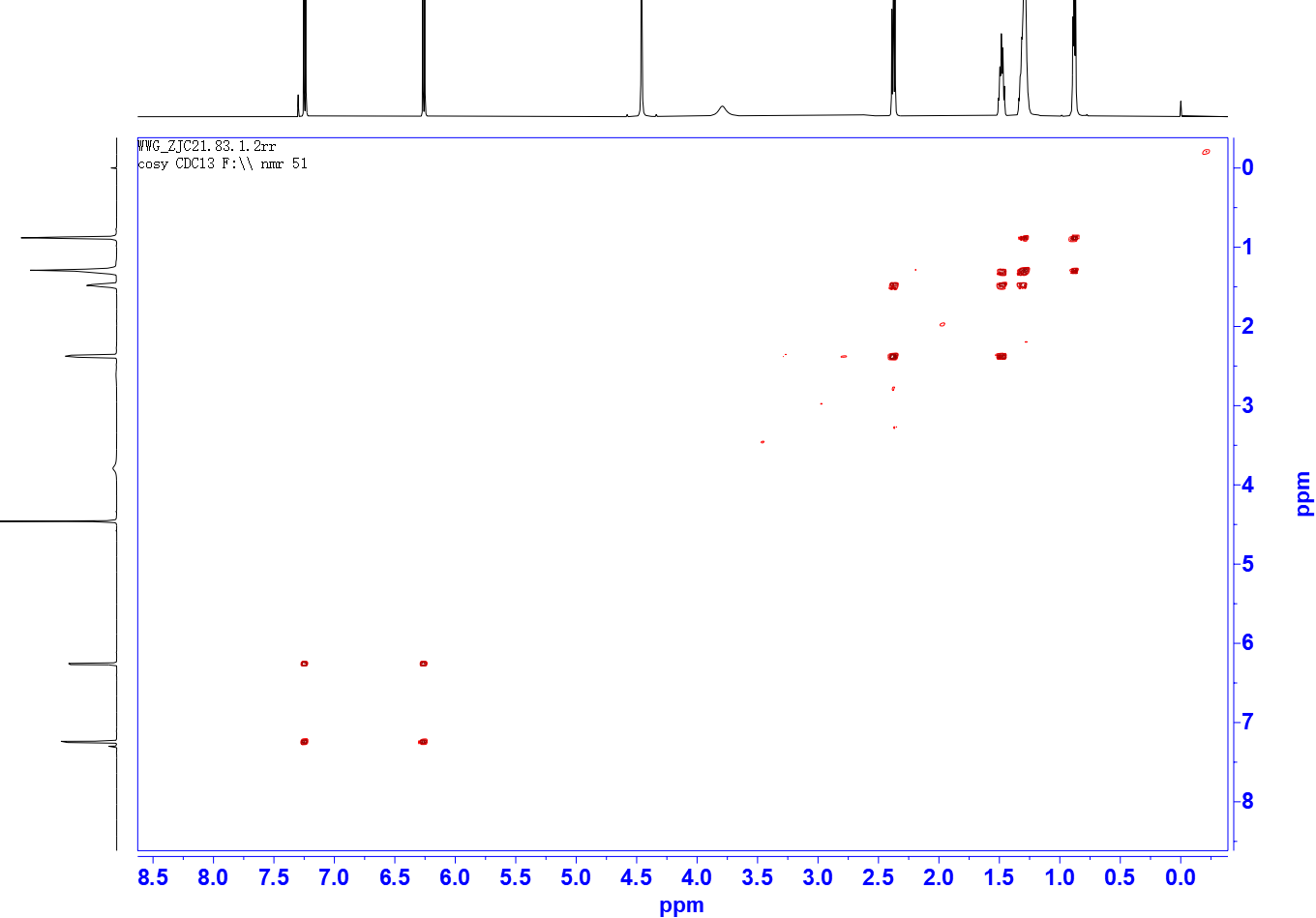


**Figure S31.** COSY spectrum of **5**


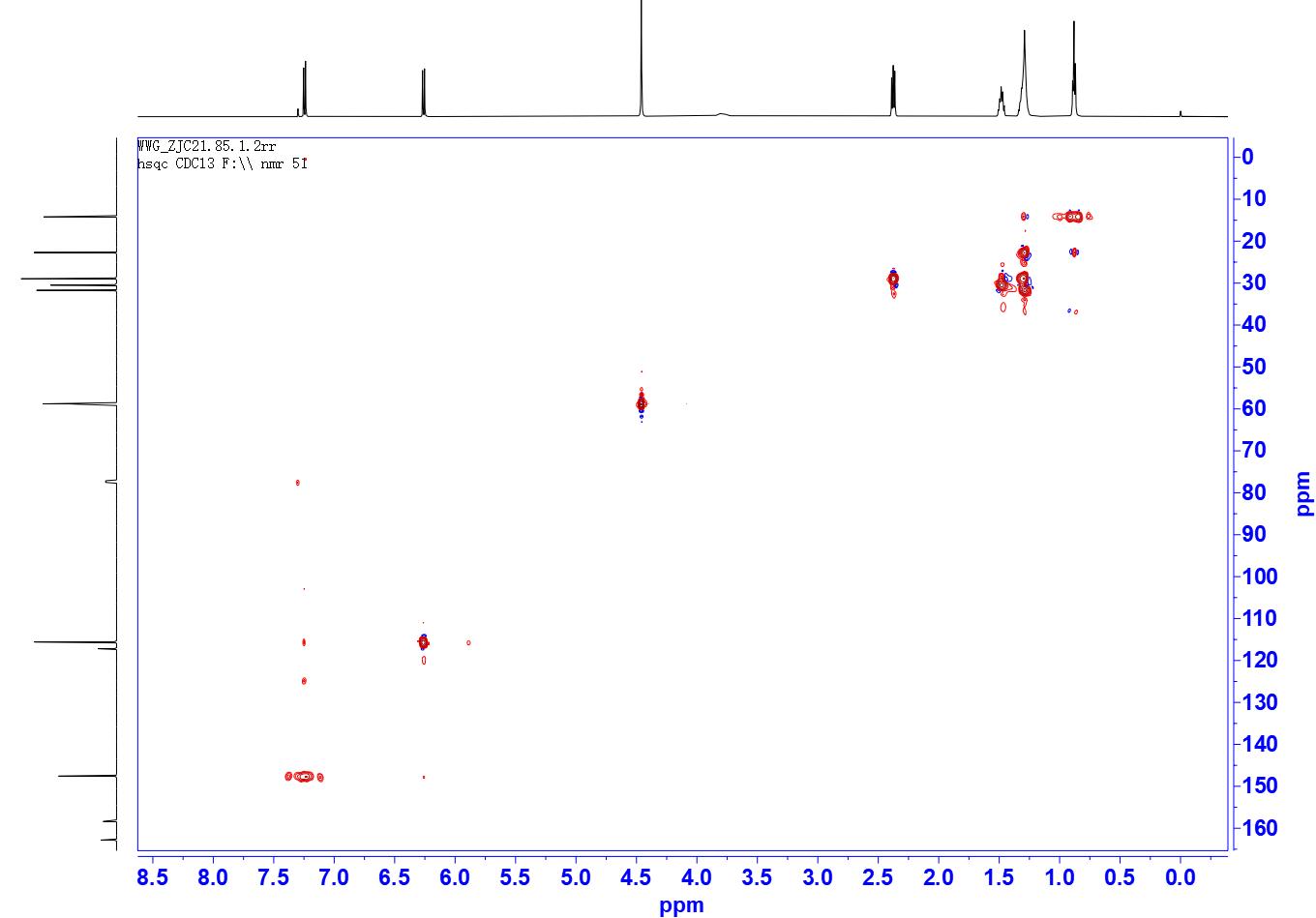


**Figure S32.** HSQC spectrum of **5**


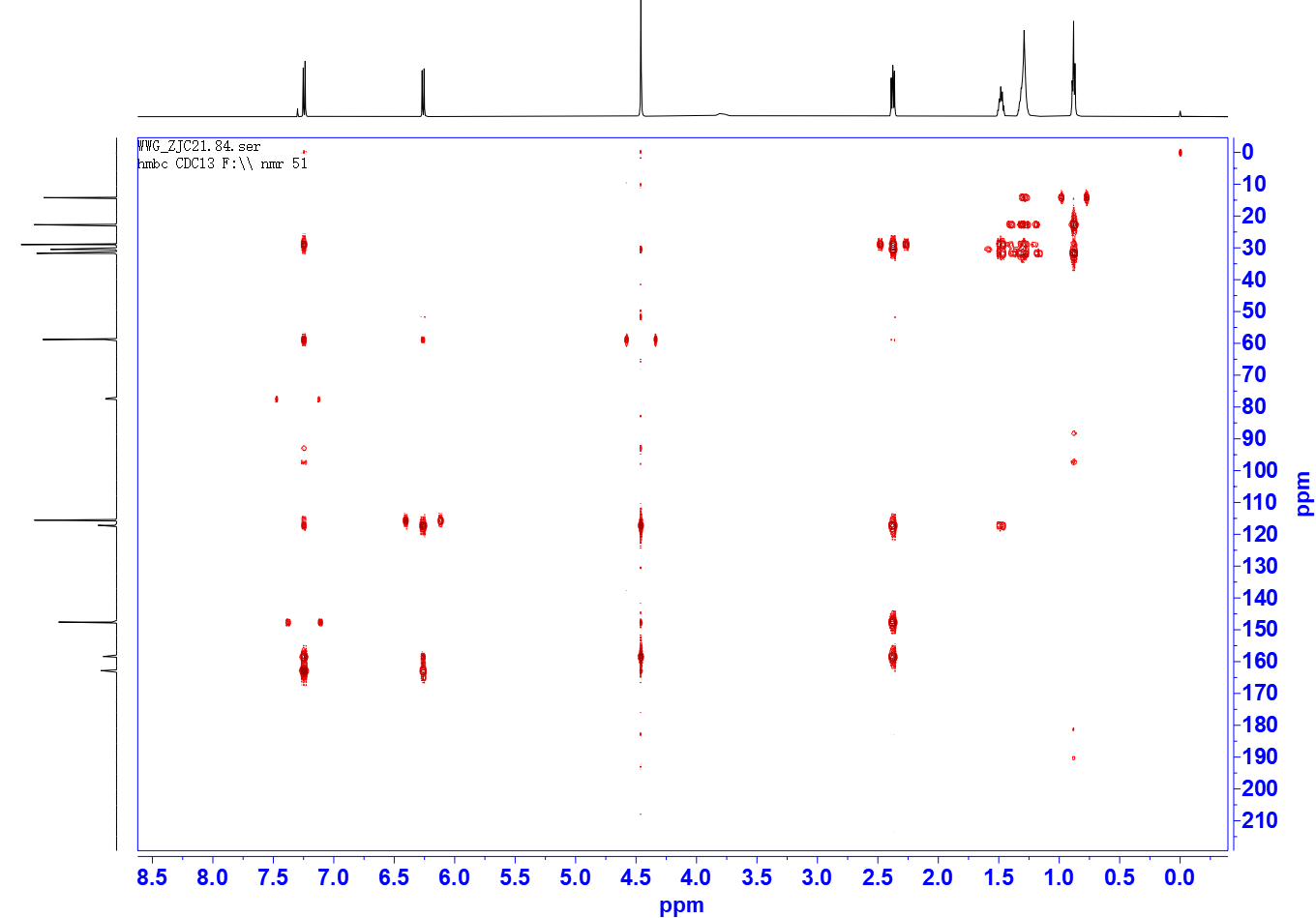


**Figure S33.** HMBC spectrum of **5**


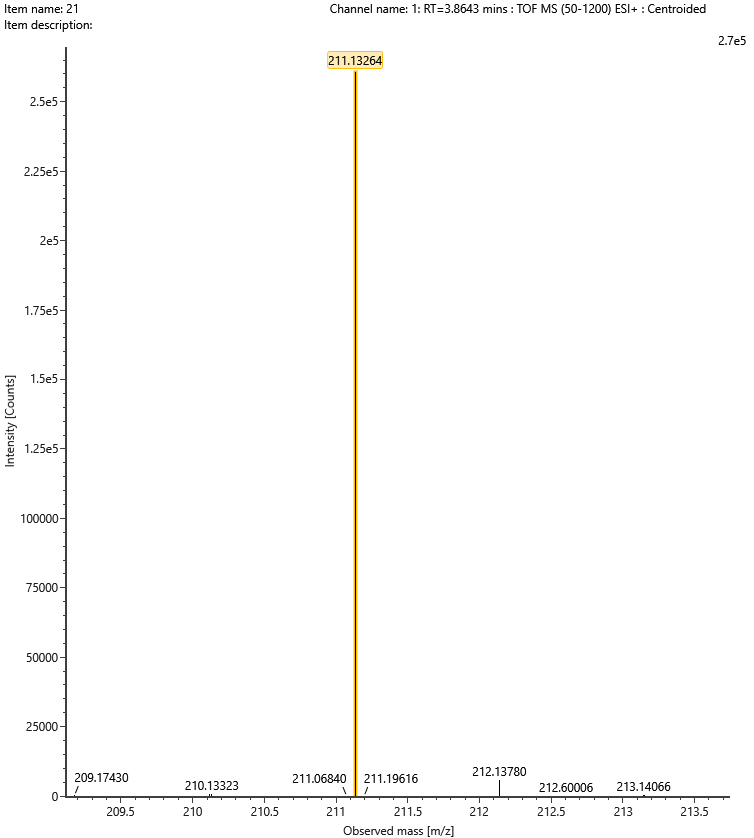

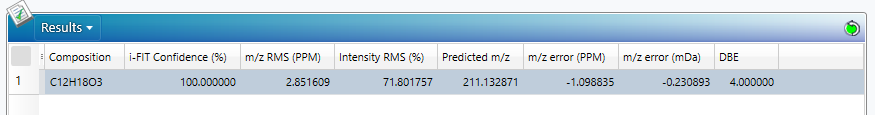


**Figure S34.** HRESIMS spectrum of **5**

**Figure S35.** UV spectrum of **5**

**
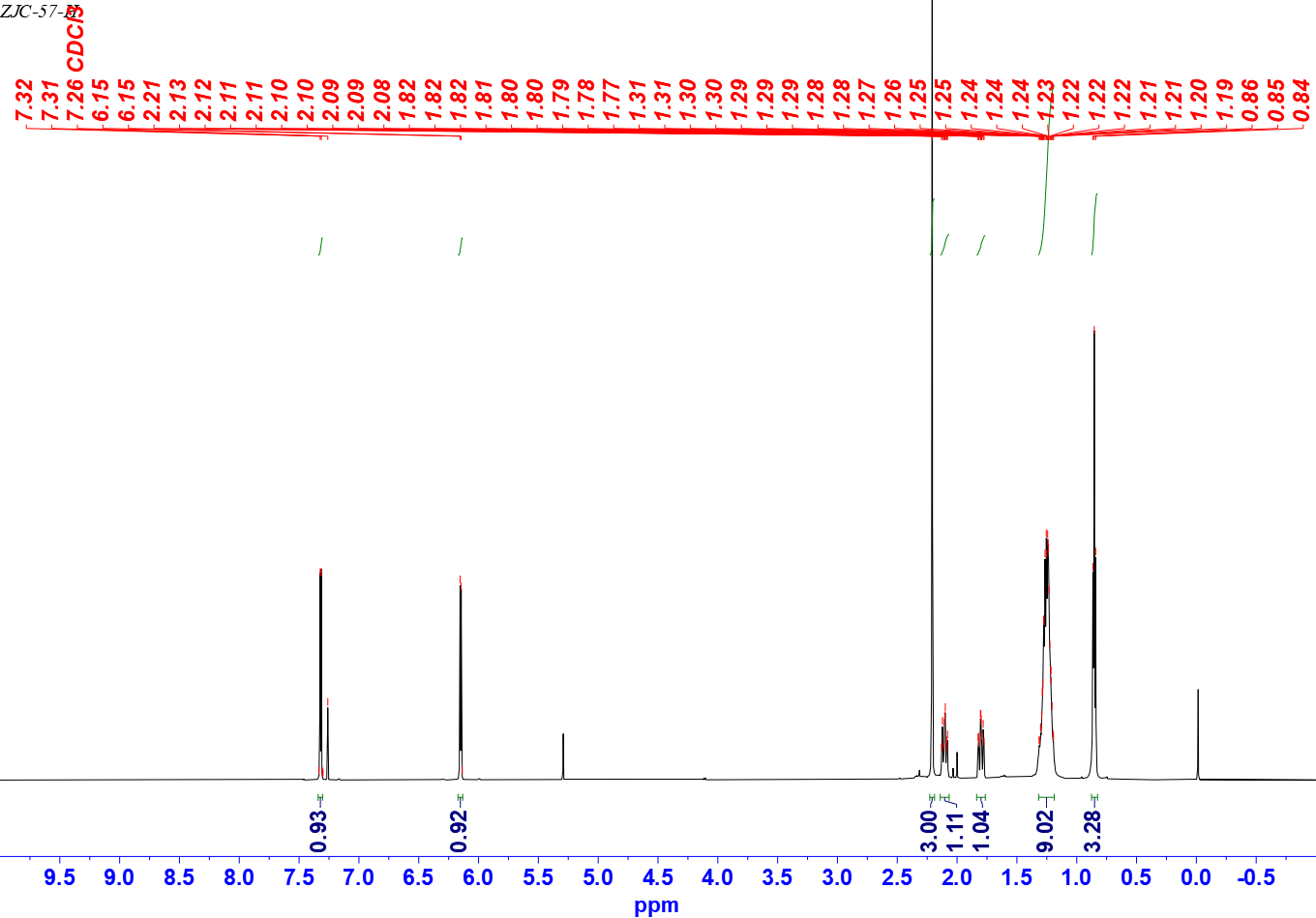
**

**Figure S36.** ^1^H NMR spectrum of **6**

**
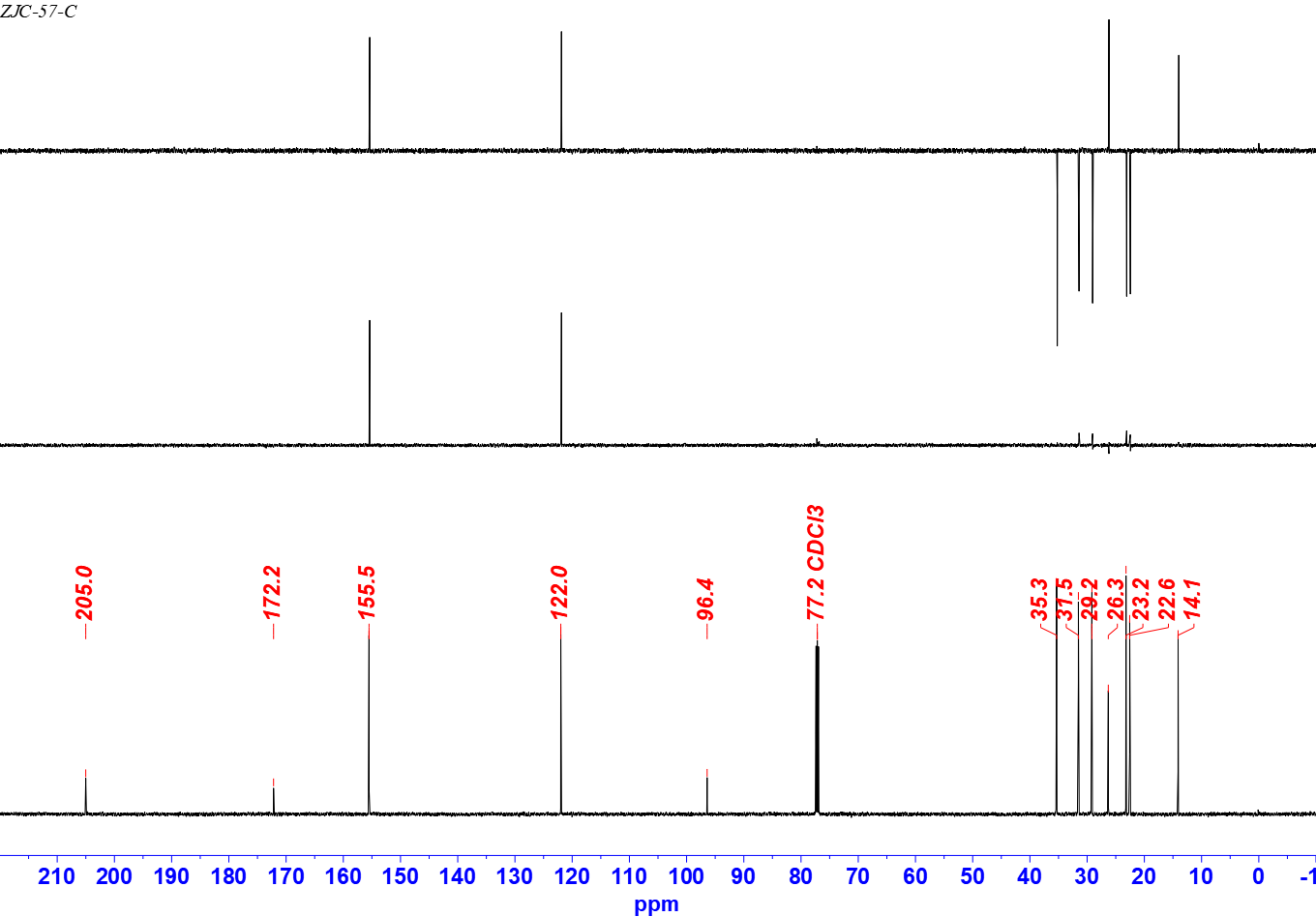
**

**Figure S37.** ^13^C NMR spectrum of **6**

**
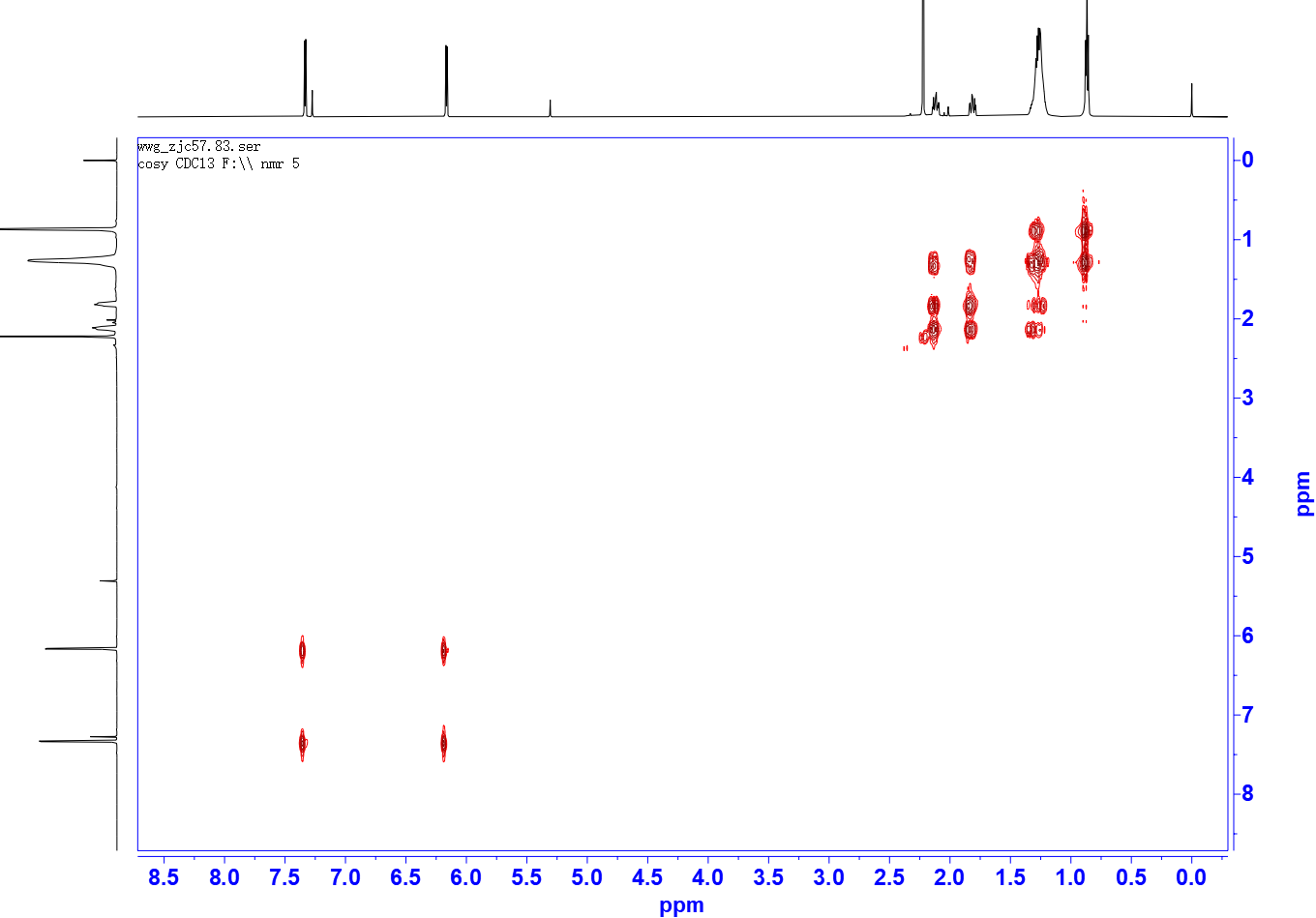
**

**Figure S38.** COSY spectrum of **6**

**
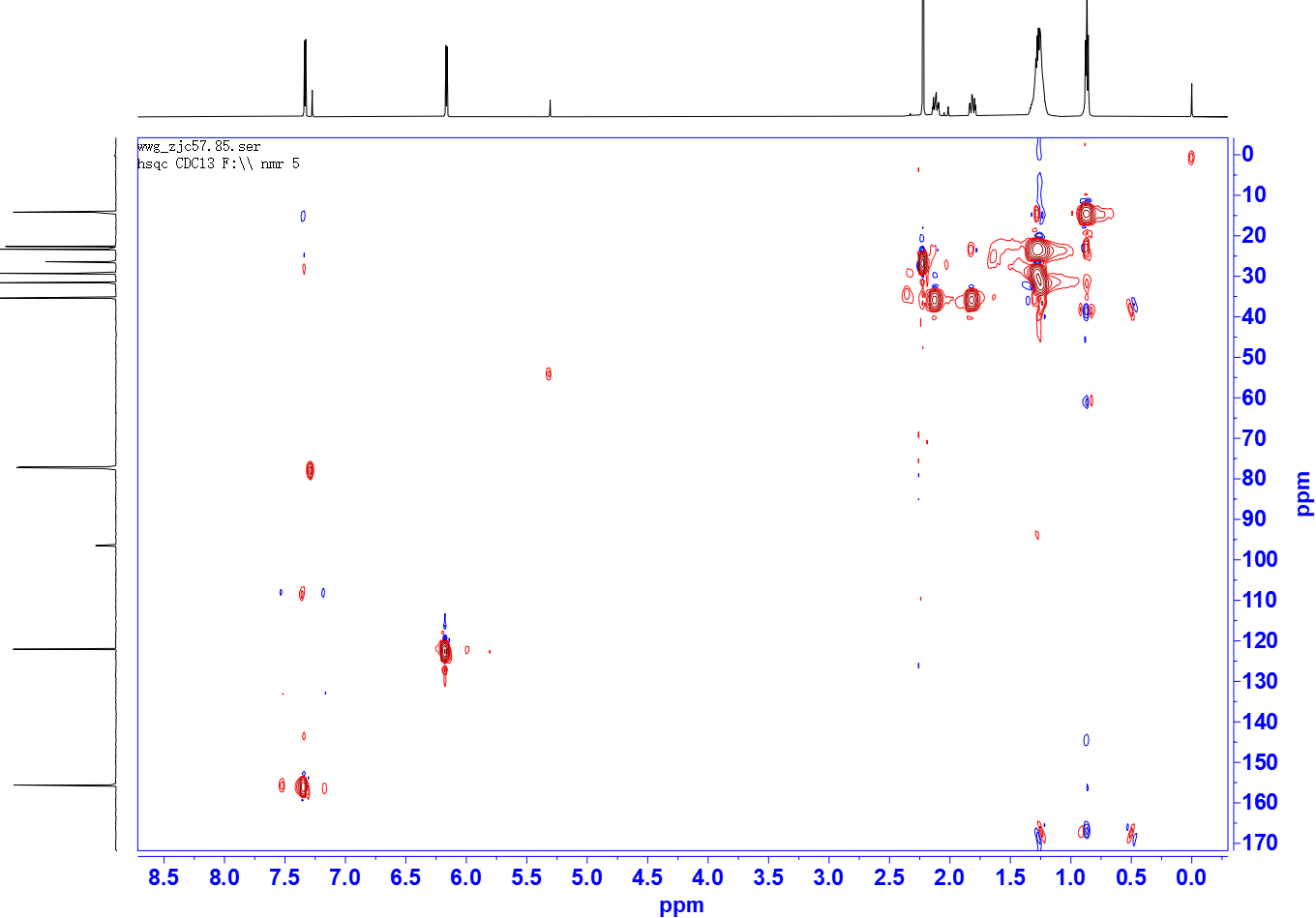
**

**Figure S39.** HSQC spectrum of **6**

**
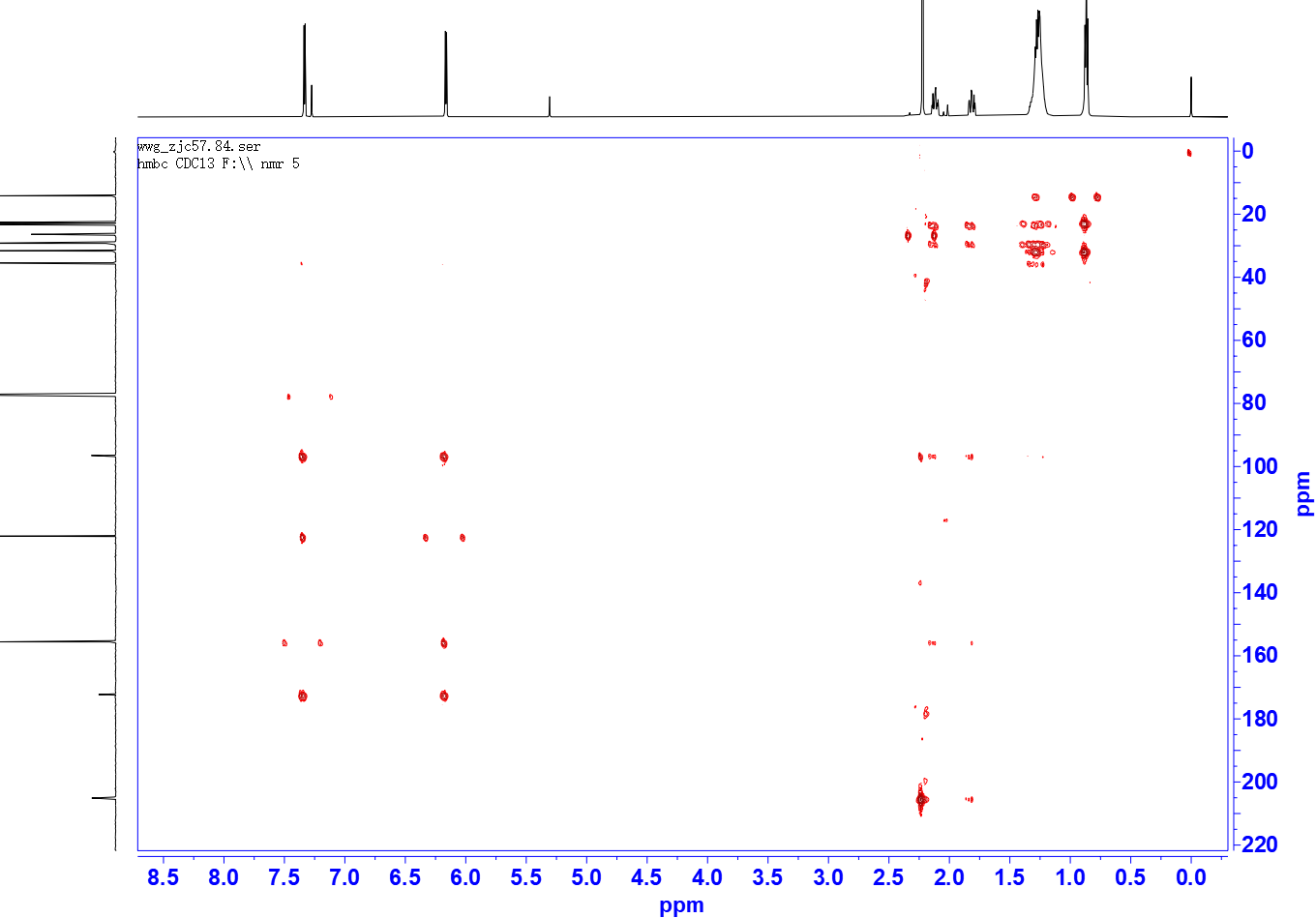
**

**Figure S40.** HMBC spectrum of **6**


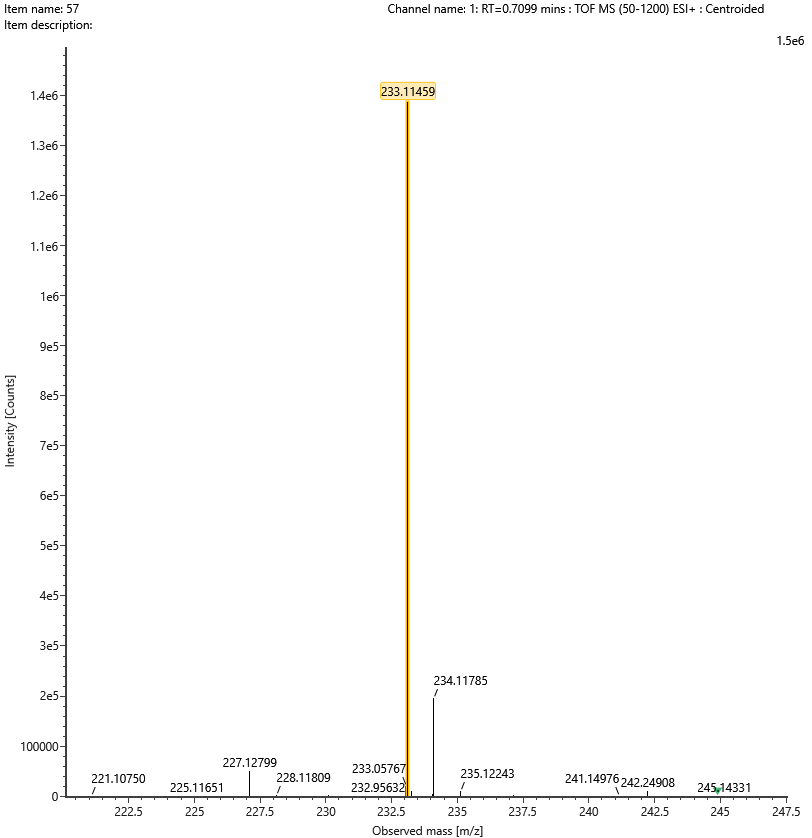
**
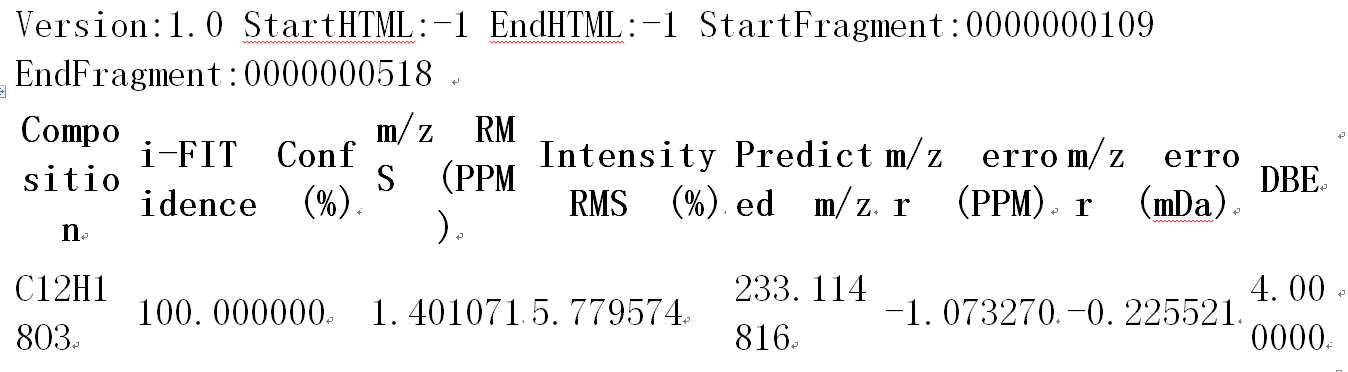
**

**Figure S41.** HRESIMS spectrum of **6**

**Figure S42.** UV spectrum of **6**


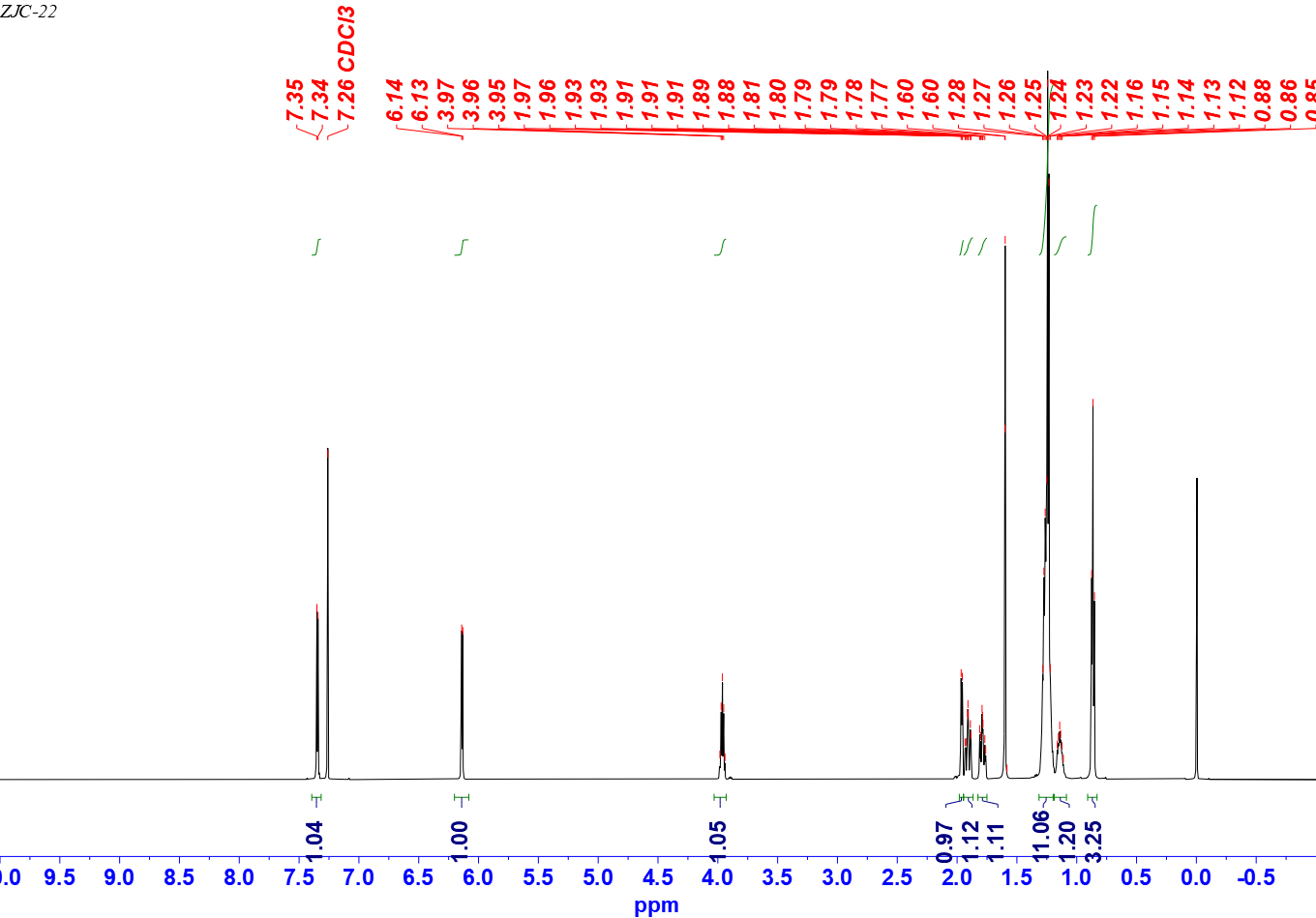


**Figure S43.** ^1^H NMR spectrum of **7**


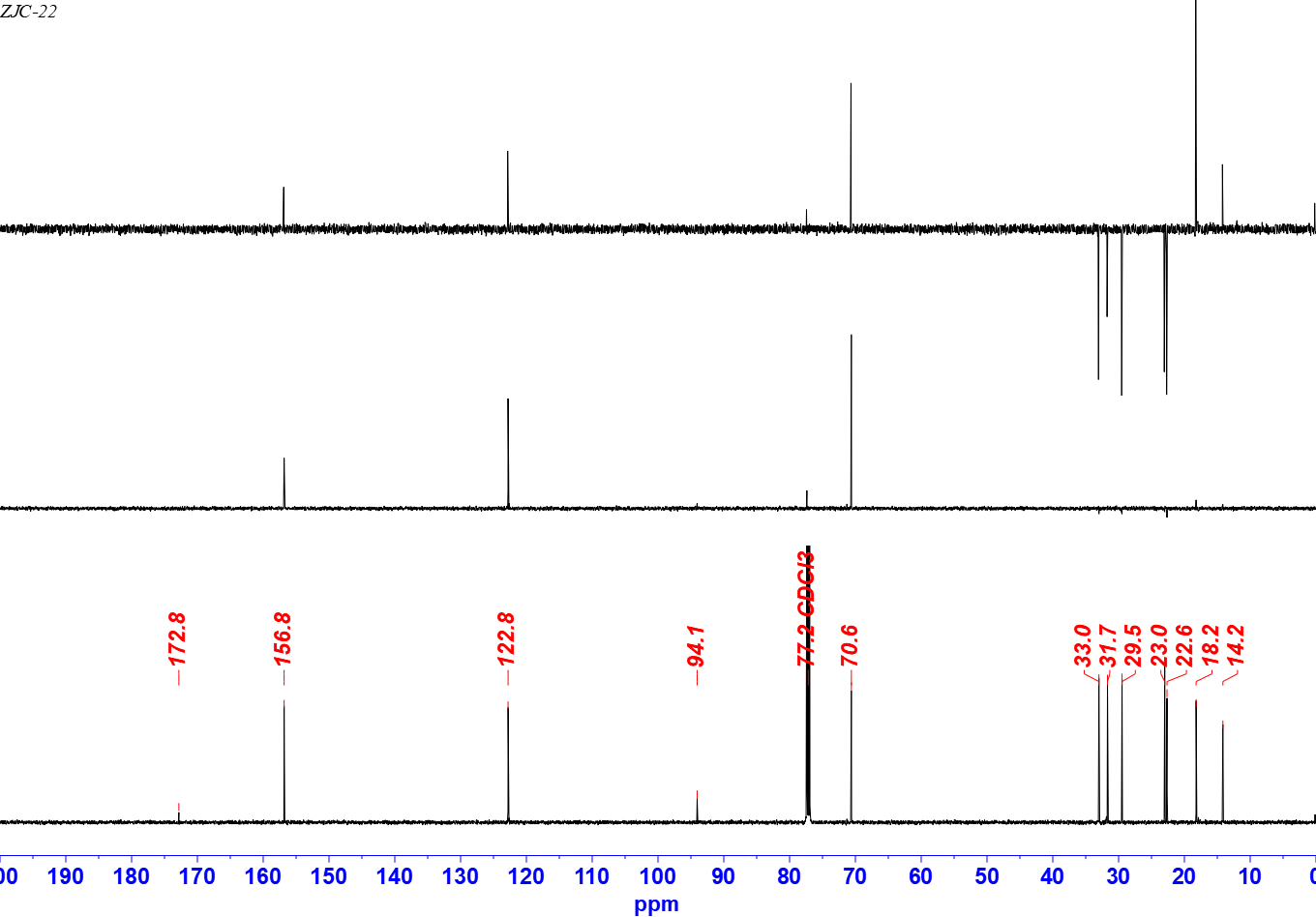


**Figure S44.** ^13^C NMR spectrum of **7**


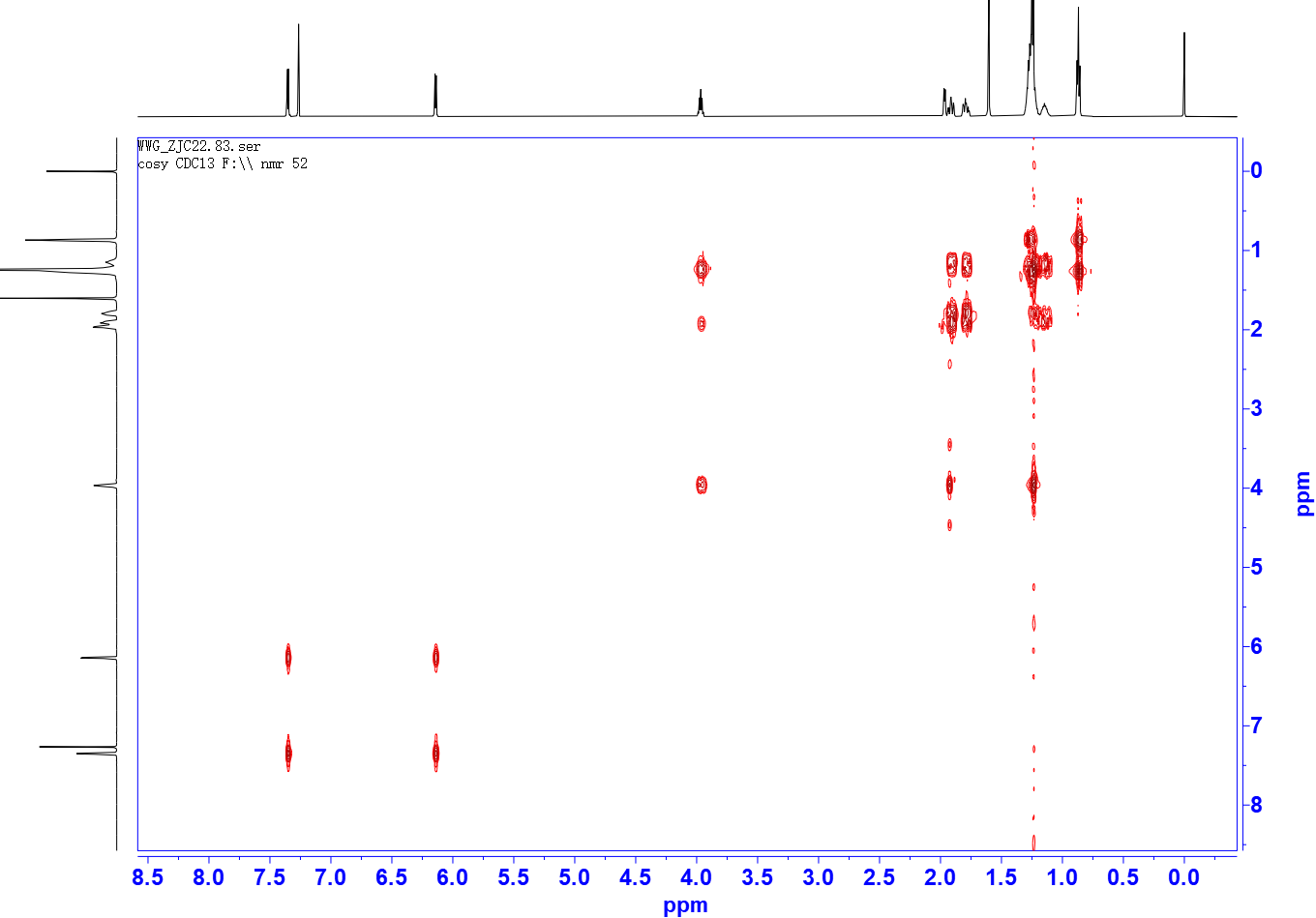


**Figure S45.** COSY spectrum of **7**


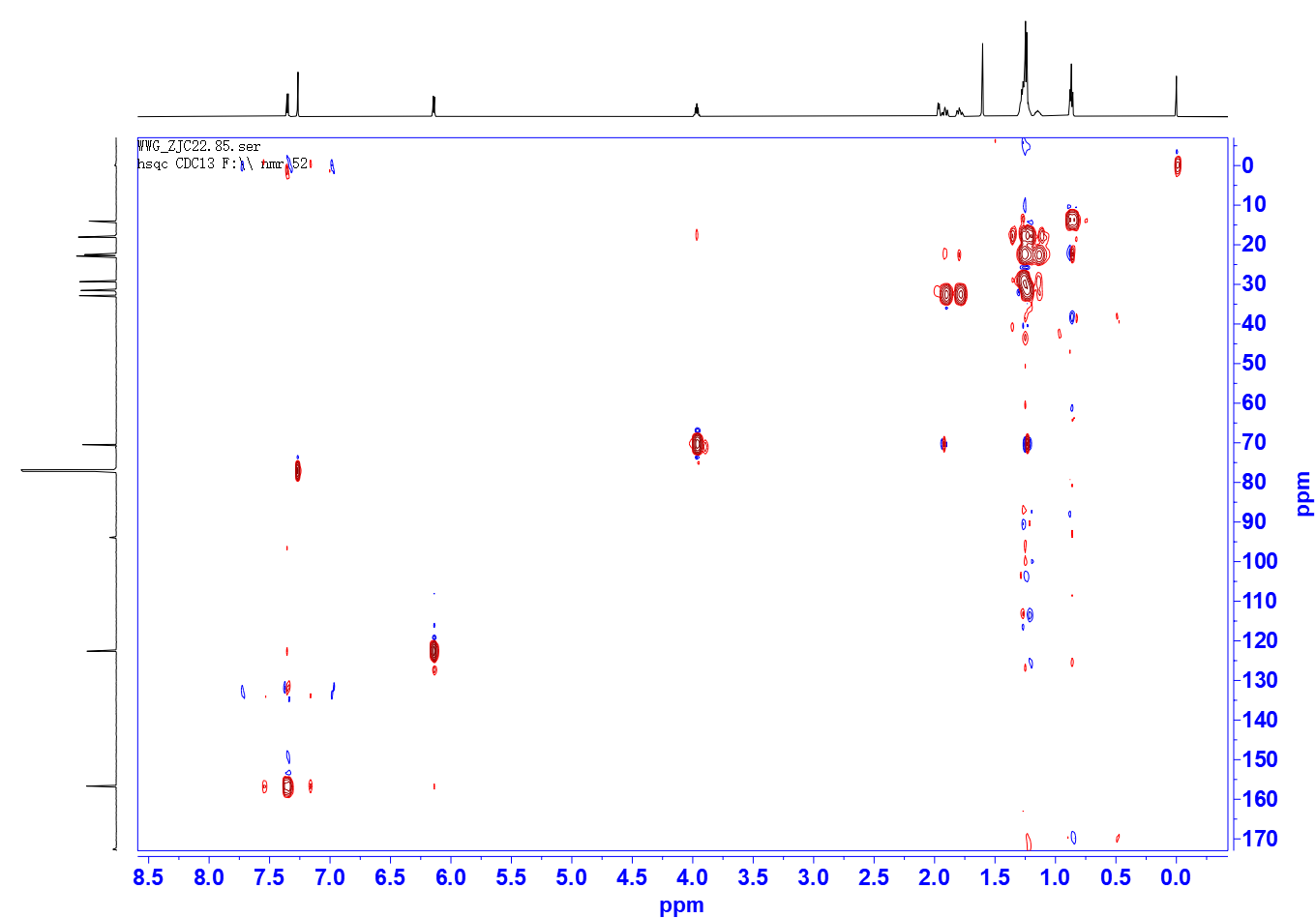


**Figure S46.** HSQC spectrum of **7**


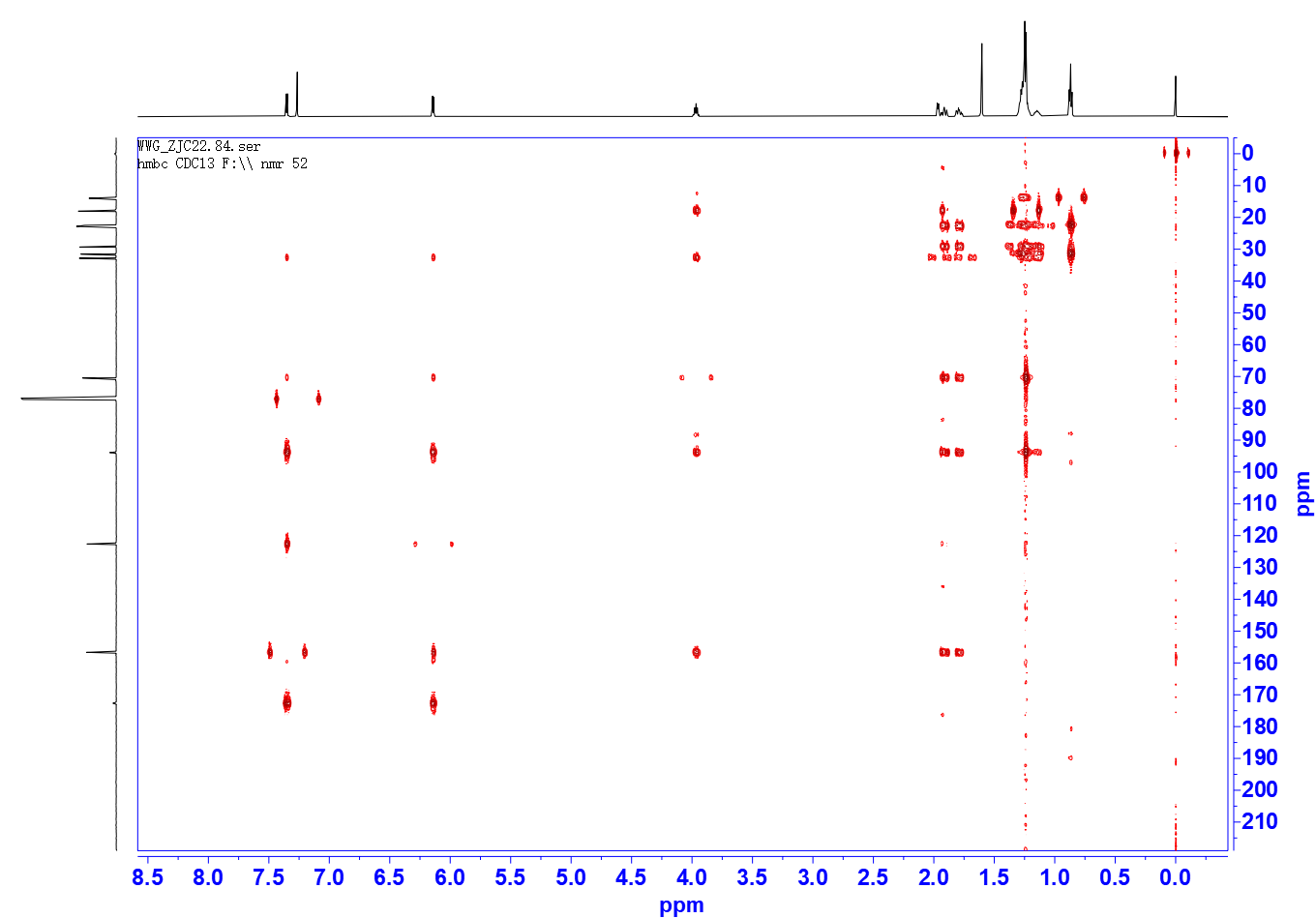


**Figure S47.** HMBC spectrum of **7**


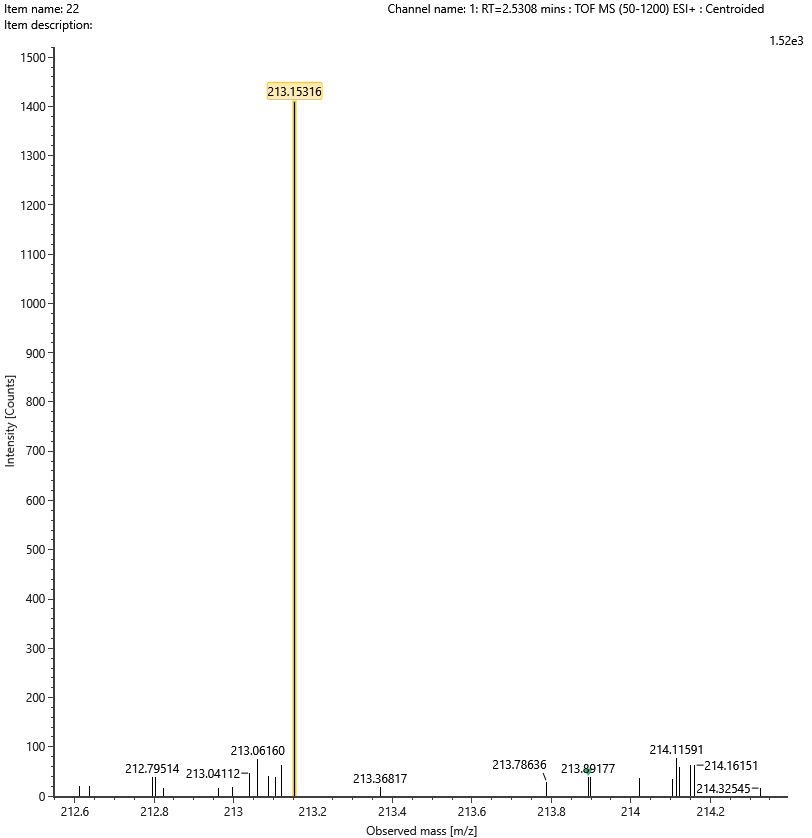


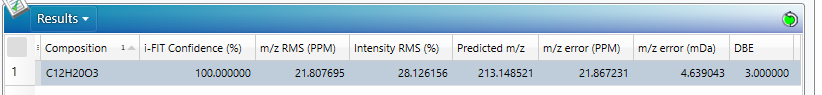


**Figure S48.** HRESIMS spectrum of **7**

**Figure S49.** UV spectrum of **7**


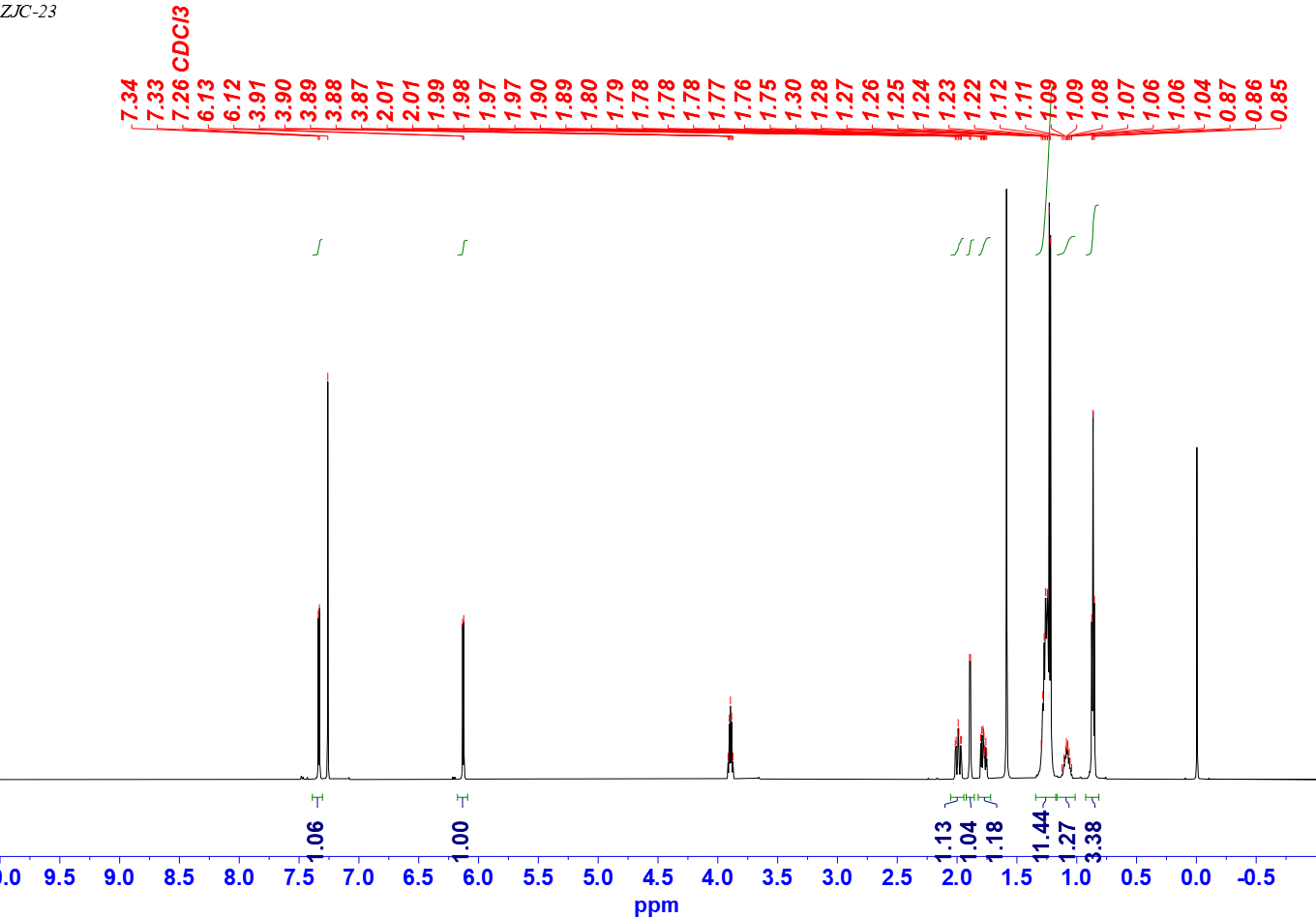


**Figure S50.** ^1^H NMR spectrum of **8**


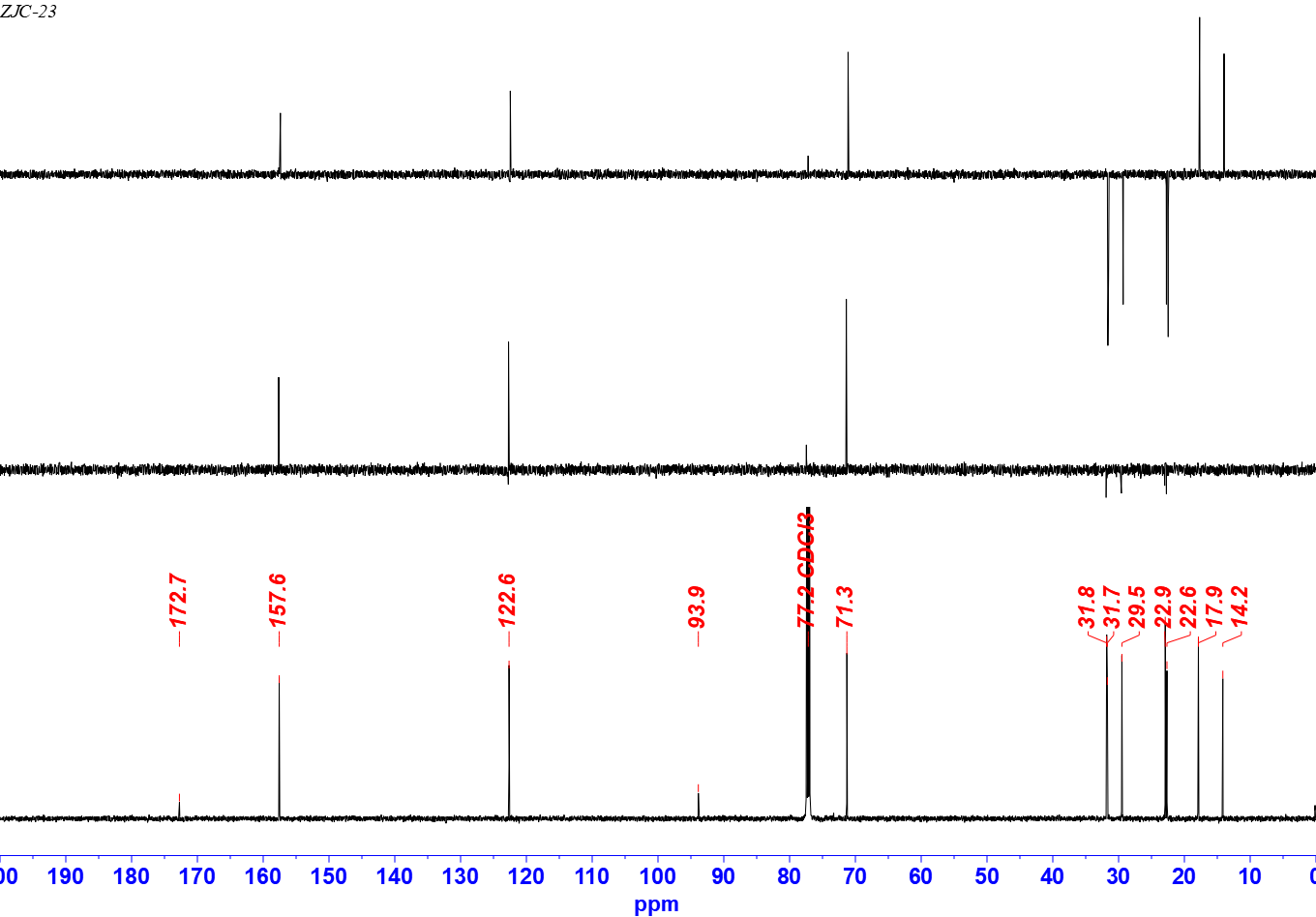


**Figure S51.** ^13^C NMR spectrum of **8**


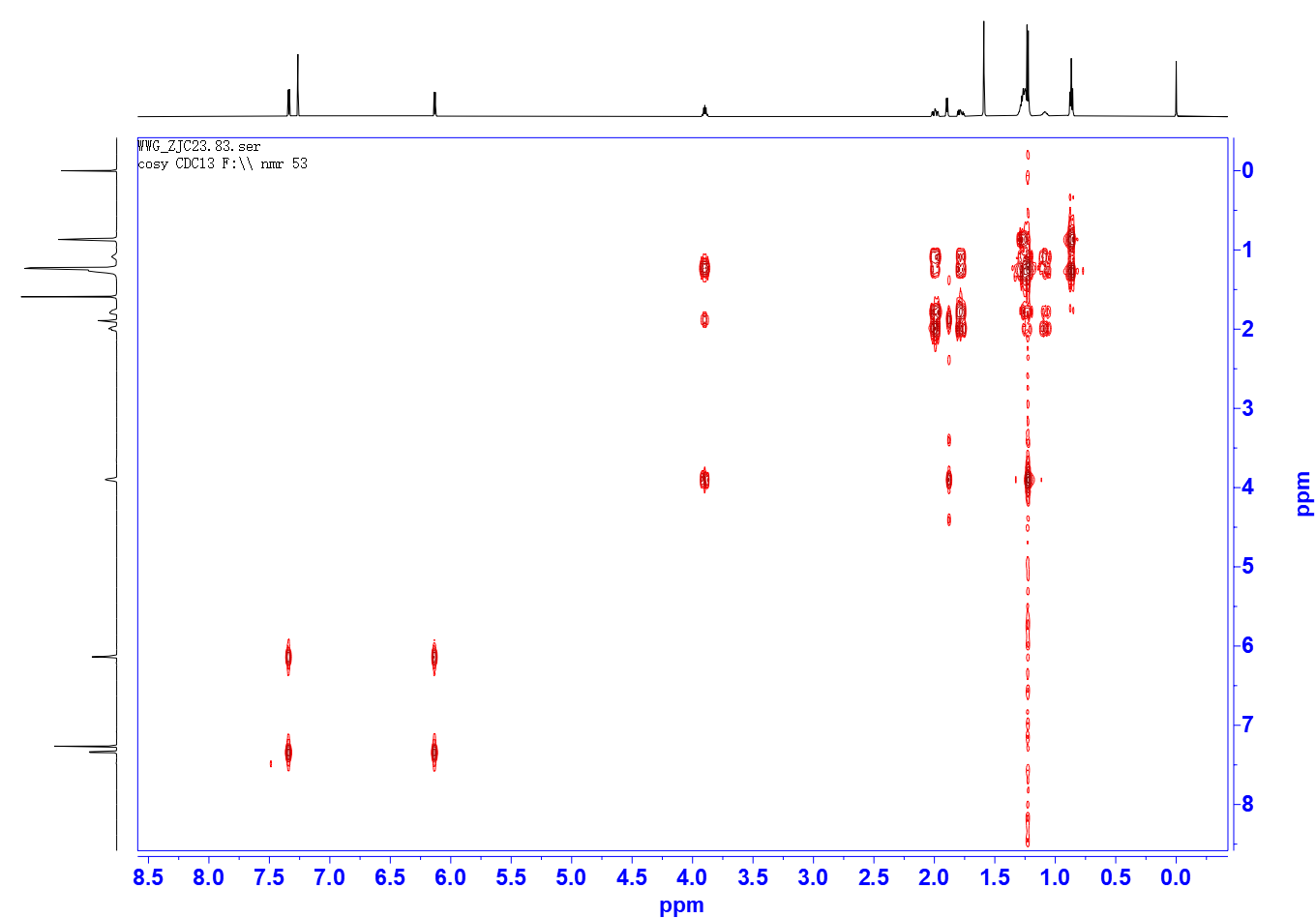


**Figure S52.** COSY spectrum of **8**


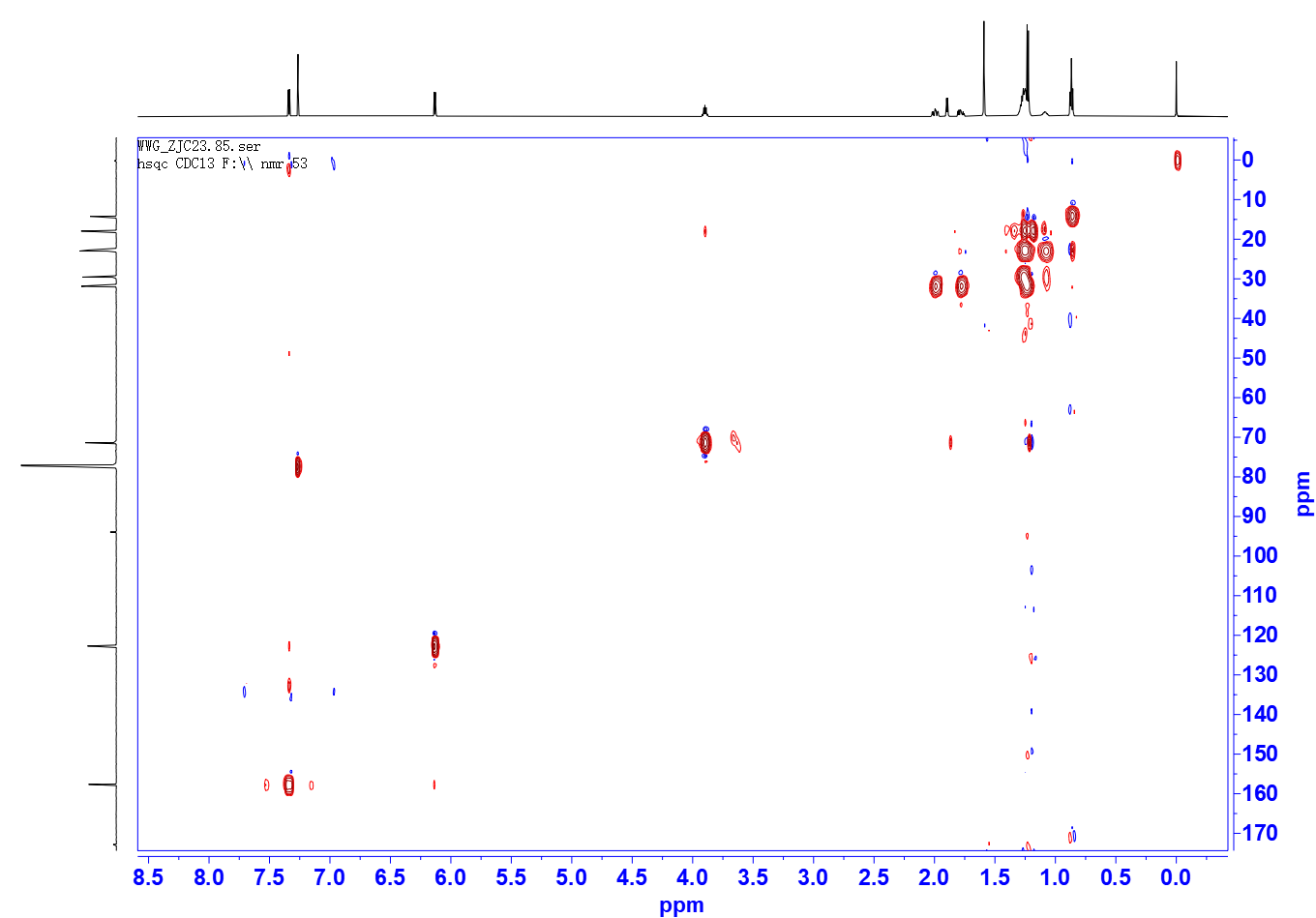


**Figure S53.** HSQC spectrum of **8**


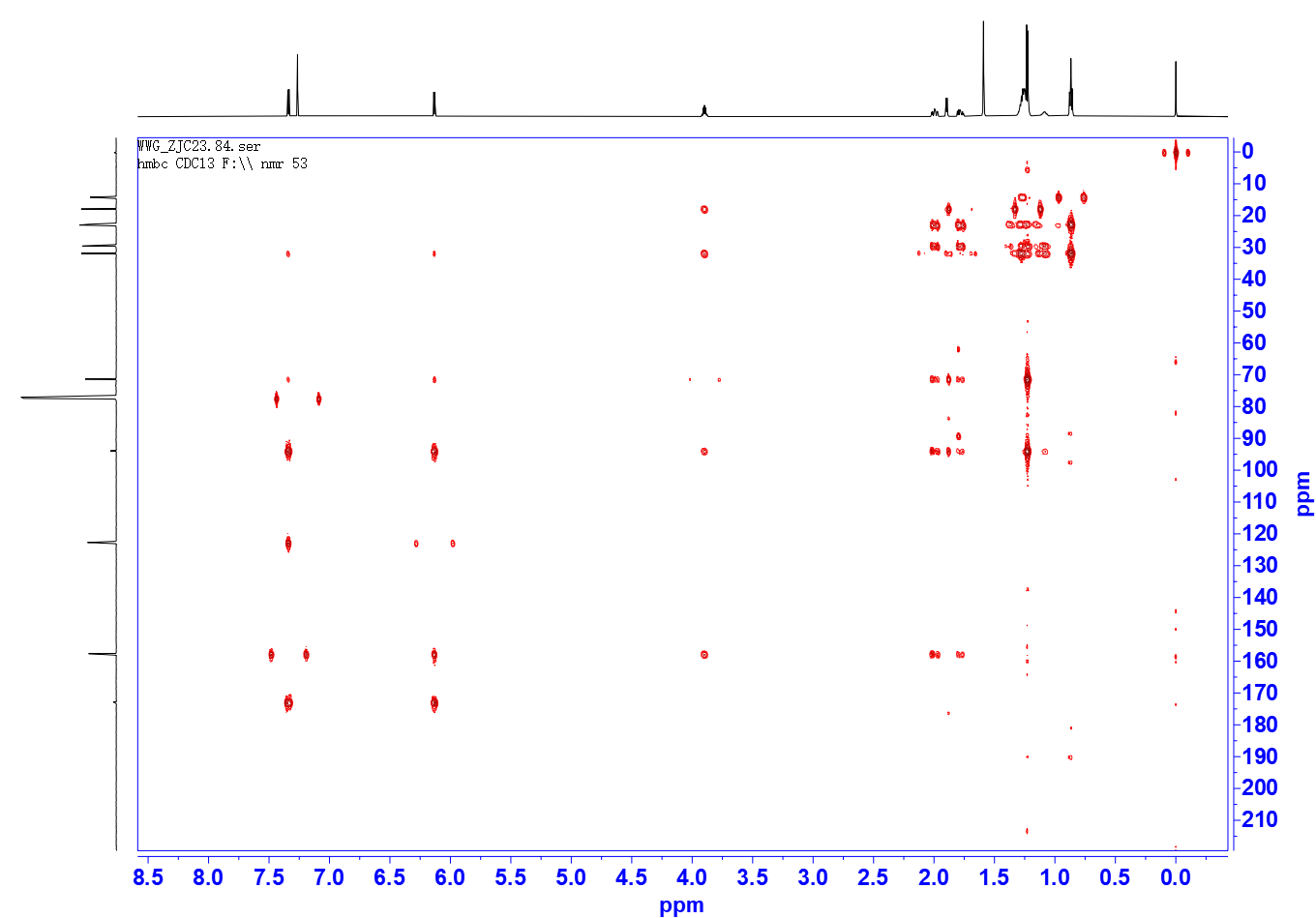


**Figure S54.** HMBC spectrum of **8**


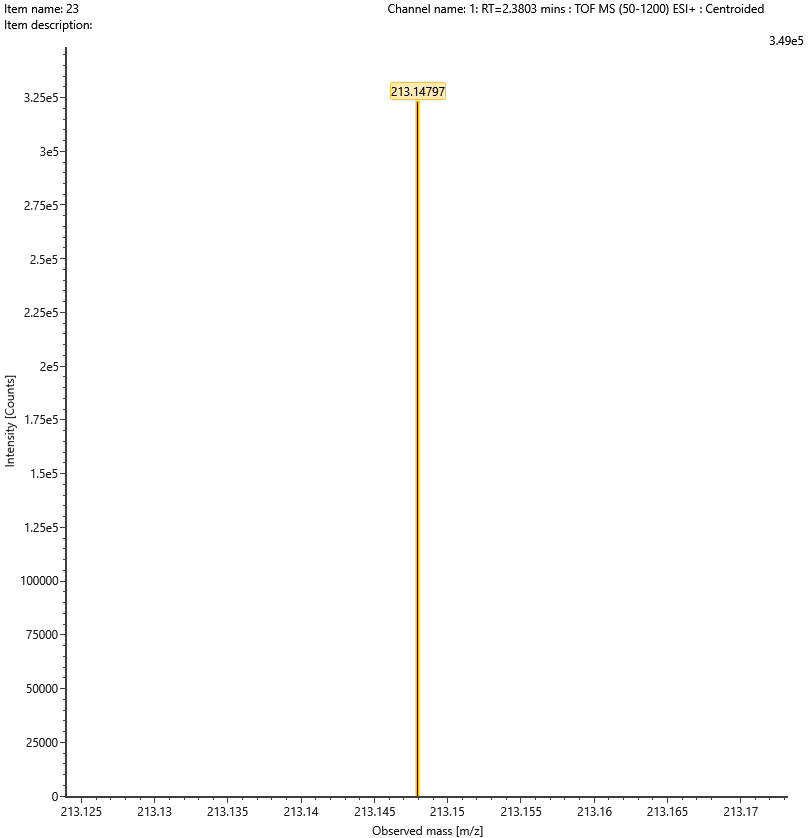

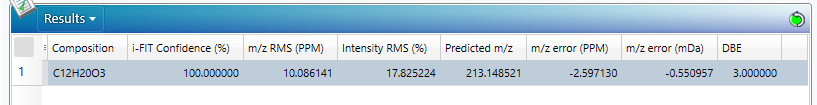


**Figure S55.** HRESIMS spectrum of **8**

**Figure S56.** UV spectrum of **8**

**Table S1**. Important thermodynamic parameters and Boltzmann distributions of the optimized **2** at B3LYP/6-31G(d) level in gas phase.

| Conformations | Energy (a.u) | Δ G(kcal/mol) | % |
| --- | --- | --- | --- |
| 1 | -693.575101 | 0.00 | 64.98% |
| 2 | -693.572997 | 1.32 | 7.00% |
| 3 | -693.571868 | 2.03 | 2.12% |
| 4 | -693.573612 | 0.93 | 13.42% |
| 5 | -693.573544 | 0.98 | 12.49% |

**Table S2**. The coordinates for the lowest energy conformers of **2**

| 33  Energy = -693.575101 a.u. #Cluster: 1  C -0.48118 3.71268 -1.82872  C -1.87716 3.64534 -2.3044  O -2.4575 2.36911 -2.45027  C -1.70132 1.18425 -2.39362  C -0.23351 1.27279 -1.97123  C 0.28352 2.62967 -1.65383  O -2.6275 4.55455 -2.53516  O -0.65125 1.13089 -3.38704  C -2.63043 0.03009 -2.24482  C 0.50896 0.08527 -1.41491  C 0.33468 -0.02361 0.10468  C 1.0695 -1.24979 0.66112  C 0.90479 -1.35587 2.18282  C 1.63361 -2.58611 2.74152  C 1.47925 -2.69274 4.25662  H -0.13201 4.72816 -1.61688  H 1.30573 2.68846 -1.27235  H -3.67515 0.34077 -2.4347  H -2.39531 -0.76548 -2.96955  H -2.59471 -0.39758 -1.23357  H 0.15642 -0.84318 -1.9143  H 1.58464 0.15622 -1.68167  H 0.709 0.89515 0.59779  H -0.7422 -0.08141 0.35897  H 0.69011 -2.17086 0.17704  H 2.14522 -1.1965 0.40281  H 1.28981 -0.43981 2.67115  H -0.16939 -1.40652 2.4462  H 1.24464 -3.5054 2.26117  H 2.70864 -2.53964 2.47838  H 1.99988 -3.57497 4.64643  H 1.89306 -1.81649 4.76858  H 0.42716 -2.77745 4.55173  33  Energy = -693.572997 a.u. #Cluster: 2  C 3.02218 -0.80121 -1.66684  C 2.97058 0.47455 -2.40713  O 1.70055 0.99308 -2.72917  C 0.51715 0.24692 -2.5769  C 0.59064 -1.10307 -1.86086  C 1.93554 -1.53082 -1.39294  O 3.88594 1.18048 -2.73431  O 0.51481 -0.98302 -3.3382  C -0.64337 1.17423 -2.68618  C -0.6181 -1.74678 -1.23066  C -0.66186 -1.59013 0.2935  C -0.82014 -0.13011 0.73609  C -0.90338 -0.01538 2.26392  C -1.04342 1.44699 2.70974  C -1.13228 1.56691 4.22908  H 4.02867 -1.0905 -1.34881  H 1.98317 -2.4607 -0.82137  H -1.08043 1.40264 -1.70443  H -0.33327 2.13545 -3.13813  H -1.42937 0.7545 -3.33303  H -1.54401 -1.33375 -1.68517  H -0.63068 -2.82568 -1.50074  H -1.50532 -2.19282 0.68883  H 0.24912 -2.02381 0.75052  H 0.03106 0.47186 0.36134  H -1.7288 0.30637 0.27819  H -1.75993 -0.60547 2.64326  H -0.0028 -0.46185 2.72808  H -0.18286 2.03832 2.33879  H -1.9426 1.89869 2.24682  H -1.23011 2.61402 4.53813  H -1.99772 1.02598 4.62853  H -0.23895 1.16409 4.72004  33  Energy = -693.571868 a.u. #Cluster: 3  C 2.25301 0.27827 -2.41026  C 2.08247 1.68369 -2.8277  O 0.78633 2.23206 -2.76424  C -0.35851 1.4444 -2.54261  C -0.17402 -0.02879 -2.17281  C 1.22727 -0.51807 -2.09066  O 2.92891 2.46373 -3.17165  O -0.54329 0.41521 -3.54084  C -1.49249 2.34673 -2.19926  C -1.24704 -0.81645 -1.46495  C -1.37062 -0.45718 0.0205  C -0.18997 -0.96084 0.86019  C -0.4098 -0.68275 2.35326  C 0.77477 -1.17509 3.1965  C 0.55972 -0.90244 4.6832  H 3.29611 -0.05086 -2.37206  H 1.36845 -1.54892 -1.75684  H -1.51857 2.57477 -1.12433  H -1.39432 3.31206 -2.73046  H -2.45824 1.90981 -2.49346  H -2.22034 -0.63833 -1.97513  H -1.06397 -1.90459 -1.58655  H -1.47305 0.63972 0.13785  H -2.31496 -0.88782 0.41232  H -0.04563 -2.04647 0.69888  H 0.74688 -0.47443 0.52556  H -0.56054 0.40157 2.51902  H -1.34182 -1.17117 2.69759  H 0.92823 -2.26011 3.03464  H 1.70839 -0.68343 2.85874  H 1.40975 -1.25654 5.27764  H 0.44369 0.16858 4.88571  H -0.3368 -1.40631 5.06224  33  Energy = -693.573612 a.u. #Cluster: 4  C -0.72874 3.72636 -1.79471  C -2.08603 3.4996 -2.32919  O -2.52846 2.1663 -2.44692  C -1.66149 1.06754 -2.30538  C -0.23222 1.32045 -1.82179  C 0.13066 2.73402 -1.53968  O -2.91117 4.31841 -2.63178  O -0.56391 1.08298 -3.24752  C -2.47706 -0.16866 -2.1486  C 0.59737 0.23723 -1.18222  C 0.35935 0.16771 0.33103  C 1.19651 -0.94593 0.97308  C 0.97064 -1.01252 2.4894  C 1.80547 -2.11585 3.15576  C 1.27077 -3.51849 2.87596  H -0.49324 4.77946 -1.61084  H 1.12176 2.91083 -1.11544  H -2.51179 -0.50587 -1.10317  H -3.52029 0.00754 -2.47155  H -2.08168 -0.98895 -2.76866  H 0.36576 -0.74074 -1.657  H 1.67233 0.40834 -1.40211  H 0.60374 1.14103 0.8002  H -0.71595 -0.00185 0.53717  H 0.94491 -1.92298 0.51532  H 2.27141 -0.78427 0.76112  H 1.22484 -0.03662 2.94834  H -0.10349 -1.17484 2.70531  H 2.85995 -2.04476 2.82378  H 1.82552 -1.94194 4.25021  H 1.87755 -4.28073 3.37808  H 0.23903 -3.63482 3.22765  H 1.27796 -3.74796 1.80419  33  Energy = -693.573544 a.u. #Cluster: 5  C -0.79153 3.48018 -1.98757  C -2.16772 3.30923 -2.49399  O -2.68882 2.00061 -2.54162  C -1.88468 0.8602 -2.36249  C -0.43547 1.05062 -1.91084  C 0.01278 2.45171 -1.69838  O -2.94799 4.16033 -2.82529  O -0.80421 0.77008 -3.31965  C -2.76804 -0.31745 -2.13846  C 0.34077 -0.05015 -1.23501  C 0.11481 -0.0463 0.28166  C 0.91107 -1.16592 0.96381  C 0.67422 -1.17745 2.47966  C 1.46937 -2.28463 3.18675  C 2.96291 -1.98061 3.27522  H -0.49286 4.52478 -1.85499  H 1.01896 2.58917 -1.29525  H -2.76954 -0.63446 -1.08684  H -3.81325 -0.08049 -2.41256  H -2.45809 -1.17172 -2.76088  H 0.05153 -1.03197 -1.6685  H 1.42118 0.05304 -1.47012  H 0.405 0.93437 0.7076  H -0.9646 -0.16062 0.50313  H 0.63313 -2.14766 0.53382  H 1.99235 -1.04395 0.75639  H 0.93992 -0.19327 2.9117  H -0.40482 -1.31501 2.68773  H 1.06226 -2.42546 4.20837  H 1.31423 -3.25128 2.66846  H 3.49966 -2.77992 3.79865  H 3.41407 -1.87824 2.28181  H 3.1534 -1.04774 3.8177 |
| --- |

**Table S3**. Important thermodynamic parameters and Boltzmann distributions of the optimized **3** at B3LYP/6-31G(d) level in gas phase.

| Conformations | Energy (a.u) | Δ G(kcal/mol) | % |
| --- | --- | --- | --- |
| 1 | -693.601532 | 0.17 | 23.78% |
| 2 | -693.596339 | 3.42 | 0.10% |
| 3 | -693.601797 | 0.00 | 31.49% |
| 4 | -693.601013 | 0.49 | 13.73% |
| 5 | -693.600549 | 0.78 | 8.40% |
| 6 | -693.601177 | 0.39 | 16.33% |
| 7 | -693.600259 | 0.97 | 6.18% |

**Table S4**. The coordinates for the lowest energy conformers of **3**

| 33  Energy = -693.601532 a.u. #Cluster: 1  C 2.85064 -1.58836 2.08508  C 2.27351 -2.50567 3.03261  O 0.92338 -2.95408 2.7165  C 0.269 -2.53377 1.60276  C 0.84597 -1.65977 0.71075  C 2.17053 -1.18413 0.98017  C 0.12041 -1.19119 -0.51352  C -0.952 -0.13415 -0.17283  C -1.03701 1.00079 -1.20242  C 0.1091 2.0053 -1.0413  C 0.03082 3.14822 -2.06403  C -1.08388 4.14456 -1.75591  C -1.0767 -3.14388 1.5359  O 2.67023 -2.99478 4.05354  O -2.23722 -0.77943 -0.0733  H 3.85944 -1.24429 2.31432  H 2.62392 -0.48476 0.27197  H 0.84057 -0.78633 -1.25321  H -0.35821 -2.05219 -1.0256  H -0.82437 0.2616 0.86228  H -1.05278 0.60006 -2.23259  H -2.00923 1.52289 -1.07328  H 0.10105 2.42337 -0.01468  H 1.08178 1.4862 -1.14721  H 1.00317 3.68076 -2.08593  H -0.10616 2.73621 -3.08312  H -1.13704 4.92937 -2.51877  H -2.06463 3.65693 -1.71843  H -0.92971 4.6369 -0.78882  H -1.84331 -2.39478 1.23836  H -1.11579 -3.96021 0.79917  H -1.38517 -3.56885 2.5063  H -2.5603 -1.07254 -0.94476  33  Energy = -693.596339 a.u. #Cluster: 2  C -3.2592 -2.8341 0.58329  C -2.59225 -3.8144 1.39941  O -1.26437 -3.43411 1.86305  C -0.70831 -2.23579 1.5459  C -1.37049 -1.31359 0.76861  C -2.67692 -1.64326 0.28204  C -0.75434 0.00644 0.41654  C 0.31167 -0.13681 -0.6914  C 0.29568 0.99488 -1.73046  C 0.25136 2.42207 -1.17332  C 1.56012 2.88235 -0.51524  C 2.68439 3.11342 -1.51982  C 0.63864 -2.11846 2.14476  O -2.89837 -4.90588 1.79234  O 1.61044 -0.25163 -0.08356  H -4.25168 -3.11106 0.22792  H -3.19807 -0.91132 -0.34149  H -1.54097 0.72344 0.10588  H -0.28849 0.4624 1.31546  H 0.22038 -1.12658 -1.20296  H 1.19289 0.88113 -2.37653  H -0.57181 0.84363 -2.40393  H -0.00226 3.11733 -2.0003  H -0.57797 2.52021 -0.44523  H 1.37294 3.81577 0.05302  H 1.88578 2.13278 0.2434  H 3.60351 3.44372 -1.02215  H 2.92414 2.19935 -2.07728  H 2.41757 3.88115 -2.25546  H 1.36679 -1.70577 1.41086  H 0.6397 -1.44283 3.01291  H 1.02484 -3.0922 2.48888  H 1.95465 0.63771 0.18489  33  Energy = -693.601797 a.u. #Cluster: 3  C 3.07906 -1.01228 -0.66301  C 2.85267 -2.00481 -1.68204  O 1.61597 -2.76256 -1.54387  C 0.74871 -2.54144 -0.52097  C 0.98291 -1.57925 0.43237  C 2.19034 -0.80983 0.34429  C 0.01949 -1.33204 1.55165  C -1.08973 -0.31405 1.20306  C -0.5395 1.08452 0.89583  C -1.672 2.09563 0.68088  C -1.15762 3.46009 0.20293  C -0.73159 3.45422 -1.2632  C -0.40877 -3.45924 -0.62311  O 3.47395 -2.35169 -2.64739  O -1.87061 -0.81083 0.1037  H 4.00568 -0.44472 -0.75452  H 2.37871 -0.05317 1.11152  H -0.46583 -2.28584 1.85245  H 0.55957 -0.98914 2.45745  H -1.84812 -0.28594 2.02169  H 0.11764 1.42122 1.7195  H 0.10717 1.04861 -0.00371  H -2.40046 1.68604 -0.04953  H -2.24065 2.22544 1.62233  H -1.95399 4.21748 0.34994  H -0.31222 3.7902 0.83754  H -0.37273 4.44123 -1.5762  H 0.07615 2.73766 -1.44846  H -1.56419 3.18442 -1.9232  H -0.30838 -4.3104 0.06968  H -1.36002 -2.9458 -0.3759  H -0.50758 -3.88588 -1.63612  H -1.40402 -0.66783 -0.74555  33  Energy = -693.571147 a.u. #Cluster: 4  C 2.59103 -1.82839 0.20895  C 3.60554 -0.94312 -0.3165  O 3.25794 0.41979 -0.28941  C 2.07219 0.90675 0.16652  C 1.10777 0.07514 0.66019  C 1.41162 -1.33008 0.66806  C -0.24744 0.53389 1.14994  C -1.36392 0.43627 0.08396  C -2.74685 0.73088 0.68297  C -3.92428 0.62549 -0.30374  C -4.16751 -0.76822 -0.91068  C -4.52109 -1.85748 0.11089  C 2.03515 2.39642 0.02477  O 4.69582 -1.23479 -0.75913  O -1.07799 1.27679 -1.04021  H 2.82473 -2.88694 0.21816  H 0.6633 -2.01547 1.06359  H -0.53969 -0.08638 2.00864  H -0.20874 1.56461 1.52375  H -1.35207 -0.57548 -0.33757  H -2.90765 0.0565 1.5349  H -2.73055 1.74775 1.10894  H -4.83593 0.94738 0.22033  H -3.76864 1.34055 -1.12138  H -4.98932 -0.68136 -1.63437  H -3.29055 -1.07832 -1.49465  H -4.75657 -2.80367 -0.39069  H -3.69714 -2.05582 0.80747  H -5.39693 -1.5711 0.70812  H 1.20118 2.83999 0.57103  H 2.972 2.82825 0.39377  H 1.93929 2.67004 -1.03335  H -1.24605 2.19996 -0.78879  33  Energy = -693.600549 a.u. #Cluster: 5  C 2.79054 -0.9074 -1.48442  C 2.59297 -1.99559 -2.40721  O 1.4452 -2.84768 -2.12259  C 0.63248 -2.62427 -1.05675  C 0.83818 -1.57257 -0.19591  C 1.95683 -0.70557 -0.43101  C -0.06163 -1.32929 0.97601  C -1.2788 -0.43577 0.65366  C -0.91871 1.01022 0.28648  C -0.46908 1.8219 1.50515  C -0.1201 3.27326 1.14313  C -1.35032 4.11955 0.82691  C -0.43969 -3.64453 -1.00612  O 3.17873 -2.36309 -3.38688  O -2.05031 -1.02068 -0.40871  H 3.64933 -0.26675 -1.68734  H 2.12147 0.12535 0.26199  H -0.43333 -2.29693 1.37665  H 0.51016 -0.87398 1.81122  H -2.0075 -0.46794 1.49914  H -0.13913 1.03015 -0.49791  H -1.80953 1.48849 -0.17351  H -1.26306 1.81261 2.27832  H 0.41326 1.3418 1.97238  H 0.43435 3.73239 1.98648  H 0.57656 3.29132 0.28199  H -1.07018 5.15114 0.58469  H -1.90628 3.72145 -0.03004  H -2.04428 4.15842 1.67418  H -0.2347 -4.40514 -0.23564  H -1.42245 -3.19211 -0.766  H -0.54037 -4.18296 -1.96415  H -1.58062 -0.9458 -1.26418  33  Energy = -693.601177 a.u. #Cluster: 6  C -2.91747 1.65855 2.45054  C -2.03066 1.30111 3.52855  O -1.02428 0.30285 3.1957  C -0.93284 -0.23929 1.95124  C -1.77937 0.12964 0.93615  C -2.79889 1.10261 1.21766  C -1.6828 -0.44446 -0.44217  C -1.09365 0.5697 -1.44572  C 0.3989 0.87315 -1.25691  C 1.30038 -0.35978 -1.37368  C 2.78121 0.02833 -1.47727  C 3.6839 -1.20065 -1.54068  C 0.16503 -1.23453 1.91943  O -1.93627 1.65482 4.67056  O -1.35325 0.08261 -2.77127  H -3.68289 2.39675 2.69324  H -3.48103 1.38276 0.40873  H -1.09957 -1.38443 -0.45324  H -2.69247 -0.74251 -0.80453  H -1.68543 1.51679 -1.43425  H 0.69656 1.6266 -2.01639  H 0.54939 1.36298 -0.27454  H 1.15 -1.02021 -0.48825  H 1.00781 -0.96112 -2.25772  H 2.94387 0.65766 -2.37506  H 3.06792 0.66001 -0.61328  H 4.73889 -0.91112 -1.61928  H 3.58524 -1.82648 -0.64689  H 3.45509 -1.82838 -2.40973  H -0.22707 -2.26366 1.93472  H 0.79526 -1.12523 1.01161  H 0.82753 -1.13455 2.7978  H -0.72905 -0.63055 -3.01505  33  Energy = -693.600259 a.u. #Cluster: 7  C 2.56112 3.15198 1.04773  C 1.78867 4.07533 1.83679  O 0.46841 3.59967 2.22993  C 0.01245 2.37029 1.87531  C 0.77389 1.50461 1.12443  C 2.0776 1.92799 0.70755  C 0.26805 0.15026 0.72979  C -0.74513 0.2345 -0.43244  C -0.60383 -0.90025 -1.45654  C -0.76884 -2.29661 -0.84565  C -0.85113 -3.39779 -1.91228  C 0.48795 -3.66579 -2.59392  C -1.35124 2.15449 2.4052  O 1.99832 5.18059 2.25317  O -2.08072 0.25622 0.10712  H 3.54893 3.49982 0.7456  H 2.67824 1.24034 0.10544  H 1.11671 -0.50816 0.45529  H -0.21098 -0.34683 1.59937  H -0.69334 1.22886 -0.93791  H -1.36319 -0.75108 -2.25271  H 0.37547 -0.82254 -1.96695  H 0.06835 -2.51172 -0.15344  H -1.68934 -2.32071 -0.2261  H -1.21694 -4.332 -1.44004  H -1.61156 -3.13159 -2.67322  H 0.40122 -4.47105 -3.33284  H 0.85937 -2.7794 -3.12024  H 1.25691 -3.96532 -1.87302  H -2.01307 1.70335 1.63284  H -1.34862 1.47003 3.26648  H -1.81942 3.09604 2.7379  H -2.37431 -0.64353 0.35738 |
| --- |

**Table S5**. Important thermodynamic parameters and Boltzmann distributions of the optimized **4** at B3LYP/6-31G(d) level in gas phase.

| Conformations | Energy (a.u) | Δ G(kcal/mol) | % |
| --- | --- | --- | --- |
| 1 | -693.600052 | 2.95 | 0.68% |
| 2 | -693.597929 | 4.29 | 0.07% |
| 3 | -693.604761 | 0.00 | 99.25% |

**Table S6**. The coordinates for the lowest energy conformers of **4**

| 33  Energy = -693.600052 a.u. #Cluster: 1  C -2.15556 -2.04501 1.60138  C -1.61178 -2.91914 0.59522  O -1.22941 -2.27035 -0.6487  C -1.37763 -0.92845 -0.8362  C -1.88506 -0.11454 0.14599  C -2.28318 -0.70855 1.38878  C -2.0072 1.37043 -0.03427  C -0.82696 2.14376 0.57904  C 0.51191 1.88133 -0.12483  C 1.36201 0.82407 0.59057  C 2.53414 0.36529 -0.29252  C 3.52799 -0.50365 0.47142  C -0.90623 -0.5535 -2.18747  O -1.39107 -4.09873 0.56176  O 2.02549 -0.35497 -1.43152  H -2.45594 -2.52471 2.53284  H -2.69296 -0.05575 2.16511  H -2.10137 1.62598 -1.10989  H -2.95388 1.7242 0.42619  H -1.05554 3.22735 0.53411  H -0.74572 1.9052 1.65717  H 0.33774 1.5524 -1.17469  H 1.08293 2.82652 -0.20063  H 1.74404 1.22446 1.54781  H 0.73242 -0.04619 0.86153  H 3.04214 1.22703 -0.78513  H 4.27257 -0.93246 -0.2132  H 4.07267 0.07278 1.2269  H 3.04357 -1.34124 0.98553  H -1.67794 -0.0294 -2.76805  H -0.01681 0.1192 -2.11809  H -0.59194 -1.43233 -2.77622  H 1.67656 -1.23102 -1.16992  33  Energy = -693.597929 a.u. #Cluster: 2  C -2.52309 -2.58085 -2.17549  C -2.2217 -3.57793 -1.18021  O -1.60399 -3.07728 0.0323  C -1.31865 -1.75401 0.20275  C -1.6 -0.82413 -0.76579  C -2.22654 -1.27003 -1.97758  C -1.28237 0.63352 -0.59656  C -0.18018 1.11834 -1.55117  C 1.23256 0.73568 -1.08741  C 1.83921 1.71508 -0.07148  C 1.79811 1.20106 1.37566  C 2.81856 1.90542 2.26682  C -0.71073 -1.54446 1.53593  O -2.37876 -4.76806 -1.13943  O 0.48225 1.37456 1.93954  H -2.99998 -2.94761 -3.08376  H -2.46261 -0.52252 -2.74042  H -2.20383 1.22931 -0.75588  H -0.97022 0.84419 0.45583  H -0.35169 0.70241 -2.56354  H -0.25603 2.21659 -1.66777  H 1.21736 -0.29336 -0.67486  H 1.89625 0.68365 -1.97359  H 2.89094 1.92101 -0.35373  H 1.32821 2.69495 -0.13196  H 1.91404 0.0928 1.42634  H 2.65195 1.64753 3.32144  H 3.84411 1.61063 2.01828  H 2.76372 2.99599 2.18932  H 0.24113 -2.08859 1.63613  H -0.50882 -0.47191 1.74129  H -1.36525 -1.91969 2.33956  H 0.24606 2.31772 2.00944  33  Energy = -693.604761 a.u. #Cluster: 3  C -3.66886 2.21191 -0.14296  C -4.60724 1.55925 -1.01961  O -4.1832 0.26259 -1.52646  C -2.98456 -0.28322 -1.18444  C -2.11058 0.35394 -0.34101  C -2.48021 1.63934 0.17973  C -0.78207 -0.23768 0.03058  C 0.33443 0.28577 -0.8874  C 1.69449 -0.35067 -0.58321  C 2.3063 0.14821 0.7308  C 3.63294 -0.56993 1.02878  C 4.29554 -0.04492 2.30019  C -2.82445 -1.60083 -1.84524  O -5.69178 1.86031 -1.4351  O 3.41876 -1.98628 1.14099  H -3.97684 3.18618 0.23694  H -1.7759 2.14757 0.84442  H -0.54421 -0.00158 1.0884  H -0.81905 -1.34523 -0.01229  H 0.06615 0.08717 -1.94403  H 0.40114 1.38783 -0.80497  H 1.60845 -1.45729 -0.54887  H 2.39014 -0.13945 -1.42039  H 2.47702 1.24003 0.68385  H 1.59444 -0.0058 1.56274  H 4.32979 -0.52285 0.15942  H 5.16058 -0.6673 2.56574  H 4.6571 0.9811 2.17435  H 3.6189 -0.05193 3.16004  H -1.84564 -1.69591 -2.33916  H -2.91626 -2.42699 -1.12168  H -3.60203 -1.76446 -2.61392  H 2.82671 -2.19886 1.88374 |
| --- |

**Table S7**. Important thermodynamic parameters and Boltzmann distributions of the optimized **6** at B3LYP/6-31G(d) level in gas phase.

| Conformations | Energy (a.u) | Δ G(kcal/mol) | % |
| --- | --- | --- | --- |
| 1 | -693.607528 | 0.00 | 75.39% |
| 2 | -693.605929 | 1.00 | 13.86% |
| 3 | -693.604353 | 1.99 | 2.61% |
| 4 | -693.605427 | 1.32 | 8.14% |

**Table S8**. The coordinates for the lowest energy conformers of **6**

| 33  Energy = -693.607528 a.u. #Cluster: 1  C 3.24244 -2.19035 -1.50261  C 4.18113 -1.39394 -0.66242  O 3.50273 -0.20539 -0.28944  C 2.14036 -0.22481 -0.84556  C 2.08284 -1.53463 -1.61964  C 1.18934 -0.1467 0.35005  C -0.28566 -0.21763 -0.05638  C 2.92722 2.14163 -1.50586  C 1.99721 1.01222 -1.77457  O 5.30376 -1.55116 -0.28404  O 1.14441 1.01429 -2.62954  C -1.20286 0.06135 1.14103  C -2.6814 -0.0261 0.74044  C -3.60406 0.25561 1.93457  C -5.07677 0.1702 1.54074  H 3.5454 -3.14033 -1.91504  H 1.19034 -1.80529 -2.16594  H 1.39874 0.78679 0.91407  H 1.43499 -0.96477 1.05996  H -0.51591 -1.21444 -0.48055  H -0.49782 0.50614 -0.87145  H 2.58962 2.73836 -0.64361  H 3.94522 1.7929 -1.25122  H 3.00106 2.82395 -2.36578  H -0.98673 1.06391 1.55871  H -0.99115 -0.65725 1.95646  H -2.90353 -1.02757 0.32407  H -2.89589 0.69116 -0.07573  H -3.3867 1.25921 2.35048  H -3.39375 -0.4624 2.75166  H -5.72831 0.37424 2.39814  H -5.33723 -0.82458 1.16091  H -5.32902 0.89541 0.75809  33  Energy = -693.605929 a.u. #Cluster: 2  C 2.58095 -1.76383 -2.21026  C 3.53976 -0.93068 -1.4306  O 2.85036 0.24569 -1.04029  C 1.46253 0.18298 -1.52566  C 1.40018 -1.13965 -2.27721  C 0.57122 0.25574 -0.2844  C -0.91959 0.12502 -0.61158  C 2.15959 2.5534 -2.27177  C 1.23904 1.4007 -2.46374  O 4.6839 -1.05353 -1.10804  O 0.33528 1.3715 -3.26454  C -1.80787 0.4597 0.59333  C -1.74072 -0.60708 1.69369  C -2.67396 -0.26608 2.86398  C -2.6091 -1.32332 3.96349  H 2.88742 -2.71205 -2.62401  H 0.48843 -1.44147 -2.77299  H 0.77493 1.20727 0.25043  H 0.88057 -0.5373 0.42921  H -1.1421 -0.89988 -0.96819  H -1.1852 0.79502 -1.45789  H 1.84812 3.17199 -1.41546  H 3.19435 2.23153 -2.05186  H 2.18253 3.20919 -3.15469  H -2.85544 0.56891 0.24615  H -1.53018 1.44821 1.00803  H -0.70207 -0.70845 2.06232  H -2.00869 -1.5971 1.27739  H -3.71573 -0.16973 2.50009  H -2.40757 0.72426 3.28252  H -3.27876 -1.07231 4.79402  H -1.59789 -1.41711 4.37582  H -2.9043 -2.31155 3.59272  33  Energy = -693.604353 a.u. #Cluster: 3  C 2.98751 0.46565 -1.66091  C 3.51549 -0.72865 -0.94237  O 2.8384 -0.80167 0.30147  C 1.86664 0.29985 0.39479  C 2.05577 1.06201 -0.90954  C 0.49385 -0.35423 0.55975  C -0.65053 0.66261 0.61318  C 3.06156 0.47195 2.67526  C 2.24787 1.15524 1.634  O 4.33771 -1.55906 -1.19283  O 1.85496 2.29478 1.70959  C -1.98578 0.00763 0.98976  C -2.57536 -0.87384 -0.11929  C -3.12767 -0.05276 -1.29271  C -3.70042 -0.94786 -2.38896  H 3.36605 0.73216 -2.63549  H 1.47947 1.95485 -1.10674  H 0.50335 -0.9855 1.47293  H 0.33159 -1.07013 -0.27449  H -0.74984 1.17431 -0.36491  H -0.41592 1.4652 1.34451  H 2.43642 -0.18199 3.30359  H 3.83602 -0.18401 2.23626  H 3.55677 1.1912 3.3442  H -2.712 0.80155 1.25829  H -1.86107 -0.59729 1.90955  H -3.3844 -1.50048 0.30538  H -1.81171 -1.58636 -0.48599  H -2.32773 0.58842 -1.7127  H -3.91058 0.64173 -0.92949  H -4.09949 -0.35272 -3.21804  H -4.51774 -1.57451 -2.01409  H -2.93918 -1.61807 -2.80394  33  Energy = -693.605427 a.u. #Cluster: 4  C -2.53524 0.78383 3.12328  C -2.46416 -0.46719 2.31396  O -1.464 -0.27456 1.33093  C -0.86421 1.05771 1.49237  C -1.63681 1.66004 2.66333  C 0.62673 0.8928 1.79561  C 1.46779 0.39913 0.61199  C -1.59507 1.10202 -0.99002  C -1.11547 1.86677 0.18944  O -3.0521 -1.50795 2.33602  O -0.89987 3.05552 0.19518  C 1.23852 -1.0856 0.30077  C 1.83708 -1.49441 -1.05127  C 0.85396 -1.2794 -2.21076  C 1.53479 -1.40122 -3.57049  H -3.24095 0.87478 3.9344  H -1.42298 2.6656 2.99674  H 0.74068 0.18968 2.64809  H 1.02517 1.87055 2.14292  H 2.54003 0.5645 0.84507  H 1.27381 1.01615 -0.28669  H -0.75708 0.53688 -1.46163  H -2.34439 0.33778 -0.72196  H -2.02265 1.75842 -1.76087  H 0.15067 -1.31754 0.32795  H 1.6785 -1.70515 1.10586  H 2.13241 -2.56158 -1.01688  H 2.77622 -0.93952 -1.23925  H 0.37092 -0.27844 -2.11856  H 0.02884 -2.01618 -2.1367  H 0.81509 -1.28218 -4.38825  H 2.01131 -2.38074 -3.69576  H 2.31243 -0.6405 -3.70483 |
| --- |

**Table S9**. Important thermodynamic parameters and Boltzmann distributions of the optimized **7** at B3LYP/6-31G(d) level in gas phase.

| Conformations | Energy (a.u) | Δ G(kcal/mol) | % |
| --- | --- | --- | --- |
| 1 | -694.791561 | 0 | 48.06% |
| 2 | -694.790585 | 0.61 | 17.09% |
| 3 | -694.79045 | 0.70 | 14.82% |
| 4 | -694.79015 | 0.89 | 10.78% |
| 5 | -694.790005 | 0.98 | 9.25% |

**Table S10**. The coordinates for the lowest energy conformers of **7**.

| 35  Energy = -694.79156100 a.u. #Cluster: 1  C -0.20085800 -2.58986100 -2.14871700  C -0.16123200 -3.25610500 -0.82771700  O 0.75258400 -2.57182400 -0.04765800  C 1.34540600 -1.48227400 -0.80308600  C 0.66428600 -1.57541300 -2.14157400  C 1.06334300 -0.16076600 -0.07024600  C -0.41709900 0.11433000 0.20661800  C 3.66062000 -0.86726200 -1.76576100  C 2.86807900 -1.79991900 -0.86041000  O -0.76381800 -4.21565400 -0.41967100  O 3.08447200 -3.11632100 -1.33273500  C -0.63383800 1.42749200 0.96418000  C -2.10991100 1.71604100 1.25186000  C -2.33639100 3.02969500 2.00640300  C -3.81455200 3.31006000 2.28706200  H -0.84716700 -2.93027700 -2.94645600  H 0.87502600 -0.88638800 -2.95090300  H 1.48393400 0.65757700 -0.66790100  H 1.61751800 -0.17526700 0.87700000  H -0.83527100 -0.71700800 0.78586900  H -0.97317600 0.14455000 -0.73941600  H 4.71765000 -1.14408100 -1.72630400  H 3.32903700 -0.96383700 -2.80443000  H 3.56545400 0.17725700 -1.45561400  H 3.23407000 -1.70047400 0.17437100  H 2.60254600 -3.70863900 -0.73272500  H -0.20619400 2.25991000 0.38588900  H -0.07741500 1.39804300 1.91223100  H -2.53590600 0.88482800 1.83206200  H -2.66709100 1.74130400 0.30382500  H -1.90786600 3.85848900 1.42537300  H -1.78026700 3.00252000 2.95392100  H -4.38802900 3.37301200 1.35422100  H -3.94911300 4.25430900 2.82631100  H -4.25880900 2.51196300 2.89411000  35  Energy = -694.79058500 a.u. #Cluster: 2  C -2.08888000 -1.20957400 2.47516300  C -2.64635600 0.11634400 2.12648300  O -1.58057100 0.95207700 1.84903900  C -0.32501300 0.24277200 2.01979100  C -0.75849700 -1.14165100 2.41970100  C 0.44776300 0.27596100 0.69116600  C -0.32977800 -0.25921200 -0.51423900  C 1.70706300 0.31871500 3.60997200  C 0.42903600 1.00470100 3.14837000  O -3.78918200 0.49253700 2.07355700  O -0.39678200 1.15081700 4.28839700  C 0.47909100 -0.17845900 -1.81229800  C -0.29656000 -0.70486500 -3.02472100  C 0.50593600 -0.71326900 -4.33335500  C 0.91003600 0.67924900 -4.82774700  H -2.71935200 -2.05081700 2.72971800  H -0.05124000 -1.93869900 2.61635200  H 1.37175300 -0.30169800 0.81883800  H 0.74953000 1.31412400 0.50163100  H -1.25834100 0.31268900 -0.62502600  H -0.62609600 -1.30074100 -0.33363700  H 2.18211100 0.93035300 4.38183200  H 1.48369600 -0.65869400 4.04918600  H 2.41645400 0.18766900 2.78795700  H 0.67774300 1.99267800 2.72807100  H -1.19427900 1.62071100 3.99458000  H 1.41152500 -0.75229300 -1.70184300  H 0.77990900 0.86373400 -1.98298300  H -1.20592000 -0.10151600 -3.15979800  H -0.63780900 -1.72696100 -2.80871700  H -0.09173600 -1.20915500 -5.10932700  H 1.40606600 -1.33027700 -4.20047700  H 1.42387300 0.62124700 -5.79370200  H 1.58552800 1.18078900 -4.12601000  H 0.02927900 1.32068500 -4.95594300  35  Energy = -694.79045000 a.u. #Cluster: 3  C -1.00075000 -3.02734000 -1.65849600  C -0.97235900 -3.63606100 -0.30994100  O -0.04625400 -2.93481500 0.43988700  C 0.56801100 -1.89236700 -0.36287100  C -0.11650200 -2.03016000 -1.69594500  C 0.31701800 -0.53359200 0.30975400  C -1.16079400 -0.20487600 0.54648100  C 2.89264400 -1.37350700 -1.35969100  C 2.08403800 -2.24450200 -0.40834200  O -1.59180200 -4.56602600 0.13933700  O 2.27084900 -3.58597700 -0.81861000  C -1.37336300 1.10193800 1.32213000  C -0.89516800 2.36138200 0.59114400  C -1.21686400 3.65378600 1.34819500  C -0.73174600 4.91026500 0.62157200  H -1.65420100 -3.38974100 -2.44056600  H 0.10622400 -1.38096300 -2.53448700  H 0.77988400 0.23896800 -0.31378500  H 0.85146500 -0.52809400 1.26873000  H -1.62022000 -1.03056500 1.10063900  H -1.68444300 -0.14496400 -0.41671600  H 3.94329700 -1.67245100 -1.31179600  H 2.55318900 -1.51129900 -2.39109000  H 2.82323900 -0.31359600 -1.09903700  H 2.45591900 -2.10401800 0.61951700  H 1.77936800 -4.13943200 -0.18990200  H -0.86784200 1.03327900 2.29639800  H -2.44429100 1.20579600 1.54255200  H -1.35837100 2.39985200 -0.40639200  H 0.18950000 2.31103500 0.42163400  H -0.76328600 3.60819900 2.34817800  H -2.30204000 3.71659800 1.50917600  H 0.35565700 4.88900400 0.47907800  H -0.97679300 5.81858200 1.18304600  H -1.19322200 4.99567200 -0.36980000  35  Energy = -694.79015000 a.u. #Cluster: 4  C 0.86740400 -2.66626400 -2.05924700  C 1.02676300 -3.21864100 -0.69315900  O 1.57325700 -2.22394900 0.09147900  C 1.80395900 -1.03340200 -0.69409600  C 1.30700300 -1.40698000 -2.06763500  C 1.04076400 0.14107900 -0.06671200  C -0.48295700 -0.00191000 -0.09728900  C 3.95552200 -0.53882200 0.63711400  C 3.33603500 -0.81028500 -0.72941600  O 0.76026100 -4.31090500 -0.26059400  O 3.54095400 0.27182200 -1.63873500  C -1.18590500 1.13811200 0.64507100  C -2.71277800 1.03103900 0.60838600  C -3.41884800 2.16968000 1.35101400  C -4.94459300 2.06092700 1.30138300  H 0.45622300 -3.25092100 -2.87131500  H 1.34900300 -0.71419800 -2.89846900  H 1.35251200 1.05275300 -0.58905500  H 1.37080100 0.23859800 0.97353400  H -0.76746000 -0.96141300 0.35249600  H -0.83638000 -0.03045000 -1.13634300  H 5.04894100 -0.50609900 0.55309100  H 3.61706400 0.41988900 1.04056000  H 3.69446700 -1.33500200 1.34001500  H 3.76655700 -1.73724200 -1.13856400  H 4.48669700 0.48359100 -1.63701200  H -0.87813000 2.10076700 0.21082800  H -0.84702300 1.15408000 1.69115700  H -3.02074800 0.06820900 1.04124000  H -3.05135700 1.01570400 -0.43810800  H -3.10381000 3.13087300 0.92095100  H -3.08417000 2.18017100 2.39779300  H -5.42425700 2.88470900 1.84169500  H -5.28773300 1.12132600 1.75123100  H -5.30802800 2.08314300 0.26671900  35  Energy = -694.79000500 a.u. #Cluster: 5  C 3.05188900 -1.45785100 -2.64210900  C 1.73559300 -1.30031400 -3.30021400  O 0.77734100 -1.27404300 -2.30497000  C 1.40163500 -1.39167800 -0.99755000  C 2.86544800 -1.53242300 -1.32409800  C 1.13186000 -0.09406700 -0.21501000  C -0.34350400 0.19580400 0.08090700  C 1.37252000 -2.98838400 1.01601000  C 0.79066600 -2.66230000 -0.35199400  O 1.45339600 -1.21362100 -4.46794800  O 1.00929100 -3.79128400 -1.18165300  C -0.54688200 1.58870000 0.68489100  C -2.01414600 1.90219500 0.99064300  C -2.22456600 3.29451600 1.59369400  C -3.69452300 3.59994400 1.89109200  H 3.97470300 -1.50623600 -3.20427800  H 3.62332900 -1.65185300 -0.55901100  H 1.54920700 0.72785000 -0.80959500  H 1.70628400 -0.12273100 0.71964600  H -0.74830200 -0.55440800 0.77263200  H -0.91765900 0.10880600 -0.84936000  H 0.90378300 -3.90025100 1.39573100  H 2.45010300 -3.16931500 0.94975300  H 1.19099500 -2.18132000 1.73188200  H -0.28758900 -2.47506500 -0.25156300  H 0.59839700 -3.59012200 -2.03817400  H -0.14916800 2.34502400 -0.00695200  H 0.04503400 1.67801200 1.60775800  H -2.41300400 1.14389500 1.68050700  H -2.60389700 1.81294000 0.06693900  H -1.82355600 4.05073200 0.90448600  H -1.63554200 3.38105000 2.51750400  H -4.29958300 3.54859700 0.97784600  H -3.81846200 4.60115300 2.31843300  H -4.11108100 2.87804700 2.60412400 |
| --- |

**Table S11**. Important thermodynamic parameters and Boltzmann distributions of the optimized **8** at B3LYP/6-31G(d) level in gas phase.

| Conformations | Energy (a.u) | Δ G(kcal/mol) | % |
| --- | --- | --- | --- |
| 1 | -694.792311 | 0 | 65.31% |
| 2 | -694.790837 | 0.92 | 13.71% |
| 3 | -694.790587 | 1.08 | 10.52% |
| 4 | -694.790172 | 1.34 | 6.78% |
| 5 | -694.789597 | 1.70 | 3.69% |

**Table S12**. The coordinates for the lowest energy conformers of **8**.

| 35  Energy = -694.79231100 a.u. #Cluster: 1  C 2.30706600 2.58280100 -0.47487300  C 3.13347900 1.43605600 -0.02951400  O 2.41519000 0.76766500 0.94117300  C 1.15197900 1.44250500 1.19462000  C 1.16651400 2.58541700 0.21821000  C 0.00183900 0.45135900 0.97118000  C 0.02054600 -0.25066700 -0.38887400  C 0.01083200 2.62426300 3.19029500  C 1.25609300 1.92212000 2.67127900  O 4.22576300 1.07921400 -0.38901400  O 1.50399000 0.81299500 3.51796400  C -1.14463200 -1.23110600 -0.55088200  C -1.13546600 -1.95720400 -1.89892600  C -2.29604000 -2.94283500 -2.06584600  C -2.27701200 -3.66549300 -3.41484700  H 2.63843000 3.26587900 -1.24550900  H 0.34219500 3.28366100 0.12780200  H -0.94152800 0.99934800 1.08983900  H 0.04284900 -0.28556200 1.78044500  H 0.96933900 -0.78710500 -0.50508600  H -0.01517600 0.49352600 -1.19593100  H -0.23424500 3.50136900 2.58246600  H -0.84639500 1.94517400 3.19733000  H 0.18637300 2.95533000 4.21762600  H 2.10770400 2.62075100 2.70050100  H 2.28693500 0.36444200 3.15555500  H -2.09690100 -0.69295200 -0.43359900  H -1.11215900 -1.97135100 0.26153400  H -0.18284400 -2.49398800 -2.01530400  H -1.16765100 -1.21684400 -2.71182500  H -3.24735900 -2.40470600 -1.94942000  H -2.26246300 -3.68083300 -1.25219900  H -2.34157100 -2.95201900 -4.24542600  H -3.11592000 -4.36408300 -3.50845600  H -1.34977900 -4.23712800 -3.54228900  35  Energy = -694.79083700 a.u. #Cluster: 2  C 2.11183200 2.22237500 -1.03509100  C 2.86101900 0.94909300 -1.16042900  O 2.57146300 0.18898900 -0.04620700  C 1.65967000 0.89803900 0.82060100  C 1.42232900 2.20277500 0.10658500  C 0.38281300 0.05863300 0.99380500  C -0.39452800 -0.19926400 -0.29836100  C 3.73791700 1.76151500 2.07475200  C 2.36781000 1.10144100 2.18246200  O 3.60423700 0.56809300 -2.02866100  O 1.46012600 1.89053200 2.95475200  C -1.59584100 -1.12371000 -0.07999400  C -2.39850100 -1.37764000 -1.35892300  C -3.60076400 -2.30329000 -1.14908300  C -4.39872100 -2.54697000 -2.43216000  H 2.16584700 2.99808100 -1.78730500  H 0.77986200 2.97131500 0.51751300  H -0.24885100 0.56832400 1.72906800  H 0.68320500 -0.89818800 1.44011700  H 0.27590400 -0.64286200 -1.04490000  H -0.74275600 0.75223400 -0.72126900  H 4.41781600 1.15282500 1.47295800  H 3.65885900 2.75576300 1.62540800  H 4.17872800 1.86949500 3.07342600  H 2.48328800 0.10310700 2.63267500  H 1.86497900 2.03854700 3.82261100  H -2.25683600 -0.69025800 0.68496400  H -1.24698800 -2.08353100 0.32778500  H -1.73647500 -1.80888000 -2.12383100  H -2.74723100 -0.41717400 -1.76620500  H -4.25931200 -1.87196000 -0.38201700  H -3.25095700 -3.26356000 -0.74492300  H -4.78771600 -1.60566700 -2.83925200  H -5.25132300 -3.21215600 -2.25543500  H -3.77087500 -3.00607600 -3.20543400  35  Energy = -694.79058700 a.u. #Cluster: 3  C 2.01879000 1.36860500 -2.24552300  C 1.52448500 0.10767900 -2.84878600  O 1.43509400 -0.82592000 -1.83726800  C 1.84837700 -0.24471600 -0.58152000  C 2.21334300 1.17157400 -0.94071600  C 0.68172800 -0.34020600 0.41591200  C -0.57536200 0.42995700 0.00710700  C 4.20278300 -1.16725000 -1.08250500  C 3.06944700 -1.04739300 -0.06956300  O 1.23502300 -0.14511100 -3.99059000  O 3.48846600 -0.36332900 1.11352200  C -1.74320000 0.18699700 0.96767000  C -3.00477100 0.96459100 0.57792300  C -4.17737400 0.79822400 1.55498100  C -4.72599900 -0.63007300 1.63369900  H 2.17471700 2.26444900 -2.83170300  H 2.57945100 1.87285900 -0.20154600  H 1.04824100 0.00673000 1.38783700  H 0.44063600 -1.40532500 0.52752500  H -0.87069100 0.13318700 -1.00703200  H -0.35760400 1.50525200 -0.03596200  H 3.86563600 -1.68117800 -1.98649500  H 4.58885500 -0.18121900 -1.35710200  H 5.02593800 -1.75077700 -0.65205000  H 2.70346000 -2.05499900 0.18221000  H 4.25209900 -0.83943800 1.47294800  H -1.44412500 0.46933000 1.98825300  H -1.96067100 -0.88879700 0.99950700  H -3.32354700 0.65361300 -0.42762900  H -2.75260000 2.03160800 0.50160400  H -4.98642900 1.47616900 1.25289200  H -3.86233100 1.12544700 2.55603800  H -5.03144100 -0.98706900 0.64227500  H -5.60068300 -0.68197000 2.29157800  H -3.97975400 -1.33150000 2.02247100  35  Energy = -694.79017200 a.u. #Cluster: 4  C 3.18257000 1.16950400 -0.28867800  C 3.26823300 -0.27271100 -0.60990500  O 2.45361300 -0.94493700 0.28316700  C 1.83026200 -0.00953000 1.19939700  C 2.36998100 1.32510500 0.75622100  C 0.30341200 -0.11346800 1.05552800  C -0.23949600 0.29361400 -0.31717200  C 1.83601300 -1.73702200 3.11334700  C 2.32853500 -0.37410800 2.62638800  O 3.90253500 -0.84662700 -1.45768600  O 3.74470200 -0.29873300 2.67744300  C -1.74407900 0.03948600 -0.44562700  C -2.30461500 0.43191200 -1.81547500  C -3.80872200 0.17607900 -1.95172800  C -4.35866400 0.56558100 -3.32586700  H 3.73240900 1.91710100 -0.84417100  H 2.10169000 2.25023100 1.25420700  H -0.15452000 0.50437400 1.84048100  H 0.01481600 -1.14941700 1.26085700  H 0.29314700 -0.26910900 -1.09372800  H -0.03091100 1.35440200 -0.50623400  H 0.75388200 -1.75511400 3.27405800  H 2.08914800 -2.51797000 2.38777400  H 2.32764100 -1.96831800 4.06246700  H 1.97492100 0.40994400 3.30620400  H 4.07845300 -0.97155200 2.06043000  H -2.27872900 0.59420200 0.33958100  H -1.94979900 -1.02472500 -0.26021300  H -1.76858200 -0.12303700 -2.59890500  H -2.09881400 1.49614900 -2.00233800  H -4.34260900 0.73338400 -1.16917400  H -4.01264400 -0.88695500 -1.76154800  H -4.19632400 1.63128300 -3.52827800  H -5.43470400 0.37205900 -3.39764300  H -3.86277200 -0.00105300 -4.12321000  35  Energy = -694.78959700 a.u. #Cluster: 5  C 1.56663100 3.26480500 2.13066800  C 1.14170500 3.57373600 0.74424900  O 1.06607900 2.37834000 0.06016900  C 1.39617700 1.27802900 0.93623400  C 1.73253000 1.94609500 2.24366100  C 0.15923500 0.37320000 1.08495100  C -0.31702500 -0.28331000 -0.21384000  C 3.84432900 1.41413100 0.15890100  C 2.60358300 0.54125700 0.31515300  O 0.89373800 4.63269500 0.22614400  O 2.84078200 -0.57146100 1.17956800  C -1.65620200 -1.00560100 -0.03631500  C -2.15094400 -1.67881400 -1.31961000  C -3.48990500 -2.40309600 -1.14871600  C -3.97900500 -3.06702800 -2.43819100  H 1.70153800 4.03689100 2.87641200  H 2.04523300 1.37911800 3.11192400  H -0.64385900 0.99746900 1.49621000  H 0.38928400 -0.39527400 1.83034800  H 0.43288600 -1.00539700 -0.56104500  H -0.41179900 0.48218300 -0.99380300  H 3.63060500 2.28466500 -0.46744300  H 4.20917100 1.75804100 1.13114300  H 4.64455200 0.84250100 -0.32730700  H 2.28885200 0.19328500 -0.67844400  H 3.62100300 -1.04007200 0.84667900  H -2.41264600 -0.28776200 0.31235200  H -1.56178500 -1.76009400 0.75828600  H -1.39337600 -2.39498000 -1.67078100  H -2.24526700 -0.92281700 -2.11257800  H -4.24476300 -1.68729900 -0.79436600  H -3.39284200 -3.16008500 -0.35790800  H -4.11221000 -2.32626700 -3.23593300  H -4.93842700 -3.57521700 -2.29024900  H -3.25785700 -3.81183600 -2.79653000 |
| --- |
